# ARHGEF2 isoform switching couples intestinal epithelial barrier function with autophagic host defense response to pathogens

**DOI:** 10.1038/s41467-026-78030-w

**Published:** 2026-09-24

**Authors:** Shuyuan Chen, Alka Diwaker, Xi Li, Abdulmajeed Salamah, Lei Guo, Neethu S. Alex, Ashutosh Shukla, Mary-Joe Touma, Yassin EL-Najjar, Lin Xu, Jianyi Yin, Ezra Burstein, Marcel Mettlen, Andrew Lemoff, Suraj Patel, Josephine Ni, Hans-Christian Reinecker

**Affiliations:** 1 Department of Medicine, Division of Digestive and Liver Diseases, University of Texas Southwestern Medical Center, 5959 Harry Hines Boulevard, Dallas, Texas USA; 2 Department of Immunology, University of Texas Southwestern Medical Center, 5959 Harry Hines Boulevard, Dallas, Texas USA; 3 Quantitative Biomedical Research Center, Peter O’Donnell Jr. School of Public Health University of Texas Southwestern Medical Center, Dallas, Texas USA; 4 Department of Cell Biology, University of Texas Southwestern Medical Center, Dallas, TX USA; 5 Department of Biochemistry, University of Texas Southwestern Medical Center, Dallas, TX USA; 6 Center for the Genetics of Host Defense, University of Texas Southwestern Medical Center, 5959 Harry Hines Boulevard, Dallas, Texas USA; 7 Simmons Cancer Center, University of Texas Southwestern Medical Center, 5959 Harry Hines Boulevard, Dallas, Texas USA

**Keywords:** Apicobasal polarity, Macroautophagy, Mechanisms of disease

## Abstract

Intestinal epithelial integrity is essential for host defense, yet how epithelial junctional scaffolds coordinate antimicrobial immunity remains unclear. We show that the long GEF-H1 isoforms (mouse Arhgef2-207; human ARHGEF2-219) localize to adherens junctions in polarized epithelial cells through interactions with actin and Nectin-3, whereas the shorter Arhgef2-201 isoform localizes to tight junctions. Conditional deletion of epithelial Arhgef2-207 induces Arhgef2-201 expression, disrupts barrier integrity, activates autophagy, and causes small-intestinal inflammation. In human intestinal organoids, *Listeria monocytogenes* selectively depletes junction-associated ARHGEF2-219, triggering a switch to ARHGEF2-201 and RhoA- and STING-mediated autophagic signaling. This transition coincides with loss of the Na⁺/K⁺-ATPase and epithelial polarity. Here we show that a pathogen-induced ARHGEF2 isoform switch links junctional perturbation to autophagic responses and immune activation, revealing a mechanism that couples barrier disruption to cell-intrinsic antimicrobial defense.

## Abstract (web summary)

Different forms of GEF-H1 coordinate the gut’s defenses: long forms build and maintain the intestinal barrier, while barrier damage triggers a short form that helps cells capture and destroy invading bacteria such as *Listeria*.

## Introduction

Disruption of intestinal epithelial integrity and impaired microbial sensing are hallmarks of chronic inflammatory bowel diseases (IBD), including Crohn’s disease and ulcerative colitis^1,2^. Variants in multiple autophagy-related genes confer susceptibility to IBD^3^. However, the mechanisms by which autophagy preserves epithelial barrier function during antimicrobial defense and inflammation remain incompletely understood^4,5^. Beyond lysosomal degradation of cytoplasmic cargo, autophagy also functions as a signaling platform that shapes epithelial responses to infection by regulating inflammation, coordinating antimicrobial defense, and supporting cell survival^6–8^.

The guanine nucleotide exchange factor GEF-H1, encoded by ARHGEF2, is a microtubule-associated Dbl-family protein that activates RhoA and regulates intercellular junctions and proliferation^9–12^. GEF-H1 has also emerged as an innate immune regulator, coordinating macrophage responses to pathogen-associated molecular patterns (PAMPs), including double-stranded RNA and bacterial peptidoglycan^13–16^. The GEF-H1-IKKε-IRF5 signaling axis is required for defense against intracellular pathogens such as *L. monocytogenes*^16,17^, but its role in host defense activation in the intestinal epithelium remains uncharacterized.

The ARHGEF2 locus produces multiple transcripts with distinct first exons, suggesting alternative promoter–driven isoform specialization. Using isoform-specific conditional knockout mice and discriminatory antibodies, we show that ARHGEF2 isoforms are required for intestinal homeostasis, with nonredundant roles in barrier maintenance and coupling junctional stress to mucosal host defense via autophagy-related pathways.

## Results

### Distinct long ARHGEF2 isoforms are expressed in polarized intestinal epithelia

**Fig. 1:**
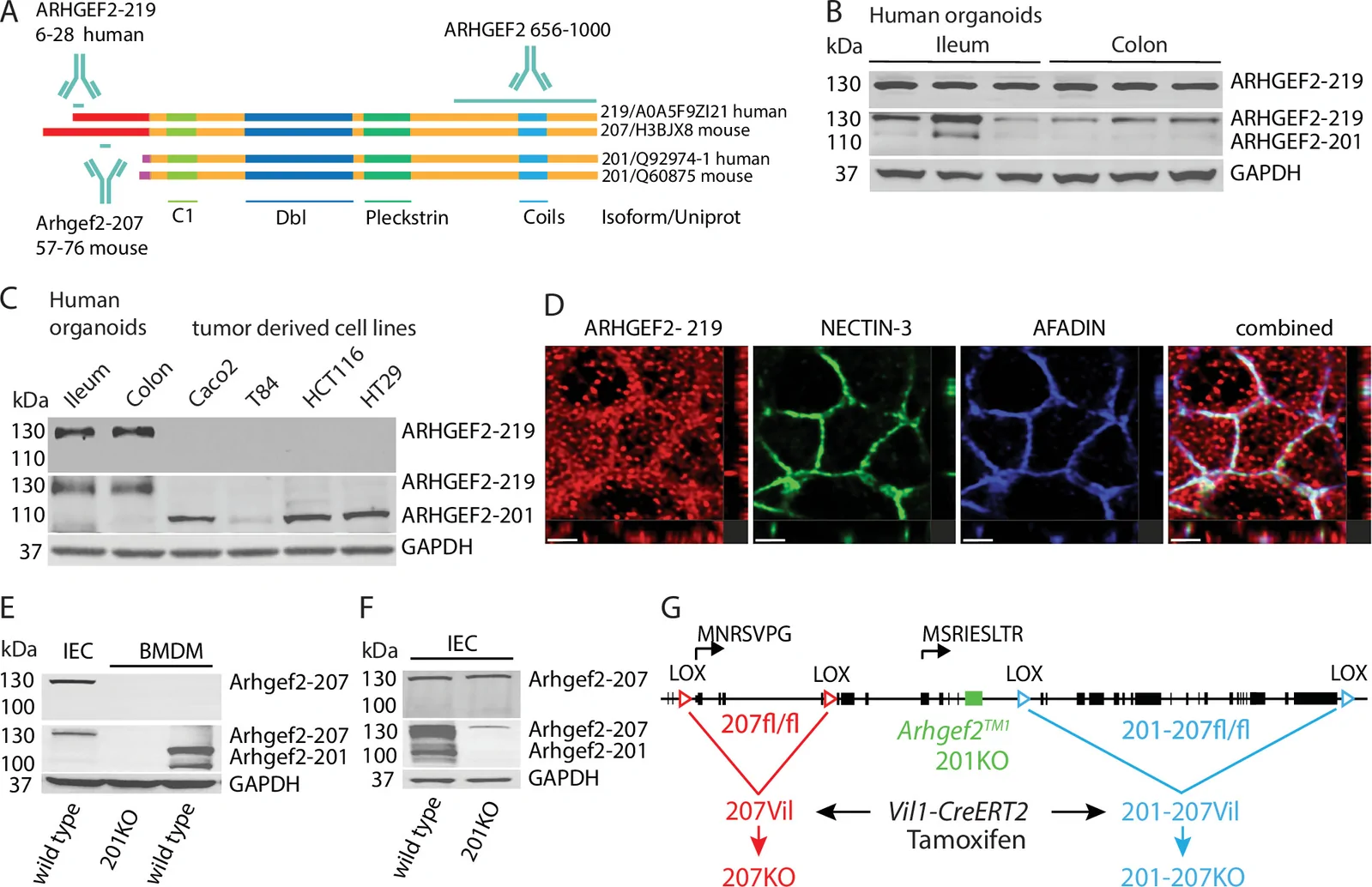
Human and mouse epithelial cells express long ARHGEF2 isoforms. **A** Domain alignment of human and mouse ARHGEF2 isoforms, highlighting the unique N-terminal leader sequences of the long (red) and short (lilac) variants. Antibody recognition regions used in this study are shown in muted teal, with corresponding amino acid positions indicated. Specific antibodies detecting AA6-28 of human ARHGEF2-219 and AA57-76 of mouse Arhgef2-207 were used to detect the long isoforms. An antibody raised against AA656-1000 of human ARHGEF2 was used to detect human and mouse long and short isoforms. **B** Immunoblot analysis of 3 independent derived ileal and colonic organoids, confirming expression of the long ARHGEF2-219 isoform detected by the isoform-specific and common antibody. Experiments were replicated two times with consistent results. **C** Western blot analysis comparing ARHGEF2 expression in human intestinal organoids and colon cancer–derived cell lines. Experiments were independently repeated three times and yielded consistent results. **D** Confocal microscopy of formalin-fixed, paraffin-embedded human colonic epithelium showing ARHGEF2-219 localization at NECTIN–AFADIN–junctions. Scale bars, 2 μm. **E** Immunoblot analysis of ARHGEF2 isoform expression in mouse intestinal organoids and BMDMs from wild-type or Arhgef2^TM1^ (201KO) mice. Experiments were independently repeated three times, with two samples per mouse strain in each experiment, and yielded consistent results. **F** Immunoblot analysis of ARHGEF2 isoforms in organoids derived from wild-type or 201KO mice. Experiments were independently repeated three times, with two samples per mouse strain in each experiment, and yielded consistent results. **G** Targeting strategy for isoform-specific and pan-isoform deletion of *Arhgef2* in intestinal epithelial cells. The positions of loxP sites enabling selective deletion of Arhgef2-207 (red) or deletion of all Arhgef2 isoforms (blue) are shown, together with the genetrap insertion site in Arhgef2TM1 mice (green). Transcriptional start sites and isoform-specific N-terminal amino-acid sequences for Arhgef2-207 and Arhgef2-201 are indicated by arrows. Crossing these alleles to Vil1-CreERT2 mice generated 207Vil and 201-207Vil lines, which produced 207KO and 201-207KO intestinal epithelial deletion models following tamoxifen treatment. Source data for all experiments are provided as Source Data files.

We generated isoform-specific antibodies to distinguish human ARHGEF2-219 and mouse Arhgef2-207, which contain unique N termini preceding the shared Dbl and pleckstrin homology domains shared with the shorter ARHGEF2-201 isoforms (Fig. 1A). Human small and large intestinal organoids expressed the long ARHGEF2-219 isoform (Fig. 1B), which was also detected using antibodies against the common C terminus, confirming that ARHGEF2-219 and ARHGEF2-201 share downstream domains (Fig. 1B). Notably, ARHGEF2-219 was absent from colorectal adenocarcinoma cell lines (Caco-2, T-84, HCT116 and HT-29), which predominantly expressed the shorter ARHGEF2-201 isoform (Fig. 1C). ARHGEF2-219 localized in the region of Nectin-3 and Afadin at adherens junctions (AJ) in human colonic epithelium (Fig. 1D).

Arhgef2-207, the mouse orthologue of ARHGEF2-219, was the predominant isoform in mouse small-intestinal IECs, as shown by immunoblotting and mass spectrometry (Fig. 1E and Supplementary Data 1). In contrast, bone marrow–derived macrophages (BMDMs) expressed only shorter Arhgef2 isoforms (Fig. 1E and Supplementary Data 2). Antibodies raised against AA 656–1000 of Arhgef2-201 also detected Arhgef2-207, confirming a shared C terminus (Fig. 1E). To further assess cell–type–specific isoform expression, we analyzed Arhgef2^TM1^ (201KO) mice carrying a gene-trap insertion downstream of exons encoding the Arhgef2-201–specific N terminus (IST13976A8)^15^ (Fig. 1G). These mice retained Arhgef2-207 expression in intestinal epithelial cells (IECs) but lacked shorter Arhgef2 isoforms in both IECs and macrophages (Fig. 1E, F). To define isoform-specific function, we generated conditional knockout mice targeting either the Arhgef2-207-specific N terminus (207fl/fl) or the C terminus shared by all isoforms (201-207fl/fl) by flanking isoform-specific or common exons with loxP sites (Fig. 1G). These mouse lines were crossed with Vil1-CreERT2 mice to generate 207Vil and 201-207Vil strains, enabling tamoxifen (TAM)-inducible, intestinal epithelial cell (IEC)-specific deletion of the targeted alleles. Throughout this study, these mice are referred to as 207KO and 201-207KO, respectively (Fig. 1G). For clarity, we designated mouse isoforms as Arhgef2-201 and Arhgef2-207 and human isoforms ARHGEF2-201 and ARHGEF2-219 throughout the manuscript.

Collectively, these findings show that differentiated human and mouse intestinal epithelial cells express ARHGEF2 isoforms with unique N termini, which are replaced by shorter variants in intestinal cancer cell lines.

### Loss of epithelial Arhgef2-207 compromises barrier integrity and causes small-intestinal inflammation

**Fig. 2:**
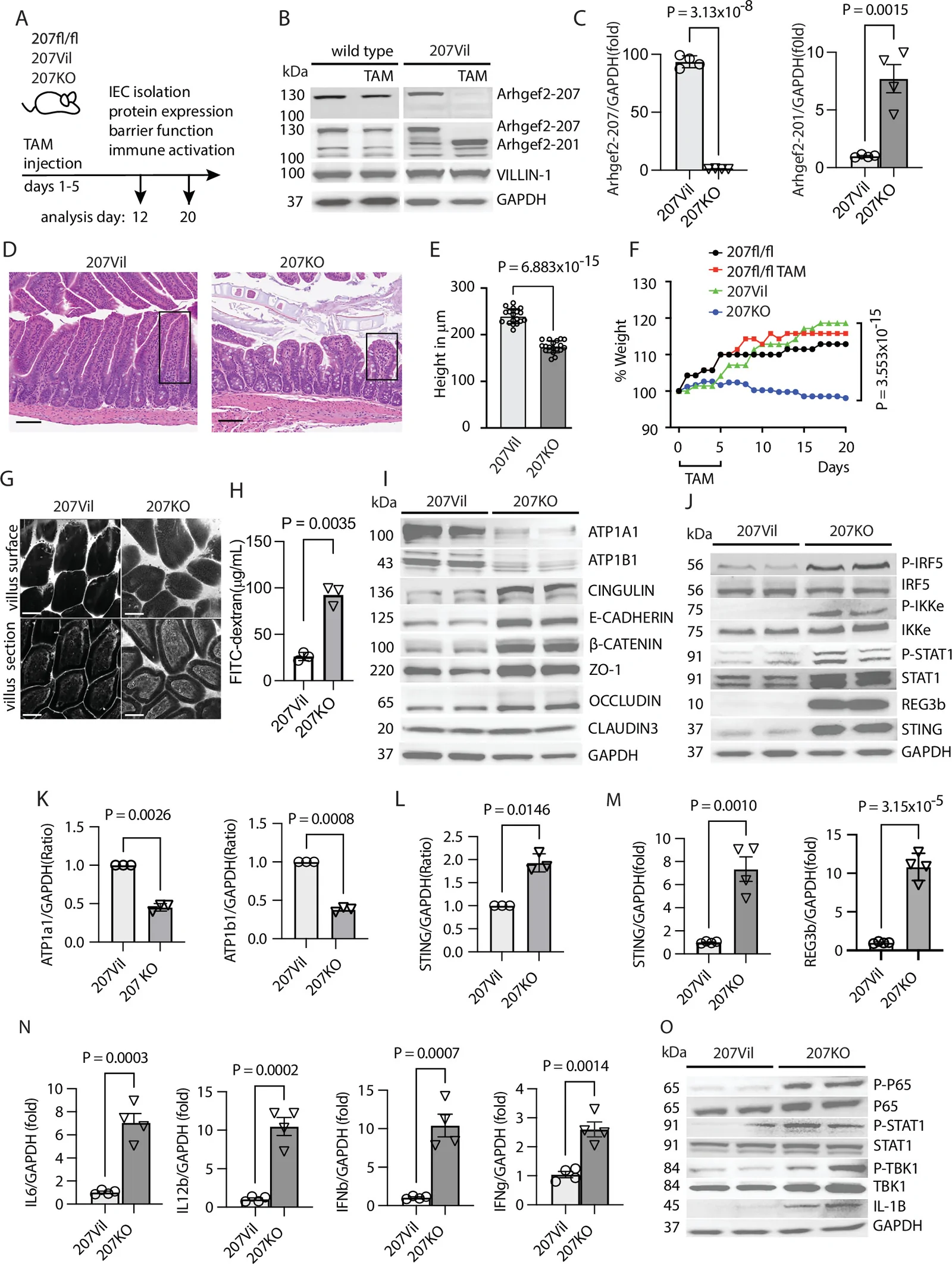
Inducible deletion of ARHGEF2-207 disrupts epithelial barrier integrity and elicits small-intestinal inflammation. **A** An experimental design. Mice received tamoxifen (TAM) for 5 days; small-intestinal IECs were analyzed 7 and 15 days after induction. **B** ARHGEF2 immunoblotting in IECs from wild-type and 207Vil mice before and after deletion. Three independent experiments, each using two samples per genotype, gave consistent results. **C** qPCR of *Arhgef2-207* and *Arhgef2-201* before (gray) and after deletion (black); *n* = 4 per group. Two-sided unpaired Student’s *t*-test. **D** Representative H&E-stained small-intestinal sections 7 days after induction. Boxes indicate villus-height measurements above crypts; *n* = 5 per genotype and time point. Scale bar, 50 µm. **E** Ileal villus height before (gray) and after induction (dark gray); *n* = 20 per condition. Two-sided unpaired Student’s *t*-test. **F** Body-weight changes following TAM treatment; *n* = 5–7 per strain. Two-way repeated-measures ANOVA with genotype/treatment and time as factors, followed by Šídák’s or Tukey’s multiple-comparisons test. Adjusted *P* values are shown. **G** Confocal images of ileal villi 1 h after oral FITC–dextran administration. Scale bar, 100 µm. **H** Serum FITC–dextran 4 h after gavage with 200 µL of 80 mg/mL FITC–dextran; *n* = 3 per condition. Two-sided unpaired Student’s *t*-test. **I** Immunoblotting of junctional proteins in IECs before and after induction. Two independent experiments, each using two samples per strain, gave consistent results. **J** Immunoblotting of inflammatory transcription factors, innate immune kinases, REG3β, and STING in ileal IECs. Two independent experiments, each using two samples per strain, gave consistent results. **K**,** L** ImageJ densitometry of ATP1A1, ATP1B1, and STING immunoblots; *n* = 3 per group. Two-sided unpaired Student’s *t*-test. **M** qPCR of *Sting* and *Reg3b* in small-intestinal lamina propria before (light gray) and after induction (dark gray); *n* = 4 per group. Two-sided unpaired Student’s *t*-test. **N** qPCR of transcription factors and immune regulators in mesenteric lymph nodes; *n* = 4 per group. Two-sided unpaired Student’s *t*-test. **O** Immunoblotting of immune regulators and inflammatory factors in mesenteric lymph nodes. Two independent experiments gave consistent results. Quantitative data are mean ± SEM, with *P* values indicated. Source data are provided as Source Data files.

We analyzed small intestines and isolated IECs from 207fl/fl, 207Vil and 207KO mice between 7 and 20 days post-induction of Cre expression (Fig. 2A). Deletion of Arhgef2-207 in IECs led to compensatory upregulation of Arhgef2-201, while TAM treatment had no effect on Arhgef2 expression in wild-type controls (Fig. 2B). qPCR analysis confirmed increased Arhgef2-201 transcript levels and lack of Arhgef2-207 mRNA expression in IECs from 207KO mice (Fig. 2C).

Histological analysis of 207KO mice revealed marked villus atrophy, epithelial disorganization, and a significant reduction in villus height 12 days post-TAM (Fig. 2D, E). Unlike control mice, 207KO mice failed to gain weight during the 20-day observation period (Fig. 2F).

To assess epithelial barrier function, we administered FITC–dextran (40 kDa) orally and analyzed its localization by confocal microscopy four hours later. In 207KO mice, FITC–dextran penetrated intercellular spaces and lamina propria of the small intestine, whereas 207Vil controls exhibited intact epithelial architecture with minimal FITC signal beyond the apical surface (Fig. 2G). Quantification of serum FITC–dextran four hours after oral gavage demonstrated a significant increase in systemic uptake in 207KO mice, consistent with compromised intestinal barrier function (Fig. 2H).

Deletion of Arhgef2-207 caused the loss of the Na^+^/K^+^-ATPase subunits ATP1A1 and ATP1B1 (Fig. 2I) that are required for the development of epithelial polarity^18^. Quantification across samples from independent experiments revealed a concomitant reduction in ATP1A1/B1 expression (Fig. 2K). Western blot revealed elevated levels of CINGULIN, E-CADHERIN, β-CATENIN, ZO-1, and OCCLUDIN in 207KO IECs, while expression of CLAUDIN-3 remained similar in 207KO compared to control IECs (Fig. 2I).

At the molecular level, loss of Arhgef2-207 was associated with activation of host-defense signaling in IECs, evidenced by increased phosphorylation of IRF5, IKKε and STAT1, together with elevated STING and REG3b protein abundance relative to IECs from 207fl/fl controls (Fig. 2J, L). Induction of STING and REG3b was further validated at the transcript level in IECs isolated from 207KO and control mice (Fig. 2M).

The lamina propria of 207KO mice exhibited increased expression of *Ifnb1, Il12b, Il6*, and *Ifng* transcripts, consistent with mucosal inflammation (Fig. 2N). Immune activation extended to mesenteric lymph nodes, where phosphorylation of NF-κB, p65, STAT1, and TBK1 was elevated, along with increased IL-1β expression (Fig. 2O). Loss of epithelial barrier integrity markedly increased the susceptibility of 207KO mice to DSS-induced colitis compared with 207fl/fl controls (Fig. S1A). Following a five-day course of 3% DSS in drinking water, 207KO mice exhibited a significantly impaired recovery response relative to controls. During the recovery phase, mesenteric lymph nodes from 207KO mice showed elevated expression of pro-inflammatory cytokines, including IL-6 and IL-1b, consistent with sustained inflammatory activation (Fig. S1B).

Together, these findings show that Arhgef2-207 is required for intestinal epithelial barrier integrity. Its ablation induced compensatory upregulation of Arhgef2-201, which failed to restore barrier function and resulted in spontaneous small-intestinal inflammation and increased susceptibility to DSS colitis. These data establish a nonredundant role for Arhgef2-207 in maintaining epithelial barrier function and intestinal homeostasis.

### Arhgef2-207 and Arhgef2-201 engage distinct junctional networks in polarized epithelia

**Fig. 3:**
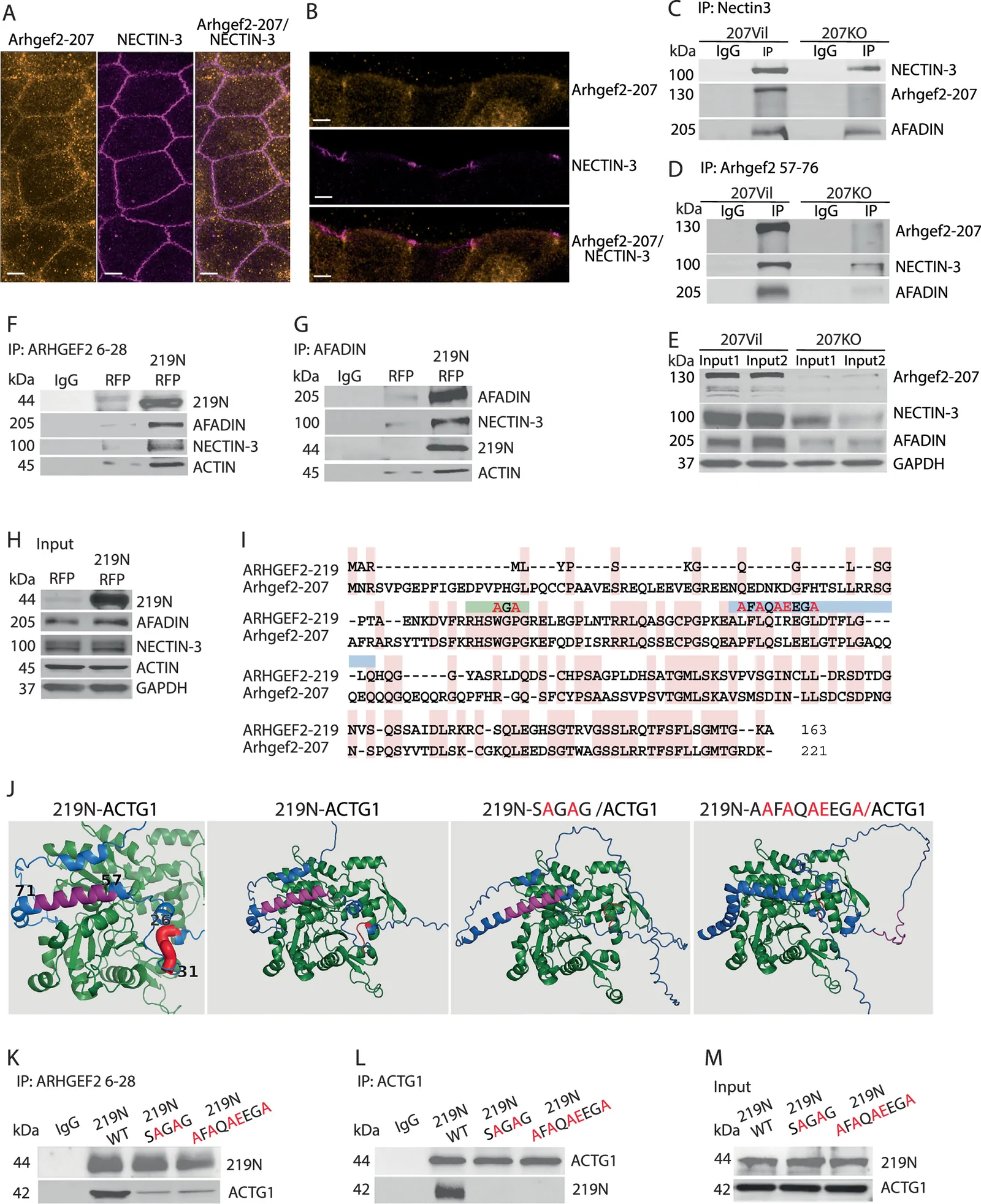
Isoform-specific coupling of Arhgef2 isoforms to epithelial junctional complexes. **A** STED image of mouse small-intestinal epithelium stained with the indicated antibodies. Scale bars, 2 µm. **B** Cross-sectional STED images showing colocalization of Arhgef2-207 and NECTIN-3 at apical junctional complexes. Scale bars, 1 µm. **C**,** D** Immunoprecipitation of NECTIN-3 (**C**) or Arhgef2-207 (**D**) from isolated small-intestinal IECs before and after Cre induction. **E** Immunoblotting of lysates corresponding to **C** and **D**. **F** Immunoprecipitation with an ARHGEF2-219-specific antibody (AA 6–28) in ARPE-19 cells expressing RFP or the RFP-tagged ARHGEF2-219 N terminus (219 N). The same antibody was used to detect 219 N. **G** Immunoprecipitation of AFADIN-associated complexes from ARPE-19 cells expressing RFP or 219 N. **H** Immunoblotting of lysates corresponding to **F** and **G**. **I** Alignment of the human ARHGEF2-219 and mouse Arhgef2-207 N termini. Conserved residues are pink; the RHSWGPG sequence and AA 55–71 motif within the predicted actin-interaction region are green and blue, respectively. Mutated residues are red; dashes indicate alignment gaps. **J** AlphaFold-Multimer models of 219N–ACTG1 complexes. In the WT model, the 219 N helix ALFLQIREGLDTFLGLQ forms a compact ACTG1 interface defined by ≤4 Å contacts; AA 26–31 and 57–71 are shown in red and purple, respectively. In the SAGAG and AAFAQAEEGADTFLGLQ mutants, this helix is disrupted and displaced from ACTG1, eliminating the contiguous interface without altering the overall ACTG1 fold. Models were aligned on ACTG1 and visualized in PyMOL under identical conditions. **K** Immunoprecipitation of lentivirally expressed 219 N from ARPE-19 cells using the ARHGEF2 AA 6–28 antibody. **L** Immunoprecipitation of ACTG1-associated complexes from ARPE-19 cells. **M** Immunoblotting of lysates corresponding to K and L. All experiments were repeated independently at least twice and yielded consistent results. Source data are provided as Source Data files.

STED imaging demonstrated that Arhgef2-207 co-localized with NECTIN-3 at the level of AJs in wild-type IECs (Fig. 3A, B). The AJ consists of two concentric rings: an apical ring containing NECTIN-AFADIN complexes and a basal ring composed of E-CADHERIN-CATENIN assemblies^19^. Co-immunoprecipitation experiments from primary IECs confirmed complexes that contained Arhgef2-207, NECTIN-3 and AFADIN, detectable in 207Vil but absent in 207KO IECs (Fig. 3C–E). Arhgef2-207 localized beneath the tight junction marker ZO-1 (Fig. S2A). Quantitative analysis revealed that Arhgef2-207 was positioned 340.0 ± 18.7 nm basally relative to the ZO-1 signal, consistent with localization to the AJ (Fig. S2B). Additionally, Arhgef2-207 was distributed in punctae across the apical cytoplasm, reflecting possible association with the apical F-actin and microtubule networks (Figs. 3A, B and S2A, B). Staining specificity was confirmed by the absence of signal in 207KO IECs using antibodies raised against amino acids 57–76 of the 207 isoform-specific N terminus (Fig. S2A).

In contrast, the short Arhgef2-201 isoform, detected using antibodies against a shared C-terminal epitope, enriched in a punctate distribution at the level of ZO-1, situated 96.5 ± 6.5 nm horizontally from the tight junction marker in both 207Vil and 207KO mice (Fig. S2C, D). Upon deletion of Arhgef2-207, Arhgef2-201 remained localized to TJs (Fig. S2C). Immunoprecipitation with antibodies directed against ZO1 pulled down the short Arhgef2-201 isoforms but not the long ARHGEF2-207 isoform (Fig. S2E, F).

To examine whether the specific N-terminal region of the long ARHGEF2 isoform mediates junctional or cytoskeletal associations, RFP fusion constructs encoding the N terminus of ARHGEF2-219 (219 N) were expressed in the non-transformed human epithelial cell line Arpe-19. Immunoprecipitation using antibodies directed against amino acids 6–28 of ARHGEF2-219 identified AFADIN, NECTIN-3 and ACTIN as potential interactors (Fig. 3F, H). Reciprocal immunoprecipitation for AFADIN confirmed the presence of complexes containing the ARHGEF2-219 N-terminal domain together with NECTIN-3 and ACTIN (Fig. 3G, H).

We analyzed the N terminal sequence 219 N for potential F-actin binding domains as Afadin functions as an actin binding junctional scaffold^20–22^. The N-terminal regions of ARHGEF2-219 and Arhgef2-207 display moderate overall similarity, with 43.2% identity across the full global alignment and 70.7% similarity when conservative substitutions are included, and conservation concentrated in discrete motifs including RHSWGPG, TGMLSK and GSSLR-TFSFL-GMTG. (Fig. 3I).

AlphaFold multimer modeling of a 219N-ACTG1 complex generated a high-confidence assembly (ipTM ≈ 0.79; ranking score ≈ 0.93) in which 219 N engages ACTG1 through a composite interface formed by two spatially coordinated regions (Supplementary Data 3). The first region corresponds to a conserved short N-terminal motif, RHSWGP (residues 26–31) (Fig. 3I, J). Within this segment, residues R26, H27, S28, W29, G30, and P31 form direct inter-chain atom–atom contacts (≤4 Å) in the top-ranked model (Fig. 3J and Supplementary Data 3). A second interface segment spans residues 51–71 (EALFLQIREGLDTFLGLQHQG), where residues F57, I60, R61, L64, D65, F67, L68, G69, and Q71 also satisfy the ≤4 Å contact criterion (Fig. 3I, J and Supplementary Data 3). Together, these two regions form a contiguous ACTG1-facing surface in the predicted wild-type assembly (Fig. 3J).

To test the contribution of each segment, two mutants were modeled. Mutations in motif residues 26–31 (W29A/P31A) revealed that the W29A/P31A mutation disrupts the N-terminal 26–31 interface motif and remodeled the broader interaction surface (Fig. 3J and Supplementary Data 4). In the wild-type model, residues 26-30 form a compact contact patch with ACTG1 residues 211–215 and 56. In contrast, the mutant abolishes all <4.0 Å contacts for residues 28–31 and substantially reduces contacts for residues 26 and 27, accompanied by a shift in contacting ACTG1 residues (Fig. 3J and Supplementary Data 4).

Notably, the mutation also alters contacts within the distal 55–71 region. Although this segment remained engaged with ACTG1, several contacts are lost or redistributed, including loss of interactions involving residues 57, 60, 61 and 69 (Supplementary Data 4). Together, these findings indicate that perturbation of the 26–31 motif induces broader interface remodeling in the predicted complex (Fig. 3J and Supplementary Data 4).

Replacement of residues 55–71 with AAFAQAEEGADTFLGLQ resulted in a marked reduction in predicted inter-chain confidence (ipTM ≈ 0.33; ranking score ≈ 0.55), accompanied by loss of direct contacts within the 55–71 region (Fig. 3J and Supplementary Data 5). The mutant model failed to maintain a stable relative orientation between 219 N and ACTG1, consistent with disruption of the composite interface. Mutating this region also remodeled interaction in the upstream 26-31 motif. While residue G30 lost its <4.0 Å contact in the mutant model, H27 and W29 retained contacts, but these contacts shift to different ACTG1 surface regions relative to the wild-type configuration. For example, W29 transitions from engaging ACTG1 residues 30 and 56 to a distinct cluster encompassing residues 214, 302, 305, 306, and 336 (Fig. 3J and Supplementary Data 5).

Immunoprecipitation assays confirmed that both mutants failed to associate with ACTG1 when expressed in Arpe-19 cells (Fig. 3K–M).

These findings reveal isoform-specific compartmentalization of ARHGEF2 in polarized epithelial cells. The long ARHGEF2-219 isoform gains actin-binding capacity through its unique N terminus and localizes to adherens junctions (AJs), where it associates with NECTIN–AFADIN complexes, while retaining the microtubule-binding domains present in ARHGEF2-201. ARHGEF2-219 engages ACTG1 through a bipartite interface within this isoform-specific N-terminal region that requires cooperative contributions from both the RHSWGP motif and residues 51–71, with the disruption of either element abolishing actin binding.

### Loss of Arhgef2-207 triggers autophagic responses in the intestinal epithelium

**Fig. 4:**
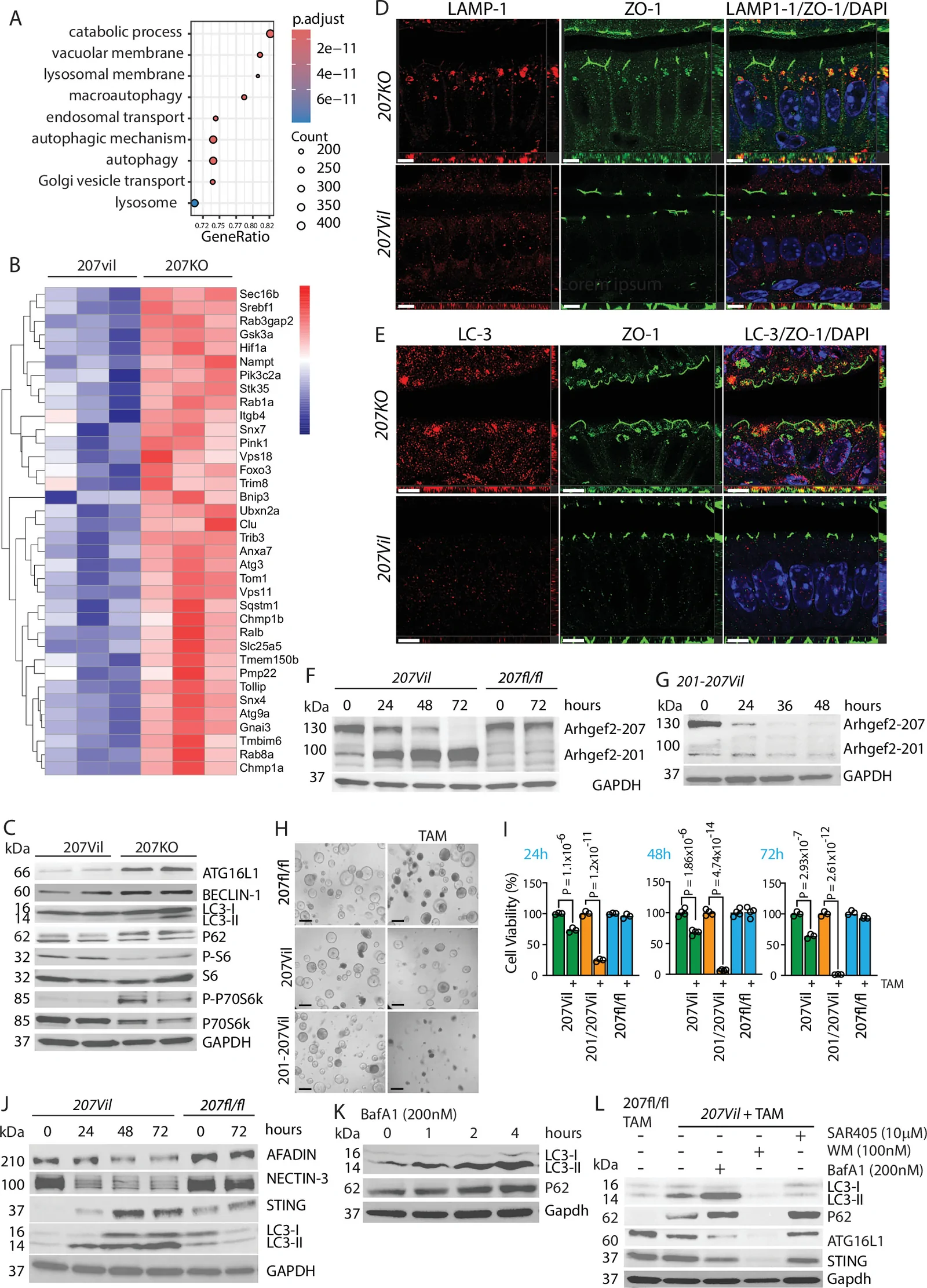
Loss of epithelial Arhgef2-207 drives an autophagy-dominated stress program. **A** Gene Ontology enrichment analysis of genes differentially expressed in IECs after small-intestinal *Arhgef2-207* deletion. The nine most enriched pathways are shown. Enrichment was assessed by a one-sided hypergeometric test with Benjamini–Hochberg false-discovery-rate correction. Dot size represents gene count, color represents adjusted *P* value, and GeneRatio represents the proportion of input genes assigned to each term. **B** Hierarchical clustering of autophagy-related genes upregulated after *Arhgef2-207* deletion; three independent biological replicates. **C** Immunoblotting of autophagy-associated proteins in IECs before and after Cre induction. Two samples per condition are shown; two independent experiments gave consistent results. **D**,** E** Confocal imaging of ZO-1, LAMP-1, and LC3 in small-intestinal epithelium before and after *Arhgef2-207* deletion. Scale bars, 5 µm. Two independent experiments, each using two samples per strain, gave consistent results. **F**,** G** ARHGEF2 isoform expression during TAM treatment of 207Vil and 207fl/fl organoids (**F**) or 201/207Vil organoids (**G**). Two independent experiments gave consistent results. **H** Representative organoids of the indicated genotypes before and after TAM treatment. Scale bars, 200 µm. Three independent experiments gave consistent results. **I** Organoid viability before and after TAM treatment in 207Vil (green), 201/207Vil (orange), and 207fl/fl (blue) organoids; *n* = 3 per group. Data are mean ± SEM; indicated *P* values were determined by one-way ANOVA. **J** Immunoblotting of junctional and autophagy-associated proteins in 207Vil and 207fl/fl organoids at the indicated times after TAM treatment. **K** LC3 and p62 immunoblotting in 207Vil and 207fl/fl organoids treated with 10 µM TAM for 48 h, followed by 200 nM bafilomycin A1 (BafA1) for 0–4 h. **L** LC3, p62, ATG16L1, and STING immunoblotting in TAM-treated 207Vil and 207fl/fl organoids after 4 h of treatment with 200 nM BafA1, 100 nM wortmannin (WM), or 10 µM SAR405. Experiments in **F**, **G**, **J**–**L** were repeated independently twice and yielded consistent results. Source data are provided as Source Data files.

To delineate the transcriptional consequences of Arhgef2-207 loss and Arhgef2-201 induction, we performed RNA-sequencing (RNA-seq) on IECs isolated from 207Vil and 207KO mice. Differential gene expression analysis identified 1,175 genes significantly regulated in 207KO compared to 207Vil IECs (Supplementary Data 6). Gene ontology enrichment revealed a marked upregulation of pathways associated with proteasomal degradation, lysosomal membrane organization, and autophagy (Fig. 4A). In this enrichment, 207KO IECs exhibited increased expression of genes associated in gene ontology data sets with GO:0006914 - autophagy, GO:0016236- macroautophagy, and GO:0010506—regulation of autophagy) including Atg3^23^, Atg9a^24^, Sqstm1/p62^25^, Tollip^26^, Trib3^27^, Chmp1a/Chmp1b^28^. This includes genes that have been shown to regulate autophagy, such as Foxo3^29^ and Hif-1a^30^ (Fig. 4B).

Immunoblots confirmed the upregulation of autophagy proteins ATG16L1, BECLIN-1, LC3-II and P62 in targeted IECs (Fig. 4C). Phosphorylation of Ribosomal protein S6 kinase beta-1, or S6K1 (P70S6k), was decreased, alongside increased phosphorylation of S6 kinase (Fig. 4C).

To assess whether epithelial responses extended to cell death pathways, we profiled markers of apoptosis, necroptosis, and pyroptosis. Surprisingly, 207KO IECs exhibited reduced expression of cleaved Caspase-3, Caspase-8, and Caspase-1, as well as necroptotic effectors MLKL and RIPK3 (Fig. S3). In contrast, we observed an increased expression of cleaved Gasdermin D (GSDMD) (Fig. S3) that may be involved in the exocytosis of mucin granules by goblet cells and nonlytic IL-1β release in IECs^31,32^.

We next examined whether Arhgef2-207 deficiency promoted the formation of autophagy-related structures in IECs. IECs from 207KO mice formed intracellular compartments enriched for the tight junction protein ZO-1, which were absent in 207Vil controls (Fig. 4D, E). These compartments contained LAMP-1 and LC3 (Fig. 4D, E).

To determine whether autophagic responses were a direct epithelial response to Arhgef2-207 loss and induction of Arhgef2-201 expression, we generated intestinal organoids from 207fl/fl, and 207Vil, and 201-207Vil mice (Fig. 4F). In 207Vil organoids, TAM-induced Cre activation led to progressive depletion of ARHGEF2-207 (130 kDa) over 72 h, accompanied by upregulation of the Arhgef2-201 isoform (110 kDa), replicating the in vivo switch observed in primary 207KO IECs (Fig. 4F). Tamoxifen treatment had no effect on Arhgef2 isoform expression in 207fl/fl controls (Fig. 4F). In contrast, 201-207Vil organoids, which delete all Arhgef2 isoforms, showed complete loss of Arhgef2 expression within 48 h of TAM treatment (Fig. 4G).

Functionally, Arhgef2-207 deficiency reduced organoid viability by 20–35% (Fig. 4H, I), whereas complete loss of all Arhgef2 isoforms resulted in rapid and profound epithelial collapse (Fig. 4H, I). Treatment of 207fl/fl organoids with TAM had no impact on cell viability (Fig. 4H, I). Thus, Arhgef2 expression is required for epithelial organoid survival, but Arhgef2-201 can compensate for Arhgef2-207 loss to sustain long-term epithelial viability.

Deletion of Arhgef2-207 resulted in the downregulation of AFADIN and NECTIN-3, induction of STING and LC3 lipidation (Fig. 4J). TAM treatment of 207fl/fl had no impact on junctional or autophagic protein expression (Fig. 4J). Importantly, Arhgef2-207 deletion in both small and large intestinal organoids induced autophagic gene expression, mirroring the phenotype observed in vivo. This included upregulation of LC3-II, ATG16L1, and Beclin-1, as well as downregulation of Na⁺/K⁺-ATPase subunits ATP1A1 and ATP1B1 (Fig. S4).

Compared with wild-type organoids, the Arhgef2-201-high state showed a clear increase in LC3-II and p62, consistent with activation of an autophagy-associated response (Fig. 4K). Treatment with bafilomycin A1 further increased LC3-II and p62, indicating ongoing LC3 turnover under basal conditions in the 201-high state (Fig. 4K, L). In contrast, inhibition of PI3K activity with wortmannin broadly reduced LC3-II, p62, ATG16L1 and STING, indicating that PI3K activity is required for maintenance of the Arhgef2-201-associated program (Fig.4L). By comparison, inhibition of VPS34, the class III PI3K, with SAR405 reduced LC3-II but caused marked accumulation of p62 and decreased STING, consistent with impaired autophagic degradation following VPS34 inhibition (Fig.4L).

Together, these findings indicate that loss of Arhgef2-207 reprograms IECs toward a stress response with the upregulation of Arhgef2-201 and induction of a PI3K- and VPS34-dependent autophagy program with the induction of STING.

### *L. monocytogenes* infection induces an ARHGEF2 isoform switch in human epithelium

The loss of epithelial barrier integrity following Arhgef2-207 deletion, together with the compensatory induction of ARHGEF2-201 and activation of autophagy and innate immune transcriptional programs, suggested that the long ARHGEF2 isoform plays a central protective role in epithelial host defense against invasive pathogens. To determine whether enteric pathogens exploit this regulatory mechanism during infection, we investigated the regulation of ARHGEF2 isoforms during infection with *L. monocytogenes*, which disrupt epithelial junctions during invasion and have evolved strategies to escape autophagic clearance^7,8,33,34^.

Because *L. monocytogenes* invasion is mediated via internalin A binding to human E-cadherin^33^, we employed human small intestinal organoids to model host-pathogen interactions.

**Fig. 5:**
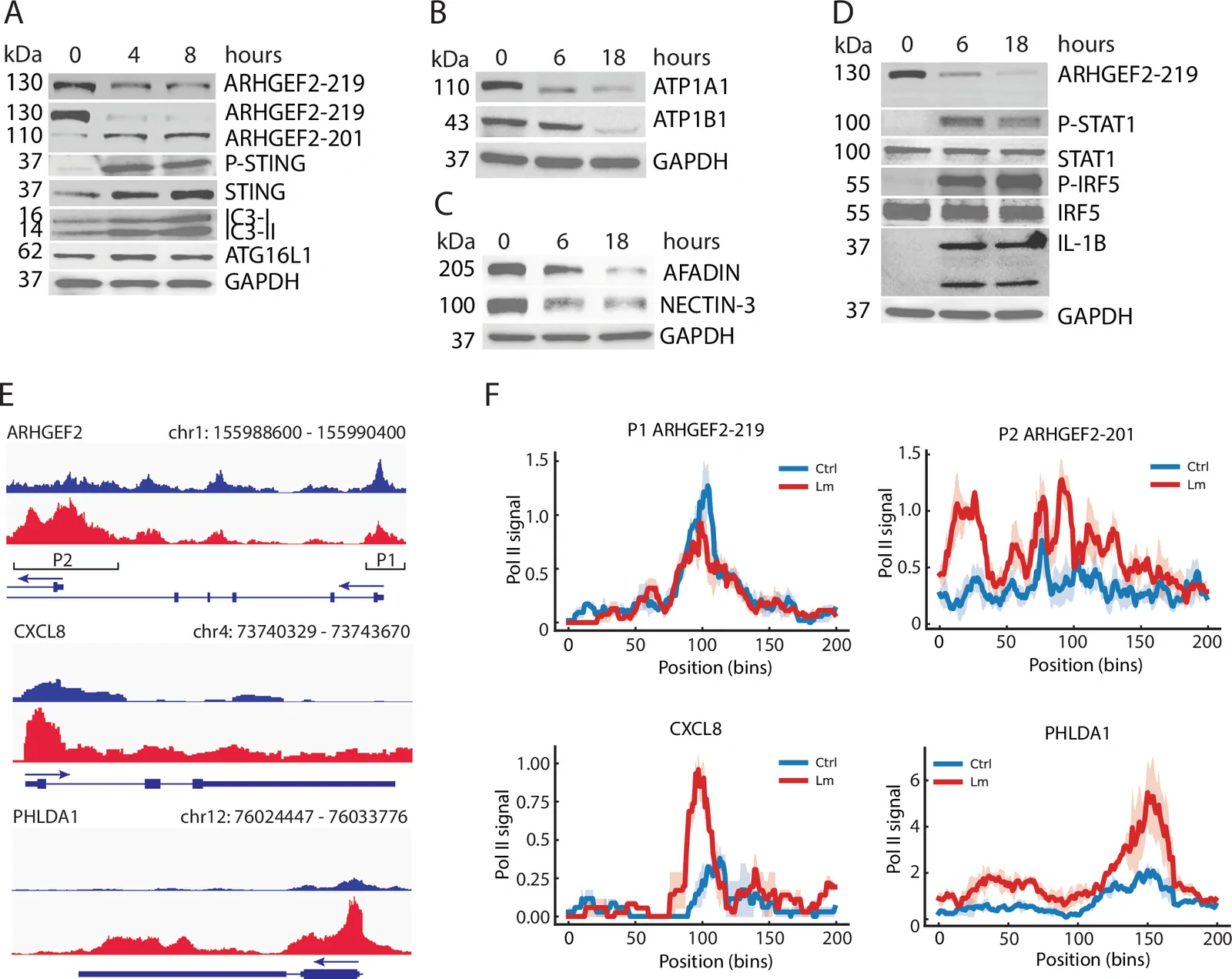
*L. monocytogenes* selectively targets the long ARHGEF2 isoform and induces expression of the short isoform. **A** Immunoblot analysis of ARHGEF2 isoforms and autophagy-related proteins (LC3, ATG16L1, STING) in intestinal organoids before and after with 5 × 10^5^ CFU of Listeria monocytogenes per well (100 organoids per well in a 24-well plate) to assess regulation of ARHGEF2 isoform expression. Experiments were independently repeated two times and yielded consistent results. **B** Immunoblot analysis of Na⁺/K⁺-ATPase subunits (ATP1A1, ATP1B1) in organoids before and after *L. monocytogenes* infection. Experiments were independently repeated two times and yielded consistent results. **C** Immunoblot analysis of AFADIN and NECTIN-3 expression in organoids before and after *L. monocytogenes* infection. Experiments were independently repeated three times and yielded consistent results. **D** Immunoblot analysis of innate immune signaling proteins in organoids before and after *L. monocytogenes* infection, including phosphorylated STAT1 and IRF5, and pro- and cleaved IL-1β. Experiments were independently repeated two times and yielded consistent results. **E** Genome browser tracks showing Pol II CUT&Tag profiles from control (blue) and *L. monocytogenes*–infected (red) human intestinal organoids for 4 h across the *ARHGEF2*, *CXCL8*, and *PHLDA1* loci (hg38). Two distinct ARHGEF2 promoters are evident: a proximal P1 promoter and an internal P2 promoter. Arrows indicate gene orientation, and exons are shown relative to the PolII profile. **F** Promoter-centered Pol II CUT&Tag profiles (mean ± s.e.m.) for ARHGEF2 P1 (219-isoform TSS), ARHGEF2 P2 (201-isoform TSS), CXCL8, and PHLDA1, generated from ±1 kb windows centered on the Pol II peak summit (200 bins). Curves represent the mean of three biological replicates per condition (Ctrl, blue; Lm, red). Source data for all experiments are provided as Source Data files.

Upon *L. monocytogenes* infection, ARHGEF2-219 protein levels declined sharply within 4 to 8 h (Fig. 5A). This loss was accompanied by the induction of the short 110 kDa isoform, ARHGEF2-201, indicating an isoform switch in response to bacterial invasion (Fig. 5A). Strikingly, *L. monocytogenes* invasion led to the increased expression of lipidated LC3, ATG16L1 and STING^35^ (Fig. 5A). *Listeria* infection further led to the loss of Na^+^/K^+^-ATPase subunits ATP1A1 and ATP1B1 (Fig. 5B), and loss of AFADIN and NECTIN-3 (Fig. 5C), suggesting a disruption of junctional integrity and polarity upon cell invasion.

In parallel, human intestinal epithelial organoids activated canonical ARHGEF2-dependent innate immune pathways^16,36^, including phosphorylation of STAT1 and IRF5, and upregulation of IL1B expression, with subsequent IL-1β cleavage (Fig. 5D). These responses mirror ARHGEF2-dependent immune activation previously characterized in macrophages^16,17^.

Pol II CUT&Tag profiling revealed *Listeria* infection-induced changes in ARHGEF2 promoter usage (Fig. 5E, F). At the proximal ARHGEF2-219 (P1) promoter, Pol II occupancy showed only a modest reduction following 4 h of *Listeria* infection (Ctrl: 0.289 ± 0.023, Lm: 0.245 ± 0.026 RPGC; fold-change 0.85, log_2_FC –0.24), and the difference did not reach statistical significance (Welch’s t-test, *P* = 0.28) (Fig. 5F, G and Supplementary Data 7). In contrast, the ARHGEF2-201 (P2) promoter exhibited a strong induction of Pol II occupancy after infection (Ctrl: 0.312 ± 0.055, Lm: 0.633 ± 0.050 RPGC; fold-change 2.03, log_2_ FC 1.02; Welch’s t-test, *P* = 0.0127) (Fig. 5E, F and Supplementary Data 7).

We also quantified Pol II CUT&Tag signal at the CXCL8 and PHLDA1 promoters to confirm correct Pol II detection at genes induced in control and *Listeria*-infected human intestinal organoids (Fig. 5E, F and Supplementary Data 7). At the CXCL8 promoter, Pol II occupancy increased from 0.21 ± 0.03 to 0.80 ± 0.13 RPGC upon infection, corresponding to an approximately 3.9-fold induction (*p* = 0.036) (Fig.5E, F and Supplementary Data 7). At the TSS of PHLDA1, Pol II signal rose from 1.19 ± 0.28 to 3.95 ± 0.75 RPGC, representing an approximately 3.3-fold increase (*P* = 0.053) (Fig.5E, F and Supplementary Data 7).

Collectively, these findings demonstrate that *L. monocytogenes* infection remodels ARHGEF2 isoform expression in human intestinal epithelial cells through selective loss of ARHGEF2-219 and transcriptional induction of ARHGEF2-201. This isoform switch is accompanied by disruption of epithelial junctional complexes and activation of innate immune signaling, identifying ARHGEF2 isoform remodeling as a previously unrecognized component of the epithelial response to bacterial invasion.

### ARHGEF2-201 couples STING signaling to VPS34-dependent autophagy through ROCK

**Fig. 6:**
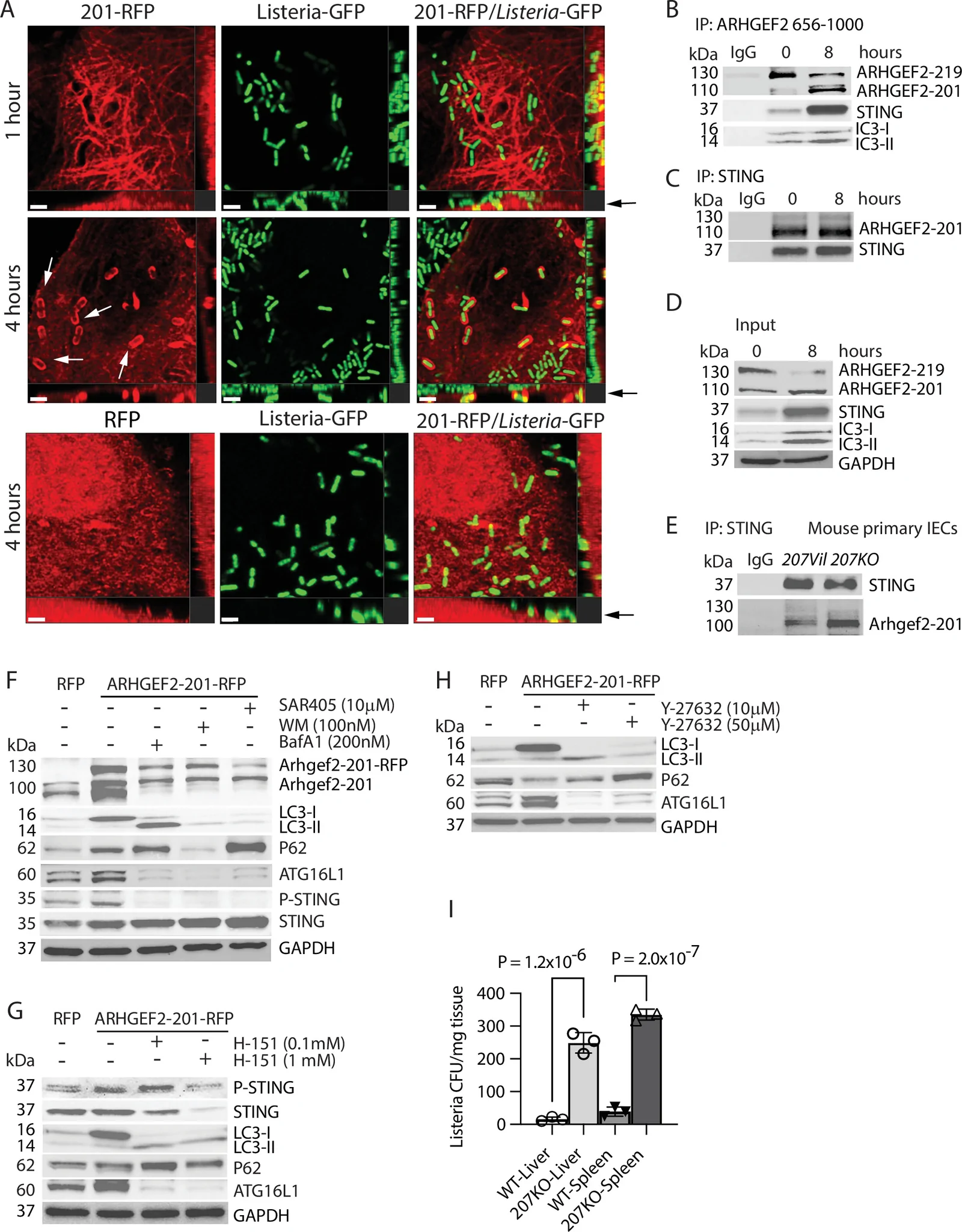
ARHGEF2-201 couples STING signaling to autophagy through RhoA-ROCK signaling. **A** Confocal microscopy of Caco-2 cells expressing RFP-ARHGEF2-201 or RFP following *L. monocytogenes* infection. White arrows indicate recruitment of ARHGEF2-201 to *Listeria*-containing endosomes. Black arrows denote the position of the z-section shown in the corresponding xy image. Scale bars, 2 µm. The experiment was independently repeated two times and yielded consistent results. **B–D** Immunoprecipitation of ARHGEF2 and STING from human colon organoids showing association of ARHGEF2-201 with STING and LC3 before and after *L. monocytogenes* infection. The experiment was independently repeated three times and yielded consistent results. **E** Immunoprecipitation of STING in IECs isolated from indicated strains of mice. The experiment was independently repeated two times and yielded consistent results. **F–H** Immunoblot of autophagy regulators in ARPE-19 cells expressing RFP or RFP-ARHGEF2-201 for 48 h and 4 h treatment with indicated inhibitors. Experiments were independently repeated two times and yielded consistent results. **I** Bacterial burden of *L. monocytogenes* in organs from WT and *207KO* mice 24 h after oral infection (1 × 10^8^ CFU). Organs were harvested, homogenized, and plated on LB–erythromycin agar for CFU enumeration. *n* = 3 per group. Data are presented as mean ± SEM with indicated *P* values analyzed by two-sided, unpaired Student’s *t*-test. Source data for all experiments are provided as Source Data files.

Following epithelial invasion, ARHGEF2-201 was recruited to intracellular *L. monocytogenes*. Caco-2 cells expressing ARHGEF2-201-RFP were infected with *L. monocytogenes* and analyzed by time-lapse 3D confocal microscopy using x-y-z optical stack acquisition to reconstruct cellular volumes. 1 h post-infection, intracellular bacteria were readily detected, while ARHGEF2-201 remained predominantly associated with microtubules (Fig. 6A). By 4 h post-infection, ARHGEF2-201 was redistributed to compartments surrounding intracellular bacteria (Fig. 6A). In contrast, RFP expressed in Caco-2 cells did not associate with intracellular *Listeria* (Fig. 6A).

Immunoprecipitation of ARHGEF2 from human organoids before and after *L. monocytogenes* infection revealed association with protein complexes containing STING and LC3, which increased following infection (Fig. 6B, D). Reciprocal immunoprecipitation of STING confirmed interaction with ARHGEF2-201, but not ARHGEF2-219 (Fig. 6C, D). This interaction was further validated in primary intestinal epithelial cells, where deletion of Arhgef2-207 increased association of Arhgef2-201 with STING-containing complexes (Fig. 6E).

Overexpression of ARHGEF2-201 increased total STING abundance and phosphorylation and was accompanied by elevated ATG16L1 and p62 levels relative to controls (Fig. 6F). LC3-I abundance was increased, whereas LC3-II was most evident following lysosomal inhibition with bafilomycin A1, indicating BafA1-sensitive LC3 turnover. Inhibition of VPS34 with SAR405 or broader PI3K inhibition with wortmannin reduced LC3-I and ATG16L1 levels, while SAR405 induced marked p62 accumulation and reduced STING phosphorylation, consistent with impaired autophagic degradation upon VPS34 inhibition (Fig. 6F).

Inhibition of STING with H-151 reduced phosphorylated STING levels in a dose-dependent manner and altered LC3 processing, with increased LC3-II and reduced LC3-I, accompanied by further p62 accumulation (Fig. 6G). These findings indicate that STING activity modulates LC3 processing and p62 levels in the ARHGEF2-201-induced response.

Given that ARHGEF2-201 functions as a RhoA guanine nucleotide exchange factor, we next examined the role of RhoA-ROCK signaling. Pharmacologic inhibition of ROCK with 50 μM Y-27632 resulted in a reduction in LC3-I and LC3-II, accompanied by marked p62 accumulation and decreased ATG16L1 levels (Fig. 6H), indicating that ROCK activity contributes to the ARHGEF2-201-associated autophagy response.

Finally, we assessed susceptibility to infection in vivo by orally infecting 207fl/fl and 207KO mice with 1 × 10^8^ *L. monocytogenes*. Loss of Arhgef2-207 significantly increased susceptibility, as evidenced by higher bacterial burdens in the livers and spleens 24 h after oral gavage (Fig. 6I).

Together, these findings identify ARHGEF2-201 as an infection-induced effector that is recruited to intracellular *L. monocytogenes*, associates with STING-containing complexes, and promotes STING-dependent autophagy signaling. Increased bacterial dissemination in 207KO mice further demonstrates that loss of the long ARHGEF2 isoform compromises host defense against invasive infection.

## Discussion

Our study identifies isoform-specific functions of ARHGEF2 that partition epithelial homeostasis and stress responses in the intestinal epithelium. We show that differentiated intestinal epithelial cells selectively express long ARHGEF2 isoforms (mouse Arhgef2-207 and human ARHGEF2-219), which localize to AJs through a unique N-terminal interface that engages actin and junctional scaffolds. In contrast, intestinal cancer cell lines and macrophages predominantly express shorter isoforms, indicating cell type- and differentiation-dependent promoter usage.

Functionally, Arhgef2-207 is required for epithelial organization and barrier integrity. Its deletion leads to villus atrophy, loss of polarity associated with Na^+^/K^+^-ATPase subunits, and increased paracellular permeability, demonstrating a nonredundant structural role of the long isoform in maintaining epithelial architecture. Despite compensatory upregulation of Arhgef2-201, barrier function is not restored, indicating that the short isoform cannot substitute for junctional anchoring mediated by the long isoform.

Mechanistically, the long ARHGEF2 isoforms are compartmentalized at AJs through interactions with NECTIN–AFADIN complexes and F-actin. Structural modeling and mutational analyses support a bipartite actin-binding interface within the isoform-specific N terminus, with residues 26–31 and 51–71 acting cooperatively to mediate stable ACTG1 binding. Disruption of either region abolishes this interaction, indicating that actin anchoring requires a composite interface rather than a single linear motif. The unique N terminus, therefore, expands the cytoskeletal-binding capacity of ARHGEF2 to include F-actin while preserving the microtubule-binding domains shared with the shorter ARHGEF2-201 isoform.

Loss of Arhgef2-207 destabilizes junctions, reduces Na⁺/K⁺-ATPase expression, and drives the accumulation of tight-junction proteins within LC3- and LAMP1-positive autophagosomes. These features parallel the enhanced autophagy observed after pharmacological Na^+^/K^+^-ATPase inhibition and reveal how junctional perturbation can directly engage the autophagic machinery^37,38^.

Loss of Arhgef2-207 reprograms epithelial cells toward a stress-associated state characterized by induction of autophagy-related pathways and innate immune signaling. IECs lacking Arhgef2-207 exhibit increased LC3 lipidation, accumulation of autophagy regulators, and activation of STING, IRF5, and STAT1 signaling. This response is accompanied by the formation of intracellular LC3- and LAMP1-positive compartments containing junctional proteins, suggesting redistribution of membrane components into autophagy-associated structures rather than classical apoptotic cell death.

The short ARHGEF2-201 isoform emerges as a key mediator of this response. Unlike the long isoform, ARHGEF2-201 localizes to tight junction compartments^10,39^ and interacts with STING-containing complexes, coupling innate immune activation to a VPS34- and PI3K-dependent autophagy program^35^. Pharmacological perturbation indicates that this program requires VPS34 activity and is modulated by ROCK signaling, linking ARHGEF2 GEF activity to membrane remodeling and LC3 conjugation.

Importantly, this isoform switch is recapitulated during infection with *L. monocytogenes*. Bacterial invasion reduces ARHGEF2-219 abundance while inducing ARHGEF2-201 expression, accompanied by activation of LC3 lipidation and STING signaling. Increased susceptibility of Arhgef2-207-deficient mice to infection supports a protective role for the long isoform at epithelial junctions, whereas the induced short isoform defines a secondary host response program.

Key questions remain regarding the transcriptional and post-translational mechanisms governing ARHGEF2 isoform expression and degradation, the structural determinants mediating autophagic targeting, and the upstream cues that trigger isoform switching. Determining whether *L. monocytogenes* internalins or other microbial factors directly destabilize ARHGEF2 junctional complexes will be essential to understanding how pathogens exploit this pathway.

From a translational perspective, ARHGEF2 isoform regulation may represent a critical determinant of epithelial differentiation. Primary IECs and organoids acquired expression of the long ARHGEF2 isoform upon differentiation, whereas human tumor-derived cell lines and organoids lacked its expression. Defining the role of this isoform in human cancer will be important, given prior links between ARHGEF2 and oncogenic signaling^40^.

Genome-wide association studies implicating autophagy genes, polarity factors, ARHGEF2 and its interactor NOD2 in IBD susceptibility^1–8,13,14,17,41^, suggest that defective maintenance or dysregulated switching of ARHGEF2 isoforms could shift the mucosa from adaptive repair to chronic inflammation.

Together, these findings establish a model in which ARHGEF2 isoforms perform nonredundant and sequential functions: the long isoform maintains junctional integrity through cytoskeletal anchoring to AJs, whereas the short isoform mediates stress-induced autophagic responses and innate immune signaling when epithelial integrity is compromised (Fig. S5). This provides a mechanism by which epithelial cells couple structural organization to immune activation through isoform-specific regulation of a single gene.

## Methods

Please see the key resources list for antibodies, primers and chemicals used. (Supplementary Data 8).

### Mice

All experimental mice were sex-matched at 7–12 weeks of age using protocols approved by the Committee on Research Animal Care at the UT Southwestern Medical Center, Dallas, under the protocol number 2020-102946 and performed according to the institutional guidelines. Up to 5 adult mice were housed in filtered cages with corn cob bedding and provided with standard chow and drinking water ad libitum. Cages were changed at least once a week in the laminar air-flow cabinet. Mice were subjected to 12 h of light/ 12 h dark cycles. Arhgef2^TM1^ mice were previously described^15^. 207fl/fl mice were created with loxP site insertion before exon 1 and after exon 4 of Arhgef2-207 without affecting transcriptional start sites for other ARHGEF2 isoforms. To eliminate all functional Arhgef2 isoforms, we also inserted loxP sites upstream and downstream of exons that encode for the common domains of all Arhgef2 isoforms before exon 2 and after exon 22 of *Arhgef2*-201, creating 201/207fl/fl mice. 207fl/fl or 201/207fl/fl mice were crossed with mice expressing tamoxifen (TAM)-inducible Cre recombinase under the control of the villin1 promoter, i.e., *Vill-CreERT2* mice (The Jackson Laboratory, 020282), to obtain 207Vil and 207KO after TAM treatment. Both 207KO and 207fl/fl mice were treated with TAM for 5 consecutive days, and small intestinal epithelial cells were isolated and analyzed 7-15 days later. Gene targeting was carried out in the Transgenic Technology Center Core at UTSW. C57BL/6 wild-type mice were from Jackson Laboratory. Recombination was induced by intraperitoneal injection of tamoxifen (MilliporeSigma, Cat# C8267) (20 mg/mL in corn oil) at a dose of 75 mg/kg body weight for five consecutive days. Tissues were collected 7–15 days post-induction. Wild-type C57BL/6 mice were obtained from Jackson Laboratory.

### Isolation of primary IECs

5 cm of small intestine was dissected, inverted and washed in PBS. Tissues were incubated in 2 mM EDTA in PBS for 30 min on a rotator at 4 °C. Epithelial cells were isolated by repeated mechanical dissociation in 0.1% Bovine calf serum (BCS). This process was repeated 6 times, and fractions were collected from 4–6 rounds. IECs were collected by centrifugation.

### ARHGEF2 immunoprecipitation–mass spectrometry

Primary intestinal epithelial cells (IECs) were isolated from untreated and tamoxifen-treated 207^Villin-Cre mice. A 5-cm segment of the small intestine was dissected, inverted, and washed with PBS. The tissue was incubated in PBS containing 2 mM EDTA for 30 min on a rotator at 4 °C. Epithelial cells were released by six rounds of mechanical dissociation in 0.1% bovine calf serum (BCS). IEC-enriched fractions from rounds 4–6 were collected, pooled, and recovered by centrifugation. For immunoprecipitation, IECs were lysed on ice for 20 min in IP lysis buffer (Thermo Fisher Scientific, Cat. No. 87787). Lysates were cleared by centrifugation, and total protein concentrations were determined using the Pierce BCA Protein Assay Kit (Thermo Fisher Scientific, Cat. No. 23227). Lysates were precleared with protein G agarose (Thermo Fisher Scientific, Cat. No. 20423) for 1 h at 4 °C. Precleared lysates were then incubated overnight at 4 °C with a rabbit antibody directed against mouse ARHGEF2 residues 57–76 (PAC#17843/4; Pacific Immunology) or rabbit anti-GEFH2-201 (aa 656–1000; Abcam, Cat# ab155785), followed by incubation with protein G agarose for 4 h with rotation. The beads were washed three times with IP lysis buffer, and bound proteins were eluted in 1× SDS sample buffer by heating at 95 °C for 10 min. Immunoprecipitated proteins were resolved by SDS–PAGE, and the gel was stained with Coomassie staining solution (Bio-Rad, Cat. No. 1610436). The upper band at approximately 130 kDa or the lower band at approximately 110 kDa was excised for mass-spectrometric analysis. Proteins within the excised gel band were reduced with dithiothreitol (DTT), alkylated with iodoacetamide (Sigma-Aldrich), and digested overnight with elastase (Worthington). Extracted peptides were purified by solid-phase extraction using an Oasis HLB plate (Waters). For liquid chromatography–tandem mass spectrometry, 2 µL of each peptide sample was injected into a Q Exactive HF mass spectrometer coupled to an Ultimate 3000 RSLC-Nano liquid chromatography system. Peptides were loaded onto a 75-µm-inner-diameter, 15-cm EasySpray column (Thermo Fisher Scientific) and eluted at a flow rate of 250 nL/min using a 90-min gradient from 0 to 28% buffer B. Buffer A contained 2% (v/v) acetonitrile and 0.1% formic acid in water, whereas buffer B contained 80% (v/v) acetonitrile, 10% (v/v) trifluoroethanol, and 0.1% formic acid in water. The mass spectrometer was operated in positive-ion mode with a source voltage of 2.5 kV and an ion-transfer-tube temperature of 275 °C. Full MS scans were acquired in the Orbitrap at a resolution of 120,000. For each full scan, up to 20 MS/MS spectra were acquired by higher-energy collisional dissociation of precursor ions with charge states of 2–8. Dynamic exclusion was set to 20 s after an ion was selected for fragmentation. Raw mass-spectrometry data were analyzed using Proteome Discoverer version 2.4 SP1 (Thermo Fisher Scientific). Peptides were identified with Sequest HT using a non-specific cleavage search against the Mus musculus UniProt protein database supplemented with the sequences of ARHGEF2 isoforms not represented in the database. Precursor-ion and fragment-ion mass tolerances were set to 10 ppm and 0.02 Da, respectively. Carbamidomethylation of cysteine was specified as a fixed modification, and oxidation of methionine was specified as a variable modification. Peptide identifications were filtered using a false-discovery rate cutoff of 1%.

### Analysis of FITC-dextran in the intestine and serum

Mice were administered 0.25 mg/g body weight of Fluorescein isothiocyanate–dextran (FITC-dextran; Sigma-Aldrich Cat# 46944) in PBS by oral gavage. After 4 h, mice were anesthetized, and blood was collected by cardiac puncture, and small intestines were harvested for live tissue imaging by confocal microscopy. During 4 h of the experiment, mice were deprived of food and water. Presence of FITC-dextran in the serum was analyzed by spectrophotometric measurement on Multi-Mode Microplate Reader (Synergy HT) at 485/528 nm (excitation/emission). A Leica Stellaris confocal microscope was used for image acquisition.

### RNA sequencing and analysis

Total RNA was isolated from IECs from wild-type mice, using a RNeasy Micro kit (Qiagen). Libraries were synthesized using TruSeq Stranded mRNA sample preparation kit from 500 ng of purified total RNA and indexed adapters according to the manufacturer’s protocol (Illumina). The final libraries were quantified using a Qubit fluorometer (Agilent Technologies), and RT-qPCR was performed using the Kapa Biosystems library quantification kit according to the manufacturer’s protocol. Pooled libraries were subjected to 35 bp paired-end sequencing according to the manufacturer’s protocol (Illumina NextSeq 500). The targeted sequencing depth was set at 30 million paired-end reads per sample. Blc2fastq2 Conversion software (Illumina) was used to generate demultiplexed Fastq files. The adapters were trimmed using Trim Galore (v0.6.4). The trimmed reads were aligned to the mouse genome (mm10) using STAR (2.7.3a). Subsequently, the mapped reads were quantified using the featureCounts of the Subread (v1.6.3) package. Genes with low expression (genes with an expression value of zero in more than 30% of the samples) were removed before subsequent analysis. Gene expression was normalized using the Voom method in the R package “limma” (v 3.50.3). Differentially expressed genes were also identified using this software. Gene set enrichment analysis of differentially expressed genes was performed using the R package “clusterProfiler” (v4.2.2). SeqMonk Mapped Sequence Data Analyzed 1.48.1 was used for DEseq2 analysis and initial visualization. Heat maps were generated using the R package heatmap (v1.0.12).

### Real-time quantitative-PCR

Total RNA was isolated using the RNeasy Mini Plus kit (Qiagen, Cat# 4134). cDNA was prepared from RNA using a PrimeScript RT Reagent cDNA Synthesis Kit (Takara, Cat# RR0373a). Real-time qPCR was performed using PowerTrack SYBR Green master mix (Applied Biosystems, Cat# A46109) with gene-specific primers, and relative expressions were calculated using the ΔΔCT method^42^. Gene expression was normalized to *GAPDH*. All experiments were repeated at least twice. The specific primer sequences are listed in the Tab. S8.

### Immunostaining

Formalin-fixed paraffin-embedded (FFPE) sections of mouse and human small intestine were baked (60 °C, 30 min), deparaffinized in xylene, and rehydrated through graded ethanol. Antigen retrieval was performed in citrate buffer (10 mM, pH 6.0) at 95 °C or Tris-EDTA buffer (10 mM Tris, 1 mM EDTA, pH 9.0) at 110 °C for 10 min. Sections were permeabilized with 0.25% Triton X-100 and blocked with 5% normal donkey serum. Slides were incubated overnight at 4 °C with primary antibodies against target proteins anti-Nectin (Invitrogen, Cat# PA5-4744, 1:1000), anti-Afadin (Santa Cruz Biotechnology, Cat# 74433, 1:200) followed by incubation with fluorescent secondary antibodies i.e., Alexa Fluor 488, 594, or 647 (ThermoFisher) for confocal imaging, and Abberior STAR RED (Cat# STRED-1051-500UG, 1:200) and Abberior STAR ORANGE (Cat# STORANGE-1001-500UG, 1:200) conjugates were used for STED microscopy. Nuclei were counterstained with DAPI and mounted in Abberior Mount Solid Antifade medium. For confocal microscopy, ProLong Gold Antifade Mountant (Invitrogen) was used. STED conditions for Figs. 3a, b, S2a, and 2b, c are available in the supplementary data. Colocalization studies were carried out in Imaris image analysis software after image acquisition using a Leica Stellaris confocal microscope or Abberior Facility Line STED microscope.

### Western blots and immunoprecipitations

Specific antibodies detecting AA6-28 of human ARHGEF2-219 and AA57-76 of mouse Arhgef2-207 were used to detect the long isoforms. An antibody raised against AA656-1000 of human ARHGEF2 was used to detect human and mouse long and short isoforms. BMDMs were lysed in RIPA buffer (10 mM Tris-HCl, pH 8.0, 1 mM EDTA, 0.5 mM EGTA, 1% Triton X-100, 0.1% sodium deoxycholate, 0.1% SDS, 140 mM NaCl) supplemented with protease and phosphatase inhibitors (Thermo Fisher, Cat#78430). Lysates were resolved by SDS–PAGE and transferred onto PVDF membranes. Membranes were blocked with 5% milk or BSA and incubated overnight at 4 °C with primary antibodies. Antibodies used included rabbit anti-GEFH2-201 (aa 656–1000; Abcam, Cat# ab155785), and custom rabbit antibodies against Arhgef2-207 (aa 57–76, Pacific Immunology corp., Cat# PAC17843/4) and ARHGEF2-219 (aa 6–28, Pacific Immunology corp., Cat# PAC17845/6), anti- LC3-I (Novus Biologicals, Cat# NB100-2331, 1:50), anti- LC3-II (Cell Signaling Technology, Cat# 4108S, 1:100), anti-ATP1A1 (Cell Signaling Technology, Cat# 3010 s, 1:1000), anti-ATP1B1(Cell Signaling Technology, Cat# 44759S, 1:1000), anti- Cingulin (Proteintech Cat# 21369-1AP, 1:5000), Anti- E-Cadherin (R&D Systems, Cat# AF748, 1:500), anti- ZO-1 (Invitrogen, Cat# 33-9100, 1:1000), anti- Occludin (Cell Signaling Technology, Cat# 68534S), anti- P-IRF5 ((Invitrogen, Cat# PA5-64760, 1:1000), anti- IRF5 (Cell Signaling Technology, Cat# 4950S, 1:1000), anti- P-IKKe (Cell Signaling Technology, Cat# 8766S, 1:1000), anti-IKKe (Cell Signaling Technology, Cat# 2905 P, 1:1000), anti-P-STAT1 (Cell Signaling Technology, Cat# 7649 P, 1:1000), anti-STAT1 (Cell Signaling Technology, Cat# 9172, 1:1000), anti- STING (Cell Signaling Technology, Cat# 13647S, 1:1000), anti-P62 (Cell Signaling Technology, Cat# 5114 T, 1:1000), anti-GAPDH (Cell Signaling Technology, Cat# 5174 T, 1:2000) followed by secondary antibodies, anti-rabbit IgG-HRP (Cell Signaling Technology, Cat# 7074, 1:3000), and anti-mouse IgG-HRP (Cell Signaling Technology, Cat# 91196, 1:3000) and anti-rat IgG-HRP (Cell Signaling Technology, Cat# 7077, 1:3000)). Detection was performed using enhanced chemiluminescence (Immobilon Western, Millipore, Cat# WBKLS010). Antibodies used included rabbit anti-GEFH2-201 (aa 656–1000; Abcam, Cat# ab155785), and custom rabbit antibodies against Arhgef2-207 (aa 57–76, Pacific Immunology Corp., Cat# PAC17843/4) and ARHGEF2-219 (aa 6–28, Pacific Immunology Corp., Cat# PAC17845/6). Additional antibodies are listed in the Tab. S5. For immunoprecipitation, cells were lysed on ice for 20 min in IP lysis buffer (Thermo Fisher Scientific, Cat# 87787), cleared by centrifugation, and preincubated with protein G agarose (Thermo Fisher Scientific, Cat# 20423) for 1 h at 4 °C. Precleared lysates were incubated overnight at 4 °C with primary antibodies, followed by 4 h rotation with protein G agarose. Beads were washed three times with lysis buffer and eluted in 1× SDS sample buffer at 95 °C for 10 min before immunoblot analysis.

### Densitometric analysis of immunoblots

Immunoblot signals were quantified by densitometry using ImageJ (NIH). For each protein and corresponding GAPDH loading controls, regions of interest were manually selected using identical dimensions across all samples. The background signal was subtracted from each band using a local background region. Protein levels were normalized to GAPDH and expressed relative to control conditions.

### Mouse small intestinal and colonic organoid culture

Mouse small intestinal and colon organoids were derived as previously described^43–45^. Small intestinal (15–20 cm) and colonic (3–6 cm) segments were harvested, flushed with cold PBS, opened longitudinally, and washed repeatedly until it gets clear. Tissue was cut into ~2 mm pieces and washed 15–20 times in cold PBS to remove debris. Fragments of the small intestine were incubated in Gentle Cell Dissociation Reagent (StemCell Technologies) for 15, 20 min and the colon for 20 min on a rocking platform at room temperature. Released crypts were filtered (70 µm), centrifuged (290 × *g*, 5 min, 4 °C) and washed in PBS + 0.1% BSA. Crypt-enriched fractions were resuspended in DMEM/F-12 (15 mM HEPES) and mixed 1:1 with Matrigel. Approximately 500 crypts were plated per well in a 24-well plate as domes and polymerized for 10 min at 37 °C. Cultures were maintained in complete epithelial organoid growth medium and supplemented with fresh medium twice weekly. For TAM-induced gene ablation, organoids were treated with 250 nM Z-4-hydroxytamoxifen (Sigma-Aldrich, Cat# H7904) for 3 days, followed by medium exchange to Intesticult medium without Z-4-hydroxytamoxifen.

### Organoid viability assay

Small-intestinal epithelial organoids were cultured at a density of 100 organoids per well in 24-well plates. Organoids were treated with 10 µM 4-hydroxytamoxifen (Millipore Sigma, Cat# H6278) for 0, 24, 48, and 72 h. The organoids were collected at each time point, and cell viability was assessed using the CellTiter-Glo Luminescent Cell Viability Assay (Promega, Cat# G7570) according to the manufacturer’s instructions.

### Human organoid culture

Human small intestinal and colon tissue samples were collected in the Division of Digestive and Liver Diseases at the University of Texas Southwestern Medical Center under the IRB Protocol Number STU-112010-130, “Registry and Biorepository for the study of Gastrointestinal Inflammatory Diseases”. Epithelial organoids were established from endoscopic biopsies of the ascending colon obtained with informed consent from de-identified donors. Briefly, biopsies were digested in 2 mg/mL collagenase IA, filtered through a 70 µm strainer, and centrifuged (150 g, 5 min). The epithelial fraction was resuspended in cold wash medium (DMEM/F12 50:50 with 10% FBS, 1× pen/strep) and embedded in 30 µL Matrigel. After polymerization (10 min, 37 °C, inverted), 450 µL of complete epithelial organoid medium was added, supplemented for the first 48 h with CHIR 99021 (2.5 µg/mL) to activate Wnt/β-catenin signaling. ROCK inhibitor (Y-27632) and TGFBR1 inhibitor were included to prevent anoikis and to suppress serum-derived TGF-β activity. Cultures were passaged 1:1–1:2 depending on organoid density and morphology. Images of organoid cultures were taken on day 5 with a Zeiss inverted microscope.

### AlphaFold modeling and interface analysis

Protein complex structures for wild-type and mutant 219N–ACTG1 assemblies were predicted using AlphaFold-Multimer with default parameters and 10 recycling iterations. For each condition, the highest-ranked model was selected based on the AlphaFold ranking score and analyzed without further structural refinement. Model-level confidence metrics (including ipTM, pTM, ranking score, and predicted disorder fraction) were taken directly from the AlphaFold summary files. Inter-chain interface analysis was performed directly on the coordinate models using a fixed geometric criterion: any atom–atom pair between 219 N (chain A) and ACTG1 (chain B) separated by ≤4.0 Å was classified as an inter-chain contact. Residue-level interface assignment required at least one qualifying atomic contact. Per-residue confidence (pLDDT) values were extracted from the atomic B-factor field of the AlphaFold models and averaged across atoms within each residue. Identical analysis procedures and distance thresholds were applied to wild-type and mutant models to permit direct comparison. All reported interface residues and contact counts derive strictly from these coordinate-based measurements without post hoc filtering or manual adjustment.

Visualization was carried out in PyMOL using cartoon representations for both chains. 219 N was rendered as a colored ribbon, with residues 55–71 highlighted to denote the predicted interface segment. ACTG1 was displayed as a scaffold to emphasize the relative placement of the 219 N chain. Identical camera orientation, lighting, and rendering parameters were applied to wild-type and mutant models to enable direct geometric comparison. All structural interpretations are based on coordinate-level visualization of the AlphaFold-predicted assemblies without additional refinement.

### Transfections

CaCo-2 and ARPE-19 cells were cultured with Dulbecco’s Modified Eagle Medium high glucose supplemented with 10% Fetal Bovine Serum (FBS) and 1% pen/strep stock, 10,000 U/mL pen, 10 mg/mL strep at 37 °C in a 5% CO2 incubator until they were 60-70% confluent. Cells were plated in an 8-well chambered slide (6 × 10^4^ per well). Cells were transfected with plasmid DNA using Lipofectamine 3000 (Thermo Fisher Scientific; Cat# L3000001) according to the manufacturer’s instructions. 500 ng of plasmid DNA was diluted in Opti-MEM medium and mixed with Lipofectamine 3000 reagent. The complex was added to the cells. The cells were analyzed 24 h post-transfection, and further assays were performed.

### Lentivirus construction and gene transfer

N-terminal fragment (AA1- 170) of the human ARHGEF2 isoform 219, consists of either WT or two mutated variants, was fused to the C-terminus of TagBFP2 via a (G_4_S)_3_ flexible linker to maintain conformational flexibility. The fusion cassette was cloned into a third-generation lentiviral transfer vector using Gibson assembly (NEB, E2611). Plasmid DNA was amplified in E*. coli* DH5α and purified using the ZymoPURE Plasmid Miniprep Kit (Zymo Research, Cat. No. D4209). The integrity of the final construct was confirmed by Sanger sequencing.

For lentivirus production, HEK293T cells were co-transfected with the lentiviral transfer plasmid, the packaging plasmid pPAX2 (Addgene, Cat#12260), and the envelope plasmid pMD2.G (Addgene, Cat#12259) using the TransIT-X2 Dynamic Delivery System (Mirus Bio, MIR 6003) at a DNA ratio of 2:1:1 (transfer:packaging:envelope). After 24 h, viral supernatants were collected and filtered through a 0.2 µm PES membrane to remove cellular debris. Filtered supernatants were used immediately to infect target cells. ARPE-19 cells were transduced with lentivirus encoding TagBFP2-LIR domain in the presence of 8 µg/mL polybrene, followed by incubation for 48 h for protein analysis and immunoprecipitations.

### *L. monocytogenes* infection of intestinal organoids

*Listeria monocytogenes* serotype 1/2a (FDA strain LS808; Microbiologics, Cat# 01249UV-V) was freshly prepared at 5 × 10^7^ CFU/mL. For infection, 10 µL of bacterial suspension (5 × 10^5^ CFU) was added to each well of a 24-well plate containing approximately 100 cultured gut epithelial organoids. Organoids were washed after 2 h and harvested at 0, 4, 6, 8, and 18 h post-infection for RNA isolation or immunoprecipitation/western blot analysis.

### *L. monocytogenes* infection of mice

Mice were orally gavaged with 1 × 10^8^ CFU of GFP-expressing L. monocytogenes (serotype 1/2a, FDA strain LS808). At 24 h post-infection, animals were euthanized by CO₂ asphyxiation, and livers and spleens were harvested. Tissues were homogenized in PBS, serially diluted, and plated on LB–erythromycin agar. Oral infections were carried out and GFP-positive bacterial colonies enumerated as described previously^46^.

### *L. monocytogenes* infection of transfected cells

Caco-2 cells were infected with *L. monocytogenes* at a multiplicity of infection (MOI) of 10. Bacteria were grown to mid-log phase (OD_600_ ≈ 0.5), diluted in infection medium, and added to cells for 2 h. Following infection, cells were washed and incubated for an additional 2 h to allow bacterial invasion. Cells were then fixed in 4% paraformaldehyde (PFA) for 15 min, washed twice with PBS, and stained with DAPI.

Confocal images were acquired using a Leica Stellaris 8 microscope (Leica Microsystems) with a 63 × oil immersion objective at a resolution of at least 2048 × 2048 pixels. Multichannel imaging (DAPI, EGFP, and mRFP) was performed with well-separated emission spectra, and channels were scanned sequentially to prevent fluorophore crosstalk.

### CUT&Tag analysis

CUT&Tag libraries for Pol II were generated from human intestinal organoids exposed to vehicle or *L. monocytogenes* (3 biological replicates). Nuclei were isolated from *Listeria*-infected and control human intestinal organoids disrupted by gentle douncing in NE1 buffer. CUT&Tag was performed on 100,000 nuclei per sample following the established protocol^47^. Briefly, nuclei were immobilized on concanavalin A-coated magnetic beads and incubated overnight at 4 °C with anti-RPB1 (Pol II) antibody (Cell Signaling Technology Cat# 2629) in digitonin-containing antibody buffer. After washing, a mouse secondary antibody and pre-loaded pAG-Tn5 adapter complex (EpiCypher) were sequentially applied, followed by tagmentation at 37 °C for 1 h. DNA was released by heat in SDS buffer, which was then neutralized, and purified in spin columns. Libraries were generated using NEBNext High-Fidelity PCR Master Mix and Nextera i5/i7 indexed primers, amplified for 11 cycles, size-selected with AMPure beads, quantified and assessed on a TapeStation, pooled equimolarly, and sequenced on the NovaSeq X Plus (PE150; ~20 million reads/sample). Reads were aligned to hg38 and were filtered to remove PCR duplicates and ENCODE blacklist regions. Then, reads were converted into normalized genome-wide coverage tracks. Two ARHGEF2 promoters (P1 and P2) were defined from Pol II enrichment and CAGE TSS clusters and represented as ±1 kb windows around their Pol II summits. Pol II signal for each sample was quantified as the average normalized signal across all bins within each promoter window. Condition-specific means and variability were calculated, and differences between control and *Listeria* conditions were evaluated using Welch’s t-test. Promoter-level changes were visualized using averaged Pol II profiles and bar plots (mean ± s.e.m.) with associated log_2_ fold-changes and *P* values.

### Inhibitor treatments

48 h post-transfected ARPE-19 cells and colon epithelial organoids derived from wild-type and *Arhgef2*-207-villin-Cre/ERT2 mice were treated with inhibitors using two different concentrations to target distinct pathways. The RhoA pathway was inhibited with 10 µM or 50 µM Y-27632 (Selleck Chemicals Cat#1254), the STING pathway was inhibited with 0.1 mM and 1 mM H-151 (Sigma- Aldrich Cat# SML2437), and autophagy was modulated by 10 µM SAR405 (Sigma- Aldrich, Cat# AMBH303C4EB3), 100 nM Wortmannin (Sigma-Aldrich, Cat# 681675), and 200 nM Bafilomycin A1 (Sigma-Aldrich, Cat# AABH30371C0C) and were incubated for 4 h. After treatment, cells were washed with PBS and lysed in RIPA buffer supplemented with protease and phosphatase inhibitors (Roche) for downstream protein analysis.

### Quantification and statistical analysis

Image analysis and three-dimensional reconstructions were performed using Imaris (Oxford Instruments). Statistical tests, the number of biological samples, and the definitions of error bars are specified in the corresponding figure legends. Biological experiments were independently repeated at least twice with consistent results, unless otherwise indicated. RNA-seq and CUT&Tag datasets were generated in a single experiment using duplicate or triplicate biological samples, as specified in the corresponding figure legends. No statistical method was used to predetermine sample size. No data was excluded from the analysis. The experiments were not randomized. The investigators were not blinded to allocation during experiments and outcome assessment. Antibodies, primers, and chemicals used in this study are listed in the Key Resources Table (Supplementary Data 8).

### Reporting summary

Further information on research design is available in the >Nature Portfolio Reporting Summary linked to this article.

## Supplementary information

Supplementary Information

Description of Additional Supplementary Files

Supplementary Data 1

Supplementary Data 2

Supplementary Data 3

Supplementary Data 4

Supplementary Data 5

Supplementary Data 6

Supplementary Data 7

Supplementary Data 8

Reporting Summary

Transparent Peer Review file

## Source data

Source Data

## Data Availability

The accession numbers for published datasets analyzed in this study are listed in the key resources table (Supplementary Data 8). https://www.ncbi.nlm.nih.gov/geo/query/acc.cgi?acc=GSE311695. The data generated in this study are provided in the Supplementary Source Data File. Deposited in the Texas University Southwestern Research Data Repository. The DOI is listed in the key resources table. https://doi.org/10.18738/T8/OMPEZK. Mass Spectrometry data is available at the Mass Spectrometry Interactive Virtual Environment (MassIVE) under submission number MSV000103076. https://massive.ucsd.edu/ProteoSAFe/dataset.jsp?task=59989519292f4f0f9d1ed99270657212. Cell lines generated in this study are available upon request to the lead contact. Any additional information required to reanalyze the data reported in this paper is available from the lead contact upon request. This paper does not report original code. Source data are provided with this paper.
