# Supplementary material for: ARHGEF2 isoform switching couples intestinal epithelial barrier function with autophagic host defense response to pathogens
Source: Nat Commun. 2026 Sep 24;17:11103. doi: 10.1038/s41467-026-78030-w (PMC13696713; doi:10.1038/s41467-026-78030-w)
Supplement: Supplementary file 1 [file 41467_2026_78030_MOESM11_ESM.pdf]

nature portfolio

Corresponding author(s): Reinecker, Hans-Christian

Last updated by author(s): 9/4/2026

Reporting Summary

Nature Portfolio wishes to improve the reproducibility of the work that we publish. This form provides structure for consistency and transparency in reporting. For further information on Nature Portfolio policies, see our Editorial Policies and the Editorial Policy Checklist.

Statistics

For all statistical analyses, confirm that the following items are present in the figure legend, table legend, main text, or Methods section.

n/a	Confirmed	
☐	☒	The exact sample size (*n*) for each experimental group/condition, given as a discrete number and unit of measurement
☐	☒	A statement on whether measurements were taken from distinct samples or whether the same sample was measured repeatedly
☐	☒	The statistical test(s) used AND whether they are one- or two-sided<br>*Only common tests should be described solely by name; describe more complex techniques in the Methods section.*
☐	☒	A description of all covariates tested
☐	☒	A description of any assumptions or corrections, such as tests of normality and adjustment for multiple comparisons
☐	☒	A full description of the statistical parameters including central tendency (e.g. means) or other basic estimates (e.g. regression coefficient) AND variation (e.g. standard deviation) or associated estimates of uncertainty (e.g. confidence intervals)
☐	☒	For null hypothesis testing, the test statistic (e.g. *F*, *t*, *r*) with confidence intervals, effect sizes, degrees of freedom and *P* value noted<br>*Give P values as exact values whenever suitable.*
☒	☐	For Bayesian analysis, information on the choice of priors and Markov chain Monte Carlo settings
☒	☐	For hierarchical and complex designs, identification of the appropriate level for tests and full reporting of outcomes
☒	☐	Estimates of effect sizes (e.g. Cohen's *d*, Pearson's *r*), indicating how they were calculated

Our web collection on statistics for biologists contains articles on many of the points above.

Software and code

Policy information about availability of computer code

Data collection	Microscope: DMi8 microscop system (Leica Microsystems) RNAseq: Illumina NextSeq 500, Western Blot: ChemiScope 3300; RT-PCR: LightCycler 480
Data analysis	Prism software (GraphPad 9), R (Rstudio), Limma package version 3.50.3, STAR (2.7.3a), Subread (v1.6.3) package, DESeq2 1.36.0, ClusterProfiler 4.2.2, ggplot2 3.5.1, pheatmap (v1.0.12)

For manuscripts utilizing custom algorithms or software that are central to the research but not yet described in published literature, software must be made available to editors and reviewers. We strongly encourage code deposition in a community repository (e.g. GitHub). See the Nature Portfolio guidelines for submitting code & software for further information.

Data

Policy information about availability of data

All manuscripts must include a data availability statement. This statement should provide the following information, where applicable:

- Accession codes, unique identifiers, or web links for publicly available datasets
- A description of any restrictions on data availability
- For clinical datasets or third party data, please ensure that the statement adheres to our policy

We have included Data availability section in our manuscript, and all GEO accssion are public after publication. All raw and processed sequencing data will be available

Research involving human participants, their data, or biological material

Policy information about studies with human participants or human data. See also policy information about sex, gender (identity/presentation), and sexual orientation and race, ethnicity and racism.

Reporting on sex and gender	No sex- or gender-based analysis was performed.
Reporting on race, ethnicity, or other socially relevant groupings	N/A Annoymized samples. Biopsies were obtained from 20 patients during routine colonoscopies after informed consent.
Population characteristics	N/A Annonymized samples.
Recruitment	Small intestinal and colon biopsies were obtained after consent at the Division of Digestive and Liver Diseases at UTSW
Ethics oversight	University of Texas Southwestern Medical Center under the IRB Protocol Number is STU-112010-130.

Note that full information on the approval of the study protocol must also be provided in the manuscript.

Field-specific reporting

Please select the one below that is the best fit for your research. If you are not sure, read the appropriate sections before making your selection.

☒ Life sciences ☐ Behavioural & social sciences ☐ Ecological, evolutionary & environmental sciences

For a reference copy of the document with all sections, see nature.com/documents/nr-reporting-summary-flat.pdf

Life sciences study design

All studies must disclose on these points even when the disclosure is negative.

Sample size	Sample sizes for all experiments were chosen based on our previous experience. (Bersch KL etal 2021, Park SM et al, 2019, Kashyap AS 2019, Zhao Y et all 2019)
Data exclusions	No data were excluded from the showed experiments.
Replication	A representative or a pool of at least two independent experiments showing similar results have been conducted. And all attempts at replication were reproducible.
Randomization	All sample involved were allocated into experimental groups randomly.
Blinding	The investigators were not blinded to group allocation during collection and analysis. The approach is considered standard for experiments of the type performed in this study, as the genetic background of mice must be predetermined prior to analysis. For cell experiments, the genetic background and treatment of the cells must be predetermined prior to analysis.

Reporting for specific materials, systems and methods

We require information from authors about some types of materials, experimental systems and methods used in many studies. Here, indicate whether each material, system or method listed is relevant to your study. If you are not sure if a list item applies to your research, read the appropriate section before selecting a response.

Materials & experimental systems

n/a	Involved in the study	
☐	☒	Antibodies
☐	☒	Eukaryotic cell lines
☒	☐	Palaeontology and archaeology
☐	☒	Animals and other organisms
☒	☐	Clinical data
☒	☐	Dual use research of concern
☒	☐	Plants

Methods

n/a	Involved in the study	
☒	☐	ChIP-seq
☒	☐	Flow cytometry
☒	☐	MRI-based neuroimaging

Antibodies

Antibodies used	Sheep anti mouse GEFH1, Exalpha Biologicals, Shirley, MA Cat# X1089

Antibodies used	Rabbit anti-GEFH1, Abcam, Waltham, MA Cat# ab155785<br>Rabbit anti- Mouse Arhgef2(57-76), Pacific immunology corp. Ramona, CA Cat# PAC17843/4<br>Rabbit anti-Human Arhgef2(6-28), Pacific immunology corp. Ramona, CA Cat# PAC17845/6<br>Rabbit anti-GAPDH, Cell Signaling Technologies, Danvers, MA Cat# 92310<br>Mouse anti-ZO-1 (1A12), Invitrogen, Carlsbad, CA Cat# 33-9100<br>Rat anti-Occludin (6B8A3), Cell Signaling Technologies, Danvers, MA Cat# 68534<br>Rabbit anti-Claudin-2 (E1H9O), Cell Signaling Technologies, Danvers, MA Cat# 48120<br>Rabbit anti- Claudin3, Invitrogen, Carlsbad, CA Cat# 34-1700<br>Goat anti- E-Cadherin, R&D Systems, Minneapolis, MN Cat# AF748-SP<br>Rabbit anti-b-Catenin (D10A8), Cell Signaling Technologies, Danvers, MA Cat# 8480<br>Goat anti-Nectin 3, Invitrogen, Carlsbad, CA Cat# PA5-47441<br>Goat anti- Nectin3, R&D Systems, Minneapolis, MN Cat# AF3064-SP<br>Rabbit anti- Afadin (D1Y3Z) Cell Signaling Technologies, Danvers, MA Cat# 13531T<br>Rabbit anti-ATP1A1, Cell Signaling Technologies, Danvers, MA Cat# 3010S<br>Rabbit anti-ATP1B1, Cell Signaling Technologies, Danvers, MA Cat# 44759S<br>Mouse anti- α-Tubulin (DM1A), Cell Signaling Technologies, Danvers, MA Cat# 3873P<br>Mouse anti-β-Actin (8H10D10), Cell Signaling Technologies, Danvers, MA Cat# 3700S<br>Rabbit anti-IRF5, Abcam, Waltham, MA Cat# 181553<br>Anti- Phospho-IRF5, Invitrogen, Carlsbad, CA Cat# PA5-64760<br>Rabbit anti- IKKε, Cell Signaling Technologies, Danvers, MA Cat# 2905P<br>Rabbit anti- Phospho- IKKε, Cell Signaling Technologies, Danvers, MA Cat# 8766S<br>Anti- STAT1, Cell Signaling Technologies, Danvers, MA Cat# 9172<br>Anti- Phospho-STAT1, Cell Signaling Technologies, Danvers, MA Cat# 7649P<br>Rabbit Anti- P65, Cell Signaling Technologies, Danvers, MA Cat# 8242S<br>Mouse anti- phospho-P65, Cell Signaling Technologies, Danvers, MA Cat# 13346S<br>Rabbit anti- STING, Cell Signaling Technologies, Danvers, MA Cat# 13647S<br>Rabbit anti-TBK1, Cell Signaling Technologies, Danvers, MA Cat# 3504<br>Rabbit anti- Phoshpo-TBK1, Cell Signaling Technologies, Danvers, MA Cat# 5483S<br>Rabbit anti-IL-1B, Cell Signaling Technologies, Danvers, MA Cat# 31202<br>Rabbit anti- ATG16L1, Cell Signaling Technologies, Danvers, MA Cat# 8089<br>Rabbit anti- BECLIN1, Cell Signaling Technologies, Danvers, MA Cat# 3495T<br>Rabbit anti-LC3 Cell Signaling Technologies, Danvers, MA Cat# 4107<br>Mouse anti- S6 Cell Signaling Technologies, Danvers, MA Cat# 2317<br>Rabbit Phospho- S6 Cell Signaling Technologies, Danvers, MA Cat# 4858<br>Rabbit anti- P70S6K Cell Signaling Technologies, Danvers, MA Cat# 34475T<br>Mouse anti- Phospho- P70S6K, Cell Signaling Technologies, Danvers, MA Cat# 9206T<br>Rabbit anti- MLKL, Cell Signaling Technologies, Danvers, MA Cat # 14993S<br>Anti-GSDMD, Proteintech, Rosemont, IL Cat# 20770-1-AP<br>Anti-CASPASE-1, Abcam, Waltham, MA Cat# Ab108362<br>Rabbit anti- cleaved CASPASE-3 (5A1E), Cell Signaling Technologies, Danvers, MA Cat# 9664<br>Rabbit anti- CASPASE-8(D35G2), Cell Signaling Technologies, Danvers, MA Cat# 4790S<br>Rabbit anti-MLKL (D2I6N), Cell Signaling Technologies, Danvers, MA Cat# 14993<br>Rabbit anti-RIP3 (D94C12), Cell Signaling Technologies, Danvers, MA Ca# 3493S<br>Anti-rabbit-Ig G-HRP, Cell Signaling Technologies, Danvers, MA Cat# 7074<br>Anti-mouse-Ig G-HRP, Cell Signaling Technologies, Danvers, MA, Cat# 7078<br>Anti-sheep-Ig G-HRP, Invitrogen, Carlsbad, CA Cat# A16041<br>Anti-goat-Ig G-HRP, Santa Cruz biotech., Dallas, TX Cat# SC-2354<br>Anti-Goat IgG AF488, Invitrogen, Carlsbad, CA Cat# A32814TR<br>Anti-Rabbit IgG AF594, Invitrogen, Carlsbad, CA Cat# A11037<br>Anti- Mouse IgG AF488, Invitrogen, Carlsbad, CA Cat# A-21202<br>Anti- Goat IgG AF647, Invitrogen, Carlsbad, CA Cat# A-21447<br>STAR Red goat anti-rabbit, Abberior Instruments GmbH, Gottingen, Germany Cat# STRED-1002-500UG<br>STAR Orange goat anti-mouse, Abberior Instruments GmbH, Gottingen, Germany Cat# STORANGE-1001-500UG<br>STAR Red Donkey anti-goat, Abberior Instruments GmbH, Gottingen, Germany Cat# STORANGE-1055-500UG
Validation	Abs were selected based on cross-reactivity with mouse and human (depending on our research needs) and carefully evaluated the validation data provided by both the manufacturers and literature. We performed further validation of antibodies by examining the molecular weight of the band by Western blotting and using KO cells for the protein of interest. Antibodies were aliquoted and stored as recommended by the manufacturer to minimize freeze thaw cycles and associated loss in performance.

Eukaryotic cell lines

Policy information about cell lines and Sex and Gender in Research

Cell line source(s)	ATCC: ARPE-19 (CRL-2302), HEK 293T (ATCC CRL-11268), Caco2 (HTB-37), T84 (CCL-248), HCT116 (CCL-247EMT), HT29 (HTB-38)
Authentication	Cell line authenticated by ATCC.

Mycoplasma contamination	Tested and found negative for mycoplasma
Commonly misidentified lines (See ICLAC register)	N/A

Animals and other research organisms

Policy information about studies involving animals; ARRIVE guidelines recommended for reporting animal research, and Sex and Gender in Research

Laboratory animals	From Jackson laboratory: C57/Bl6, The Jackson Laboratory (000664), Arhgef2-207fl/fl, Arhgef2-201fl/fl generated in this manuscript, Arhgef2TM1 were described in Chiang et al. PMID: 24270516, B6.Cg-Tg(Vil1-cre/ERT2)23Syr/J, The Jackson Laboratory (020282).
Wild animals	*Provide details on animals observed in or captured in the field; report species and age where possible. Describe how animals were caught and transported and what happened to captive animals after the study (if killed, explain why and describe method; if released, say where and when) OR state that the study did not involve wild animals.*
Reporting on sex	*Indicate if findings apply to only one sex; describe whether sex was considered in study design, methods used for assigning sex. Provide data disaggregated for sex where this information has been collected in the source data as appropriate; provide overall numbers in this Reporting Summary. Please state if this information has not been collected. Report sex-based analyses where performed, justify reasons for lack of sex-based analysis.*
Field-collected samples	n/a
Ethics oversight	University of Texas Southwestern Medical Center under the protocol number 2020-102946

Note that full information on the approval of the study protocol must also be provided in the manuscript.

Plants

Seed stocks	*Report on the source of all seed stocks or other plant material used. If applicable, state the seed stock centre and catalogue number. If plant specimens were collected from the field, describe the collection location, date and sampling procedures.*
Novel plant genotypes	*Describe the methods by which all novel plant genotypes were produced. This includes those generated by transgenic approaches, gene editing, chemical/radiation-based mutagenesis and hybridization. For transgenic lines, describe the transformation method, the number of independent lines analyzed and the generation upon which experiments were performed. For gene-edited lines, describe the editor used, the endogenous sequence targeted for editing, the targeting guide RNA sequence (if applicable) and how the editor was applied.*
Authentication	*Describe any authentication procedures for each seed stock used or novel genotype generated. Describe any experiments used to assess the effect of a mutation and, where applicable, how potential secondary effects (e.g. second site T-DNA insertions, mosiacism, off-target gene editing) were examined.*
