# Supplementary material for: ARHGEF2 isoform switching couples intestinal epithelial barrier function with autophagic host defense response to pathogens
Source: Nat Commun. 2026 Sep 24;17:11103. doi: 10.1038/s41467-026-78030-w (PMC13696713; doi:10.1038/s41467-026-78030-w)
Supplement: Supplementary file 2 [file 41467_2026_78030_MOESM12_ESM.pdf]

nature portfolio

Peer Review File

ARHGEF2 Isoform Switching Couples Intestinal Epithelial Barrier Function with Autophagic Host Defense Response to Pathogens

Corresponding Author: Professor Hans-Christian Reinecker

This file contains all reviewer reports in order by version, followed by all author rebuttals in order by version.

Version 0:

Reviewer comments:

Reviewer #1

(Remarks to the Author)
Summary:
The authors examine the role of the two major isoforms of ARHGEF2 (GEF-H1) through the use of specific antibodies, expression constructs and the generation of conditional knockout mice. Their findings indicate that the N-terminus of the long isoform functions as a structural interface between Afadin-Nectin complexes and the cytoskeletal network in intestinal epithelial cells. Loss of the long form of Arhgef2 in mice causes epithelial cell dysfunction, loss of barrier integrity, compensatory inflammation. Use of organoids from these mice reveals that epithelial cell phenotypes are intrinsic and do not require microbial triggers. Finally, authors show that infection of epithelial cells by an invasive pathogen (Listeria) is sufficient to cause changes in isoform expression that appear to contribute to loss of epithelial junction integrity.

This is a well designed study that has important implications for our understanding of how gene splicing and Rho regulation can impact epithelial junctions. The writing of the paper and presentation of the data could be improved in some areas (see minor comments below) but I am generally in support of publication.

Minor comments:
-nomenclature is challenging throughout this paper. Recommend dropping use of GEF-H1 after introduction.
-line 75, should be “ARHGEF2”, remove dash to be consistent
-line 78 a bit clunky, propose changing to “(Fig.1B), which encodes a unique…”
-Figure 1A does a good job of showing isoforms and domains. But the gray bars are not super obvious… maybe add a schematic of an antibody (also in gray) indicating that there are 4 different antibody epitopes (in different species) being depicted? For the other panels, can you indicate species to make it super clear what you are looking at? A label of the gene name at the top and numbering of amino acids would also be helpful.
-line 125, Fig.2I is cited twice.
-line 130 a bit clunky, merge into one paragraph?
-Figure 2D,I,L, where are the X-axis labels?
-Figure 3A vs 3G. For clarity, recommend indicating antibody used since NT epitope (3A) specific to -207 isoform but CT common epitope (3G) is not.
-Figure 3B/H, units are required for the graphs. Y-axis needs to be clarified… nm from what? Why measure vertical distance in B but horizontal in H?
-Figure 3L,M,N. Where is the RFP only control band? If running at a different MW then crop that portion of the gel for display.
-Figure 3K-N. Conserved motifs in K are highlighted but not tested in co-ip experiments, so their role remains unknown. Region of ARHGEF2 used for NT co-ip is not described in results or figure legend… which aa?
- Listeria should be italicised. Be consistent with nomenclature.
-Figure 4K, where are X-axis labels?
-line 288. RFP-tagged ARHGEF2-201 and LC3-GFP are shown to be colocalized with Listeria in Figure 5L. This is confusing since many papers show that Listeria can evade autophagy during infection. Are these bacteria trapped within vacuoles or a subset of total bacteria in the infected cells?
-line 299, “linking junctional targeting by L. monocytogenes to innate immune activation”. It is not clear that the pathogen is “targeting” expression of Arhgef2. For example, there are no virulence factors shown here to control changes in isoform

expression. It seems plausible that a host immune response is causing changes in isoform expression. Recommend changing conclusive statements about what is happening vs how. Same comment for line 327.
-line 343. “ARHGEF2 isoform regulation may represent a critical determinant of epithelial resistance”. Are there any conditions that promote expression of the long isoform over the shorter isoform e.g. in development, repair pathway, etc. ?

Reviewer #2

(Remarks to the Author)
This manuscript investigated the long and short isoforms of the guanine nucleotide exchange factor GEF-H1 in human organoids and mouse intestinal tissues. These are microtubule-associated molecular switch that activates Rho GTPases. The team developed antibodies that can distinguish these isoforms. They make conditional mouse knockouts for the long form Arhgef2-207, which corresponds to the human ARHGEF2-219 isoform. They show that this long form localizes to adherens junctions in polarized intestinal epithelial cells. Deleting Arhgef2-207 increased autophagy activity, increased gut permeability, elevated inflammatory cytokines, accompanied by an elevation in the short form Arhgef2-201. Through a number of Western blot and co-IP analysis, the authors show the short form interacts with STING and LC3. To show the relevance to human biology, they showed that organoids that are infected with Listeria monocytogenes showed reduced ARHGEF2-219 isoform but increased ARHGEF2-201, which they believe to trigger autophagy. The efforts on the biochemical characterization of binding partners of these isoforms are appreciated. There are some major issues with the experimental control, conclusion, interpretation, and quantification.

Major concerns:
1. Fig. 5 nicely showed that Listeria monocytogenes targeted the longer form while increasing the short form in biochemistry assay. Surprisingly, the manuscript ended without further testing if the conditional knockout mice would have an exacerbated inflammation by Listeria monocytogenes infection. The entire story stopped short by describing a changed abundance of an isoform that binds to different partners.
2. There are lots of Western blots or IP results in this manuscript, but they did not seem to be quantified. This raises major concerns especially the authors used these results for their major conclusions.
3. Some of the conclusions are overstated. For example, showing elevated cytokine levels by qPCR or RNA-seq may not be equivalent with spontaneous inflammation or an increased susceptibility of colitis. Whether these KO mice have increased colitis susceptibility has not been tested here.
4. The statement such as “loss of Arhgef2-207 reprograms IECs toward an autophagy dominant stress” shall be documented by stronger results.

Detailed comments:
Fig. 1B-C. Not clear why some were labeled as ARHGEF2 others were Arhgef2. If they refer to the antibodies used for the Western blot, the label should be modified.
Fig. C. “Importantly, ARHGEF2-219 expression was absent in human colorectal adenocarcinoma-derived cell lines Caco2, T-84, HCT116 and HT-29 that expressed the shorter 84 ARHGEF2-201 isoform instead (Fig 1C).” --- This conclusion was based on an antibody raised against the murine Arhgef2(656-1000). Why not use the ARHGEF2 antibody here?
“These mice maintained Arhgef2-207 expression in IECs but lacked shorter GEF-H1 isoforms in both IECs and macrophages (Fig. 1D),” ---- Here should be referring to Fig. 1E.
Fig. 1F, “ARHGEF2-219 was 97 detected in and adjacent to Nectin-Afadin complexes and in puncta throughout the cytoplasm of 98 IECs (Fig.1F).” ---- This is difficult to appreciate from the images.
“these findings reveal that differentiated intestinal epithelial cells express long ARHGEF2 isoforms that are lost in intestinal cancer cell lines” ---- Can the second part of this statement be supported by staining in monolayer of cancer cell lines?
Fig. 2A. The diagram includes three separate alleles can be confusing to readers.
Fig. 2D. Would be helpful to know where the primers are located, according to the diagram in Fig. 1A.
Fig. 2G. The label of TAM for one of the groups can be confusing, assuming the Arhgef2-207cko mice were also administrated with TAM.
Fig. 2J, 2K, 2M. “Deletion of Arhgef2-207 further caused the loss of the Na^+/K^+-ATPase subunits ATP1A1 and ATP1B1” ---- Normally, if you claim a loss of something, the blots should be quantified from multiple animals and experiments. Same comments for Fig. 2K and 2M.
Line 139: “Genetic ablation of Arhgef2-207 induces compensatory upregulation of Arhgef2-201, which is insufficient to restore barrier integrity, leading to spontaneous small intestinal inflammation and heightened susceptibility to colitis.” ---- This was overstated, as neither spontaneous inflammation nor colitis was seen at this point.
Fig. 3C. “The AJ consists of two concentric rings: an outer ring containing Nectin-Afadin complexes and an inner ring composed of E-cadherin–catenin assemblies” --- How is Arhgef2-207 relative to the inner ring?
Fig. 3I-J: The labels on the top of these blots are the same, and it was confusing what is different between the two? Since Arhgef2-201 is also expressed in wild type cells (Fig. 3G), does ZO1 also interact with Arhgef2-201 in wild type cells? It is not clear about the significance of I and J here, especially no quantitative comparison was made between the KO and WT.
“Arhgef2-201 is enriched at tight junctions (TJs) in association with ZO-1.” --- This does not change from WT to 207 KO.

Fig. 4E. “IECs from Arhgef2 207cKO mice formed enlarged intracellular compartments enriched for the tight junction protein ZO-1, which were absent in Arhgef2207vilc controls (Fig. 4E). ---- This statement is difficult to appreciate.
Fig. 4F, G: “These compartments colocalized with LAMP-1 and LC3, consistent with their identity as autophagosomes (Fig. 4F, G).” ---- Including the control here seems important.

Reviewer #3

(Remarks to the Author)
OVERALL ASSESSMENT:
In the submitted manuscript, the authors make the argument that the mouse/human protein Arhgef2/ARHGEF2 possesses two isoforms of differing lengths that interact with distinct sets of cell-junction proteins in intestinal epithelia; thus, affecting the integrity of the intestinal barrier. The authors argue that the N-terminal region of Arhgef2/ARHGEF2, present only in the long isoform, is responsible for these distinct interactions. Given the significant phenotypes associated with preferential expression of the short isoform, the authors performed transcriptomics in intestinal epithelial cells producing each isoform and identified several stress-response and degradative pathways – one of which being autophagy – enriched in cells only expressing the short isoform. The authors provide several pieces of data to demonstrate that markers for innate immune and autophagy responses increase after the switch in expression to the short isoform, consistent with their transcriptomics data. Lastly, the authors suggest that Listeria infection increases Pol II occupancy at the promoter site for the short isoform, which upon expression, interacts with autophagy-regulating protein, STING, as well as the autophagy protein, LC3. Altogether, the data presented suggest that bacterial infection alters the expression of Arhgef2/ARHGEF2 from a long to a short isoform, with the latter lacking interaction domains with the appropriate cell-junction proteins required for the maintenance of barrier integrity, causing downstream effects such as the induction of innate immune and autophagic responses.
While the authors have produced and characterized a mouse model that affects the activity of cell-junctional proteins that could benefit the field, additional pieces of data and controls are required to support several of their claims in this report.

MAJOR COMMENTS:
1. The writing of the text as well as the readability of the figures contain room for improvement. There are multiple grammatical errors throughout, references to the wrong figure panels or references to data that doesn’t exist in the figure suggesting a lack of rigor in preparation of the manuscript. In addition to these concerns, a simplified nomenclature for the long vs short isoforms of ARHGEF2 would significantly improve the flow of the manuscript and interpretation of the data in the figures, allowing readers to more efficiently identify the sample without relying on identifiers in a smaller superscript.
Additionally more minor recommendations for improved readability of figures include among other things:
a. Fig. 1BC – explicitly direct the reader to the relevant bands of interest – higher and molecular weight ARHGEF – using arrows
b. Fig. 2DFIL – label each color in the graph/axes instead of relying on the figure legend
c. Fig. 3BH – label each color in the graph/axes instead of relying on the figure legend
d. Fig. 4K – label each color for each bar in the graph.
2. The methodology section and some figure legends are severely lacking in detail in some cases making the data impossible to interpret. Some examples include how the viability of organoids was measured, details regarding Listeria infections (strain identity, MOI); timing of serum FITC-dextran quantification (collection of serum on different dates or Cre-induction was staggered to collect serum on the same date, etc.); depletion laser and power settings for STED microscopy and how lateral images were obtained and many many others. While these may seem like small issues they are in fact important issues. For example, it is know that Listeria has multiple mechanisms for evading autophagy including the use of actin based motility and phospholipases, did the authors use strains lacking these factors to see association with autophagy and if not how do they explain the discrepancy with the literature and how these bacteria were targeted by autophagy? Listeria doubles every 40 minutes within cells, how long were the organoids infected for and at what MOI, this is critical for considering whether phenotypes observed were due to overwhelming bacterial infection or if this was early during infection and likely a response to initial detection by pattern recognition receptors. These are just some examples of how the data is uninterpretable with no details provided at all about the experimental approaches and controls.
3. A reference control (ex. for another protein that regulates cell-junction integrity) to assess the effect sizes of various phenotypes (ex. losses in barrier integrity Figures 2) as well as the specificity for ARHGEF2 in the induction of autophagy and mucosal innate immunity (Figs. 4 and 5) should be included for key experiments. The authors state that these processes are regulated by ARHGEF2 isoforms but it seems more likely to me given that 10% of the genome is differentially regulated in their mutants that all of these downstream effects are caused by the loss of junction integrity and barrier function leading to stress responses. There are no other controls for junction dysfunction so it is impossible to know if everything observed is simply a pleiotropic effect of loss of junction function. There is also no justification provided for looking at autophagy and cell death pathways over any of the other ~2000 genes differentially expressed.
4. Evidence of absence for the opposing markers and isoforms are required to support the claims for isoform-specific compartmentalization of ARHGEF2 and their regulation of autophagy and innate immunity. Thus, all of the immunoprecipitation experiments should include blots for both isoforms (as the authors have shown for Figure 3I). Similarly, additional blots for the excluded junctional complex (ex. include an Afadin/Nectin-3 blot in Figure 3I or a ZO-1 blot in Figure 3D) are also required.
5. The authors repeatedly reference “specific” or “selective” changes caused by Listeria, yet the authors have not infected with other bacteria such as Salmonella or simulated infection using Internalin-deficient Listeria, attenuated Listeria (for example lacking LLO), heat-killed bacteria, or PAMPs. This figure is severely underdeveloped and overinterpreted without significant other controls to determine how Listeria or infection more generally might alter isoform expression.
6. While the idea that the switch from the long to short isoform of ARHGEF2 results in the activation of autophagy, the authors ought to consider investigating mTOR activity and lysosomal integrity in greater detail as the phenotypes described in Figures 4 and 5 can all be described by perturbations in these pathways/organelles rather than a specific effect on autophagy. Thus, at their present state, these final two figures do not significantly contribute to the story.

MINOR COMMENTS:
• Line 115: Figure call-out refers to 1D when it should be referring to Figure 2D.
• Line 178: This call-out regarding the expression of the RFP fusion constructs in the ARPE-19 cell line was probably meant to reference Figure 3L.
• Line 198: In the autophagy field, the use of the word "core" when describing autophagy genes and proteins is restricted to proteins that are absolutely required for autophagosome biogenesis. Foxo3 and Hif1a are regulatory proteins and are not typically described as core-autophagy genes. The authors also overlook Sqstm1, also called p62, in Fig. 4B. While not necessarily a "core" autophagy protein, Sqstm1 is a ubiquitously discussed protein in autophagy manuscripts.
• Line 283: Figure call-out likely refers to Figures 5H and 5I instead of 5G and 5J.
• Figure 1E is missing a call-out in the text.
• Figure 2H may be better organized by switching the order of the mouse genotypes to be consistent with all other graphs in this figure.
• Figure 3L-N ought to include a mutagenized version of the named structural motifs fused to RFP to demonstrate the importance of those motifs for interaction with afadin and Nectin-3. The authors use expression of this fragment of the protein to argue that these domains are important but did not do any mutagenesis of any of the domains to demonstrate that any of them or all of them are in fact important. Given that this figure is all done with expressed fusion proteins this is incredibly straightforward and simple to do.
• Figure 4FG are referenced in the discussion (Line 317) to demonstrate tight-junction proteins residing within autophagosomes, yet the sizes of these structures (2.5 – 3 um) are significantly larger than either yeast or mammalian autophagosomes (~200 – 500 nm). The gold standard for cargo residence within an autophagosome is to use electron microscopy to visualize the double membraned structure surrounding the cargo.
• Figure 4I-M: These autophagy proteins are not unique to macroautophagy. The "conjugation of Atg8 to single membranes" a.k.a. "CASM" pathway, which is a host response to damaged endolysosomal membranes, also involves the same autophagy proteins (with exception of Atg6/Beclin-1, whose changes in protein levels may be within range of changes in Gapdh).
• Figure 5L: The multiplicity of infection as well as the Listeria genotype used in this experiment must be included. Drastic positivity for LC3 is unique to Listeria mutants lacking three key proteins: ActA, a surface protein required for subversion of the host's actin polymerization machinery for motility and escape from autophagosomes, as well as PlcA and PlcB, which reduce levels of PI3P – an essential lipid for autophagy. (See PMCID: PMC5777066)
• Figure 5M: Given that this experiment utilized cell lines, use of inhibitors for autophagy/lysosomal functon(SAR405/wortmannin/Bafilomycin A1) and measurement of the degradation of autophagy cargo/receptors (ex. p62) ought to be included. This would help the authors conclusively determine whether or not autophagy is induced or rather inhibited (which could also result in the accumulation of LC3-II).

Version 1:

Reviewer comments:

Reviewer #1

(Remarks to the Author)
The authors have addressed my comments.

Reviewer #2

(Remarks to the Author)
The authors added new results addressing some of my points.

Reviewer #4

(Remarks to the Author)
Summary:
This study examines the existence and role of two distinct isoforms of the microtubule-associated Dbl-family protein ARHGEF2 (Arhgef2 in mouse) and demonstrates that a longer isoform (-219, human/-207, mouse) is preferentially expressed in intestinal epithelial cells while a shorter isoform with a distinct N-terminus (-201) is preferentially expressed in cancer cell lines and mouse bone marrow-derived macrophages. The authors demonstrate that the longer isoform localizes to adherens junctions and can interact with actin via its unique N-terminus. While depletion of the long isoform results in an increase in abundance of the shorter isoform, epithelial cells lacking the long isoform have compromised barrier function suggesting non-redundant roles of the isoforms. Depletion of the long isoform was also associated with upregulation of autophagic pathways in a manner dependent on PI3K and VPS34. The authors demonstrate that 207KO mouse is more susceptible to infection by the intracellular pathogen Listeria monocytogenes, suggesting a protective role of the long isoform. However, experiments in human intestinal organoids show that L. monocytogenes infection is associated with reduced expression of the long isoform and upregulation of the shorter isoform, potentially suggesting that the pathogen co-opts isoform switching to evade ARHGEF2-mediated host defense. The overall model of whether this is a protective

response or a pathogen-driven response is unclear. Association of the shorter ARHGEF2 isoform with LC3-II and STING during L. monocytogenes infection points to its role in driving an autophagic response. Overexpression studies further provide evidence that this ARHGEF2-driven autophagic program is both STING- and ROCK-linked and represents a secondary host defense when the long isoform is depleted.
Overall, this was thorough study that addresses an interesting mechanism of intestinal barrier maintenance and host defense. While a lot of work went into this manuscript and I am overall in favor of publication, there are still several instances where sufficient information was lacking in the text or figure legends to properly evaluate the findings, particularly with respect to L. monocytogenes experiments, and/or inconsistencies existed in the data that were not properly addressed. Finally, several typos and inconsistent explanation of the mouse models made it difficult to follow the manuscript.

Major comments:
With respect to use of L. monocytogenes as a model invasive pathogen, there are key methodological details missing that affect interpretation of the data. The most pressing is, did the authors treat cells and organoids with gentamicin after infection to kill extracellular bacteria? This is routinely done in the field to synchronize infection and prevent unchecked outgrowth of bacteria in the cell culture medium (washing is not sufficient to accomplish this). The images in Fig. 5H show a large number of non-cell-associated L. monocytogenes that would not be expected at 4 hours post-infection, suggesting to me that gentamicin treatment was not done. In this panel, some L. monocytogenes cells co-localize with ARHGEF2-201 and the authors conclude this is happening intracellularly. Without gentamicin treatment, however, live extracellular bacteria could be interacting with the surface of the cells via internalin A, and this co-localization could reflect InlA binding-mediated redistribution of ARHGEF2. Second, with respect to organoid infections, the presence of (multiplying) extracellular bacteria over 18 hours could stimulate extracellular pattern recognition receptors and influence induction of the immune-related factors assayed in Fig. 5B-E.
There are multiple instances where inconsistencies between or within panels are not properly addressed and/or statements made in the text do not match the data that is presented within the accompanying figure (detailed below). This calls into question the reproducibility of some of the findings, especially given that not all immunoblot panels have been quantified and are often representative of n=2 experiments. Further, some conclusions may inaccurately represent the data. While some attempts have been made to quantify immunoblot images in Figure 2, the rigor in other figures is lacking.
Figure 1B, ARHGEF2-219 signal as detected with the common antibody varies greatly between samples (yet is highly reproducible when the isoform-specific -219 antibody is used). A lower band becomes visible in lane two, but it is not clear if this is ARHGEF2-201 or not. Could this suggest that there are multiple different isoforms expressed in intestinal epithelial cells that share the same N-terminus as -219?
Figure 1E lane 1 and Figure 1F are both labeled as IECs (and referred to as such in text), but the figure legend states mouse intestinal organoids for both panels, so it is not clear which model was used. Assuming lane 1 in both panels represents Arhgef2 expression in the same cell type from wild-type animals it is not clear why there is essentially no arhgef2-201 signal in Panel E lane 1, but substantial -201 signal in F lane 1.
There is inconsistency between Figure 5B which shows that ARHGEF2-201 is almost absent at T=0 then increased by T=4 and Figure 6D where ARHGEF2-201 signal is the same at T=0 and T=8. This calls into question whether the shorter isoform is actually reproducibly induced in response to infection. Please comment.
Line 321: reduced LC3-I not LC3-II?
Line 330: “dose-dependent reduction in LC3-II…” is inaccurate. LC3-I is clearly reduced with Y-27632 treatment, but there actually appears to be a slight increase in LC3-II with 10uM Y-27632 relative to untreated.

Minor comments:
-A more clear explanation of how the 207 (fl/fl, vil, KO) mouse models were constructed and more consistent textual references to these models (in the text and figures) would make these experiments easier to understand. For example, "207vil" is not defined in the paragraph describing the mouse models (lines 72-86), but only in the next results section (line 93).
-Two different antibodies against the common C terminus of ARHGEF2 are indicated in Figure 1A, and referenced in the text, but it is not clear why, nor indicated in any subsequent panel which of the two was used. Further, it is not obvious whether an isoform-specific or common antibody was used in each immunoblot image aside from the presence of one or more bands and is in some cases difficult to determine which bands correspond to which isoform (e.g. Fig 1B lane 2; Fig. 1E lane 3). Consider adding arrows to make it more clear which bands correspond to which isoform and adding information about the specific antibody used in each part of the panel to the figure legend or elsewhere.
-The language used in lines 109-112 is redundant and somewhat confusing.
-The figure call out in line 119 is incorrect and should only read Fig. 2M. Fig. 2L is densitometry associated with 2J and should be referenced accordingly.
-Figure legend for Figure 2 states that a One-way ANOVA was used to assess significance and that data are presented as mean +/- SEM (Line 581). Given that only two groups are being compared in each panel, a T-test would be more appropriate. Additionally, assuming that the data points shown represent individual values (as opposed to average values across multiple replicates), standard deviation would be more appropriate.
Line 129-Line 132: conclusions are overstated. There is no evidence presented for differential susceptibility to pathogens at this point.

Comments related to Figure 3:
-Panel D, please comment on the presence of NECTIN-3 upon IP of Arhgef2 in the 207KO strain. Was AFADIN detected under these conditions as well?
-Line 145: Figure call out read: 3A, 3B, S2A, S2B
-Line 148: language and subsequent interpretation of the figure is misleading as both isoforms should be detected with the indicated antibody.

-Panels F and K: Please clarify in the figure legend or elsewhere how 219N was detected (antibody to RFP or ARHGEF2-specfic antibody). Do ARPE-19 cells express the long ARHGEF2 isoform? It seems possible that AFADIN, NECTIN-3 and ACTIN could actually be co-precipitating with the full-length native version of ARHGEF2. Please comment.
Was any AlphaFold modeling (or mutagenesis) done with the full-length ARHGEF2? It seems plausible that conformation of the N-terminus could be impacted by the presence of the rest of the protein.
-Panel M: labels on the right are misaligned.
The experiments done in Figure 3K-M are an interesting addition to the story pointing to a role for the unique N-terminus of the long isoform, but this could have been strengthened by localization studies addressing how disruption of the interaction with ACTG1 impacts long-isoform localization within epithelial cells.
Line 209-211: missing an open parenthesis

Comments related to Figure 4:
-Panel B: please define in the figure legend or key what the red/blue colors indicate.
-Panel C: P70S6k is referred to as simply S6 kinase in the text (line 218), please add parenthetical reference to the shorthand name.
-Panel K: There is no indication of the length of TAM treatment prior to Bafilomycin treatment.

Comments related to Figure 5:
-Panel A: Does not provide any useful information in its current form. Was there some rationale for choosing these specific time points that can be provided?
-Line 299: "…-201 was redistributed to compartments surrounding intracellular bacteria". How do we know that the Listeria cells shown in the image are within compartments? L. monocytogenes would canonically be expected to be replicating within the cytosol by 4hpi. Do the authors have a reason to suspect L. monocytogenes is in a compartment?
-Expression of the human ARHGEF2 isoforms is demonstrated to be modulated by L. monocytogenes infection, but in Panel I the mouse model is used to show that the long isoform is protective from L. monocytogenes infection. Data addressing whether the mouse long isoform is also downregulated during infection with concomitant increase in the short isoform (as in Panel B) or alternatively, assessment of the role of the long isoform in protecting human epithelial cells from infection (via measuring intracellular bacterial burdens or plaquing) would greatly strengthen the figure.
-Line 304: There is no evidence provided that "Targeting of junctional complexes by L. monocytogenes" causes the ARHGEF2 isoform switch, just that the switch occurs during infection. Please soften the language used here.

Comments related to Figure 6:
-The methods indicate that a GFP-expressing L. monocytogenes strain was used (acquired from Microbiologics, Cat #01249UV-V) and no other strain is mentioned. Yet, in panel A GFP-LC3 is used to demonstrate colocalization with L. monocytogenes during infection. Please comment on this discrepancy.
-The conclusion about colocalization of LC3 and -201 on "L. monocytogenes-containing vesicles" (Line 308) appears to be made based on one ROI. Are there additional images and/or quantification that can address (1) the frequency of LC3 association with L. monocytogenes in these infection conditions and (2) the frequency of -201 and LC3-association across multiple infected cells. It seems surprising that LC3 appears to be so strongly associated with the Listeria cells given that they are largely thought to evade autophagy (~10% of L. monocytogenes co-localize with LC3 in macrophage cell lines, for example, but this single ROI suggests a much larger proportion of L. monocytogenes are associated with LC3 in these Caco-2 derivatives). Could this potentially be a factor of what is presumably ARHGEF2 overexpression in this context given that Caco2 cells naturally express more -201 (figure 1C) and are also ectopically expressing 201-RFP? The authors should show data for LC3 association with L. monocytogenes from non-overexpressing Caco-2 cells as controls.

The addition of a graphical model summarizing the role of the two separate ARHGEF2 isoforms under "native" conditions and during infection including the proposed localization/proteins complexes would provide additional clarity on the overall proposed model of the isoform switching mechanism.

Version 2:

Reviewer comments:

Reviewer #4

(Remarks to the Author)
My major concerns have been addressed.

Open Access This Peer Review File is licensed under a Creative Commons Attribution 4.0 International License, which permits use, sharing, adaptation, distribution and reproduction in any medium or format, as long as you give appropriate credit to the original author(s) and the source, provide a link to the Creative Commons license, and indicate if changes were made.
In cases where reviewers are anonymous, credit should be given to 'Anonymous Referee' and the source.
The images or other third party material in this Peer Review File are included in the article's Creative Commons license, unless indicated otherwise in a credit line to the material. If material is not included in the article's Creative Commons license and your intended use is not permitted by statutory regulation or exceeds the permitted use, you will need to obtain permission directly from the copyright holder.
To view a copy of this license, visit https://creativecommons.org/licenses/by/4.0/

DETAILED RESPONSE TO THE REVIEWER COMMENTS

We thank the reviewer for the thoughtful critique and constructive suggestions, which prompted additional experiments and analyses that strengthened the manuscript. Major advances since the initial submission include identification and mutational validation of a bipartite actin-binding motif within the unique N terminus of the long ARHGEF2 isoform. These findings establish that this ARHGEF2 isoform can interact with actin in epithelial junctions as well as microtubules. We further show, using organoids and cell lines, that Arhgef2-201 engages autophagy machinery through a VPS34-sensitive pathway that intersects with STING signaling. The dependence of LC3 lipidation on class III PI3K activity, together with its enhancement by lysosomal inhibition, supports recruitment of canonical macroautophagic components. ARHGEF2 appears to activate a VPS34-dependent membrane-trafficking program that couples autophagy proteins to innate immune signaling. We included new data analyzing DSS colitis and Listeria infections in the absence of the long ARHGEf2 isoform. Please find our detailed responses below.

REVIEWER COMMENTS

Reviewer #1 (Remarks to the Author):

Summary:
The authors examine the role of the two major isoforms of ARHGEF2 (GEF-H1) through the use of specific antibodies, expression constructs and the generation of conditional knockout mice. Their findings indicate that the N-terminus of the long isoform functions as a structural interface between Afadin-Nectin complexes and the cytoskeletal network in intestinal epithelial cells. Loss of the long form of Arhgef2 in mice causes epithelial cell dysfunction, loss of barrier integrity, compensatory inflammation. Use of organoids from these mice reveals that epithelial cell phenotypes are intrinsic and do not require microbial triggers. Finally, authors show that infection of epithelial cells by an invasive pathogen (Listeria) is sufficient to cause changes in isoform expression that appear to contribute to loss of epithelial junction integrity.

This is a well designed study that has important implications for our understanding of how gene splicing and Rho regulation can impact epithelial junctions. The writing of the paper and presentation of the data could be improved in some areas (see minor comments below) but I am generally in support of publication.

Minor comments:
-nomenclature is challenging throughout this paper. Recommend dropping use of GEF-H1 after introduction.

Answer: We follow this recommendation

-line 75, should be “ARHGEF2”, remove dash to be consistent

-line 78 a bit clunky, propose changing to “(Fig.1B), which encodes a unique…”

Answer: corrected

-Figure 1A does a good job of showing isoforms and domains. But the gray bars are not super obvious… maybe add a schematic of an antibody (also in gray) indicating that there are 4 different antibody epitopes (in different species) being depicted? For the other panels, can you indicate species to make it super clear what you are looking at? A label of the gene name at the top and numbering of amino acids would also be helpful.

Answer: the figure was revised

-line 125, Fig.2I is cited twice.
-line 130 a bit clunky, merge into one paragraph?
-Figure 2D,I,L, where are the X-axis labels?-Figure 3A vs 3G. For clarity, recommend indicating antibody used since NT epitope (3A) specific to -207 isoform but CT common epitope (3G) is not.

Answer: corrected

-Figure 3B/H, units are required for the graphs. Y-axis needs to be clarified… nm from what? Why measure vertical distance in B but horizontal in H?

-Figure 3L,M,N. Where is the RFP only control band? If running at a different MW then crop that portion of the gel for display.

-Figure 3K-N. Conserved motifs in K are highlighted but not tested in co-ip experiments, so their role remains unknown. Region of ARHGEF2 used for NT co-ip is not described in results or figure legend… which aa?

Answer: we have added additional figures and corrected omissions.

- Listeria should be italicised. Be consistent with nomenclature.

Answer: Corrected

-Figure 4K, where are X-axis labels?

Answer: we added labels directly instead of referring to bar color in the legends.

-line 288. RFP-tagged ARHGEF2-201 and LC3-GFP are shown to be colocalized with Listeria in Figure 5L. This is confusing since many papers show that Listeria can evade autophagy during infection. Are these bacteria trapped within vacuoles or a subset of total bacteria in the infected cells?

Answer: We have added additional figures showing ARHGEF2-201 localization during Listeria infection. Only a subset of Listeria will be in autophagosome at any given time. We focused on high magnification images that allow us to demonstrate the colocalization of ARHGEF2 and LC3.

-line 299, "linking junctional targeting by L. monocytogenes to innate immune activation". It is not clear that the pathogen is "targeting" expression of Arhgef2. For example, there are no virulence factors shown here to control changes in isoform expression. It seems plausible that a host immune response is causing changes in isoform expression. Recommend changing conclusive statements about what is happening vs how. Same comment for line 327.

Answer: We have removed these speculative statements.

-line 343. "ARHGEF2 isoform regulation may represent a critical determinant of epithelial resistance". Are there any conditions that promote expression of the long isoform over the shorter isoform e.g. in development, repair pathway, etc. ?

Answer: This is a very important question and subject of future studies. The lack of the long isoform in intestinal tumor derived organoids also suggests a role in epithelial differentiation and detailed studies to define the role of ARHGEF2 isoforms in human cancer is an important future goal. We have added this point to the discussion.

Reviewer #2 (Remarks to the Author):

This manuscript investigated the long and short isoforms of the guanine nucleotide exchange factor GEF-H1 in human organoids and mouse intestinal tissues. These are microtubule-associated molecular switch that activates Rho GTPases. The team developed antibodies that can distinguish these isoforms. They make conditional mouse knockouts for the long form Arhgef2-207, which corresponds to the human ARHGEF2-219 isoform. They show that this long form localizes to adherens junctions in polarized intestinal epithelial cells. Deleting Arhgef2-207 increased autophagy activity, increased gut permeability, elevated inflammatory cytokines, accompanied by an elevation in the short form Arhgef2-201. Through a number of Western blot and co-IP analysis, the authors show the short form interacts with STING and LC3. To show the

relevance to human biology, they showed that organoids that are infected with Listeria monocytogenes showed reduced ARHGEF2-219 isoform but increased ARHGEF2-201, which they believe to trigger autophagy. The efforts on the biochemical characterization of binding partners of these isoforms are appreciated. There are some major issues with the experimental control, conclusion, interpretation, and quantification.

Major concerns:

1. Fig. 5 nicely showed that Listeria monocytogenes targeted the longer form while increasing the short form in biochemistry assay. Surprisingly, the manuscript ended without further testing if the conditional knockout mice would have an exacerbated inflammation by Listeria monocytogenes infection. The entire story stopped short by describing a changed abundance of an isoform that binds to different partners.

Answer: Deletion of Arhgef2-207 in the epithelium increases the susceptibility of mice to *Listeria* infection significantly. We have added this data. We also include the identification and mutational validation of a bipartite actin-binding motif within the unique N terminus of the long ARHGEF2 isoform.

2. There are lots of Western blots or IP results in this manuscript, but they did not seem to be quantified. This raises major concerns especially the authors used these results for their major conclusions.

Answer: We have quantified the expression of mRNA and protein expression thoughout the manuscript. Western blots were carried with at least two independent samples in parallel. We have included these additional samples in the figures. We also added additional quantitation of gene and protein expressions. Conclusions are supported by multiple lines of investigation using isolated primary IECs, mouse and human organoids, and cell lines.

3. Some of the conclusions are overstated. For example, showing elevated cytokine levels by qPCR or RNA-seq may not be equivalent with spontaneous inflammation or an increased susceptibility of colitis. Whether these KO mice have increased colitis susceptibility has not been tested here.

Answer: We have included new experiments showing the enhanced susceptibility of Arhgef2-207 KO mice to DSS colitis and to Listeria infection.

4. The statement such as “loss of Arhgef2-207 reprograms IECs toward an autophagy dominant stress” shall be documented by stronger results.

Answer: We have added a more specific analysis using inhibitors of the autophagic gene regulation in intestinal organoids, in response to *Listeria* and expression of ARHGEF2 in intestinal epithelial cells. Together, these data indicate that ARHGEF2-201 engages a RhoA–ROCK- and STING-linked, VPS34-dependent autophagy program, consistent with recruitment of autophagy machinery in a membrane-trafficking and innate immune context.

Detailed comments:
Fig. 1B-C. Not clear why some were labeled as ARHGEF2 others were Arhgef2. If they refer to the antibodies used for the Western blot, the label should be modified.

Answer: We use ARHGEF2-219 and ARHGEF2-201 for the human protein and Arhgef2-207 and Arhgef2-201 to identify the mouse proteins to avoid misunderstandings. We correct the mistakes in Figure1B and C.

Fig. C. "Importantly, ARHGEF2-219 expression was absent in human colorectal adenocarcinoma-derived cell lines Caco2, T-84, HCT116 and HT-29 that expressed the shorter 84 ARHGEF2-201 isoform instead (Fig 1C)." --- This conclusion was based on an antibody raised against the murine Arhgef2(656-1000). Why not use the ARHGEF2 antibody here?

Answer: We have added the specific western blot for ARHGEF2-219.

"These mice maintained Arhgef2-207 expression in IECs but lacked shorter GEF-H1 isoforms in both IECs and macrophages (Fig. 1D)," ---- Here should be referring to Fig. 1E.
Fig. 1F, "ARHGEF2-219 was 97 detected in and adjacent to Nectin-Afadin complexes and in puncta throughout the cytoplasm of 98 IECs (Fig.1F)." ---- This is difficult to appreciate from the images.

Answer: We have added new figures and corrected labeling mistakes.

"these findings reveal that differentiated intestinal epithelial cells express long ARHGEF2 isoforms that are lost in intestinal cancer cell lines" ---- Can the second part of this statement be supported by staining in monolayer of cancer cell lines?

Answer: Western blot analysis is the most sensitive approach to identify short and long ARHGEF2 isoforms in epithelial cells.

Fig. 2A. The diagram includes three separate alleles can be confusing to readers.

Fig. 2D. Would be helpful to know where the primers are located, according to the diagram in Fig. 1A.

Fig. 2G. The label of TAM for one of the groups can be confusing, assuming the Arhgef2-207cko mice were also administrated with TAM.
Fig. 2J, 2K, 2M. “Deletion of Arhgef2-207 further caused the loss of the Na^{+}/K^{+}-ATPase subunits ATP1A1 and ATP1B1” ---- Normally, if you claim a loss of something, the blots should be quantified from multiple animals and experiments. Same comments for Fig. 2K and 2M.

Answer: We have revised these figures for clarity. We have included the quantification for ATPase, STING, REG3B and added duplicate sample assessments.

Line 139: “Genetic ablation of Arhgef2-207 induces compensatory upregulation of Arhgef2-201, which is insufficient to restore barrier integrity, leading to spontaneous small intestinal inflammation and heightened susceptibility to colitis.” ---- This was overstated, as neither spontaneous inflammation nor colitis was seen at this point.

Answer: MLNs and lamina propria demonstrate enhanced immune responses and increased expression of inflammatory cytokines after deletion of Arhgef2-207. We have included new data demonstrating the enhanced susceptibility to DSS colitis with induction of inflammatory cytokine expression in MLNs upon deletion of Arhgef2-207 and induction of Arhgef2-201.

Fig. 3C. “The AJ consists of two concentric rings: an outer ring containing Nectin-Afadin complexes and an inner ring composed of E-cadherin–catenin assemblies” --- How is Arhgef2-207 relative to the inner ring?

Answer: We have clarified this statement. ARHGEF2-207 localizes to the Nectin-Afadin complexes that are apical to the E-cadherin assemblies.

Fig. 3I-J: The labels on the top of these blots are the same, and it was confusing what is different between the two? Since Arhgef2-201 is also expressed in wild type cells (Fig. 3G), does ZO1 also interact with Arhgef2-201 in wild type cells? It is not clear about the significance of I and J here, especially no quantitative comparison was made between the KO and WT.

Answer: We have corrected the labels in these figures.

“Arhgef2-201 is enriched at tight junctions (TJs) in association with ZO-1.” --- This does not change from WT to 207 KO.

Answer: Yes, we found Arhgef2-201 associated with TJs before and after deletion of 207. However, upon 207 deletion ZO-1 also enriched in cytoplasmic aggregates in IECs.

Fig. 4E. “IECs from Arhgef2 207cKO mice formed enlarged intracellular compartments enriched

for the tight junction protein ZO-1, which were absent in Arhgef2207vilc controls (Fig. 4E). ---- This statement is difficult to appreciate.

Answer: We have included new figures to address this.

Fig. 4F, G: "These compartments colocalized with LAMP-1 and LC3, consistent with their identity as autophagosomes (Fig. 4F, G)." ---- Including the control here seems important.

Answer: We include the requested controls

Reviewer #3 (Remarks to the Author):

OVERALL ASSESSMENT:
In the submitted manuscript, the authors make the argument that the mouse/human protein Arhgef2/ARHGEF2 possesses two isoforms of differing lengths that interact with distinct sets of cell-junction proteins in intestinal epithelia; thus, affecting the integrity of the intestinal barrier. The authors argue that the N-terminal region of Arhgef2/ARHGEF2, present only in the long isoform, is responsible for these distinct interactions. Given the significant phenotypes associated with preferential expression of the short isoform, the authors performed transcriptomics in intestinal epithelial cells producing each isoform and identified several stress-response and degradative pathways – one of which being autophagy – enriched in cells only expressing the short isoform. The authors provide several pieces of data to demonstrate that markers for innate immune and autophagy responses increase after the switch in expression to the short isoform, consistent with their transcriptomics data. Lastly, the authors suggest that Listeria infection increases Pol II occupancy at the promoter site for the short isoform, which upon expression, interacts with autophagy-regulating protein, STING, as well as the autophagy protein, LC3. Altogether, the data presented suggest that bacterial infection alters the expression of Arhgef2/ARHGEF2 from a long to a short isoform, with the latter lacking interaction domains with the appropriate cell-junction proteins required for the maintenance of barrier integrity, causing downstream effects such as the induction of innate immune and autophagic responses. While the authors have produced and characterized a mouse model that affects the activity of cell-junctional proteins that could benefit the field, additional pieces of data and controls are required to support several of their claims in this report.

MAJOR COMMENTS:
1. The writing of the text as well as the readability of the figures contain room for improvement. There are multiple grammatical errors throughout, references to the wrong figure panels or references to data that doesn't exist in the figure suggesting a lack of rigor in preparation of the manuscript.

Answer: We have corrected these oversights

In addition to these concerns, a simplified nomenclature for the long vs short isoforms of ARHGEF2 would significantly improve the flow of the manuscript and interpretation of the data in the figures, allowing readers to more efficiently identify the sample without relying on identifiers in a smaller superscript.

Answer: Throughout the text we use ARHGEF2-201 and ARHGEF2-219 for the human isoforms and Arhgef2-201 and Arhgef-207 for the mouse isoforms. We have abbreviated the designation of the used mouse strains to improve clarity in figures and text.

Additionally more minor recommendations for improved readability of figures include among other things:

a. Fig. 1BC – explicitly direct the reader to the relevant bands of interest – higher and molecular weight ARHGEF – using arrows

Answer: We have better highlighted the specific antibodies used to detect the mouse and human short and long isoforms in Figure 1A. We have directly labeled the isoforms in the western blots. Specific antibodies were developed to detect the N-termini of human and mouse long isoforms. To detect the short isoform we used antibodies originally raised against the ARHGEF2-201. These antibodies can detect shared C terminal regions in both isoforms.

b. Fig. 2DFIL – label each color in the graph/axes instead of relying on the figure legend
c. Fig. 3BH – label each color in the graph/axes instead of relying on the figure legend
d. Fig. 4K – label each color for each bar in the graph.

Answer: We have added this labels

2. The methodology section and some figure legends are severely lacking in detail in some cases making the data impossible to interpret.

Some examples include how the viability of organoids was measured, details regarding Listeria infections (strain identity, MOI);

timing of serum FITC-dextran quantification (collection of serum on different dates or Cre-induction was staggered to collect serum on the same date, etc.);

depletion laser and power settings for STED microscopy and how lateral images were obtained and many many others.

Answer: We have added this detailed information.

While these may seem like small issues they are in fact important issues. For example, it is known that Listeria has multiple mechanisms for evading autophagy including the use of actin based motility and phospholipases, did the authors use strains lacking these factors to see association with autophagy and if not how do they explain the discrepancy with the literature and how these bacteria were targeted by autophagy?

Answer: The detailed analysis of Listeria effectors that target ARHGEF2 is an important future goal but beyond the scope of this initial report. This detailed analysis may show unexpected mechanisms. We do not see a discrepancy with the literature concerning Listeria and autophagy as most data is derived from macrophages, cancer cell lines or modified mouse models to facilitate infections.

Listeria doubles every 40 minutes within cells, how long were the organoids infected for and at what MOI, this is critical for considering whether phenotypes observed were due to overwhelming bacterial infection or if this was early during infection and likely a response to initial detection by pattern recognition receptors. These are just some examples of how the data is uninterpretable with no details provided at all about the experimental approaches and controls.

Answer: *Listeria monocytogenes* serotype 1/2a (FDA strain LS808; Microbiologics, cat# 01249UV-V) was freshly prepared at 5 × 10^7 CFU/mL. For infection, 10 µL of bacterial suspension (5 × 10^5 CFU) was added to each well of a 24-well plate containing approximately 100 cultured gut epithelial organoids. Organoids were washed after 2 hours of infection and harvested at 0, 4, 6, 8, and 18 h post-infection for RNA isolation or immunoprecipitation/western blot analysis.

3. A reference control (ex. for another protein that regulates cell-junction integrity) to assess the effect sizes of various phenotypes (ex. losses in barrier integrity Figures 2) as well as the specificity for ARHGEF2 in the induction of autophagy and mucosal innate immunity (Figs. 4 and 5) should be included for key experiments.

Answer: We assess multiple junction associated proteins. The phenotype was reproduced in primary IEC isolated from mice, in mouse as well as human organoids.

The authors state that these processes are regulated by ARHGEF2 isoforms but it seems more likely to me given that 10% of the genome is differentially regulated in their mutants that all of these downstream effects are caused by the loss of junction integrity and barrier function leading to stress responses. There are no other controls for junction dysfunction so it is impossible to know if everything observed is simply a pleiotropic effect of loss of junction function. There is

also no justification provided for looking at autophagy and cell death pathways over any of the other ~2000 genes differentially expressed.

Answer: Gene set enrichments of the significantly regulated genes identified autophagy pathways. The regulation of autophagy related genes was confirmed by immunostaining and protein analysis in primary IECs and mouse organoids. We further now show, through genetic upregulation or expression that Arhgef-201 engages autophagy machinery through a VPS34-sensitive pathway that intersects with STING signaling.

4. Evidence of absence for the opposing markers and isoforms are required to support the claims for isoform-specific compartmentalization of ARHGEF2 and their regulation of autophagy and innate immunity. Thus, all of the immunoprecipitation experiments should include blots for both isoforms (as the authors have shown for Figure 3I). Similarly, additional blots for the excluded junctional complex (ex. include an AFADIN/NECTIN-3 blot in Figure 3I or a ZO-1 blot in Figure 3D) are also required.

Answer: We have carried out IPs with newly generated ABs against mouse and human Arhgef2 long isoforms. These ABs are possible as the long isoforms have distinct N termini of 221 and 163AA respectively. However, as long and short isoforms share a common C terminus the other antibodies available detect both long and short isoforms.

5. The authors repeatedly reference “specific” or “selective” changes caused by Listeria, yet the authors have not infected with other bacteria such as Salmonella or simulated infection using Internalin-deficient Listeria, attenuated Listeria (for example lacking LLO), heat-killed bacteria, or PAMPs. This figure is severely underdeveloped and overinterpreted without significant other controls to determine how Listeria or infection more generally might alter isoform expression.

Answer: We have corrected these statements

6. While the idea that the switch from the long to short isoform of ARHGEF2 results in the activation of autophagy, the authors ought to consider investigating mTOR activity and lysosomal integrity in greater detail as the phenotypes described in Figures 4 and 5 can all be described by perturbations in these pathways/organelles rather than a specific effect on autophagy. Thus, at their present state, these final two figures do not significantly contribute to the story.

Answer: Following the recommendations of the reviewer we now have included a more detailed analysis of the autophagic response in organoids and cell lines upon upregulation of Arhgef2-201 and in intestinal epithelial cell upon overexpression of ARHGEF2-201.

Our new data are consistent with an engagement of the autophagy machinery by ARHGEF2 through a VPS34-sensitive pathway that intersects STING signaling. The dependence of LC3 lipidation on class III PI3K activity, together with its enhancement by lysosomal inhibition, supports recruitment of canonical macroautophagy. ARHGEF2 activates a RhoA and STING mediated autophagic signaling axis that couples autophagy proteins to innate immune responses.

MINOR COMMENTS:
• Line 115: Figure call-out refers to 1D when it should be referring to Figure 2D.
• Line 178: This call-out regarding the expression of the RFP fusion constructs in the ARPE-19 cell line was probably meant to reference Figure 3L.

Answer: we corrected these mistakes

• Line 198: In the autophagy field, the use of the word "core" when describing autophagy genes and proteins is restricted to proteins that are absolutely required for autophagosome biogenesis. Foxo3 and Hif1a are regulatory proteins and are not typically described as core-autophagy genes. The authors also overlook Sqstm1, also called p62, in Fig. 4B. While not necessarily a "core" autophagy protein, Sqstm1 is a ubiquitously discussed protein in autophagy manuscripts.

Answer: We have corrected the text. We identified the signature through gene set enrichment analysis of the significantly regulated genes. The list shown represents genes that a part of GO:0006914 - autophagy, GO:0016236- macroautophagy, GO:0010506 - regulation of autophagy. We now only highlight genes with a published role in autophagy or that regulated autophagy with references provided.

• Line 283: Figure call-out likely refers to Figures 5H and 5I instead of 5G and 5J.

• Figure 1E is missing a call-out in the text.

Answer: We corrected these mistakes

• Figure 2H may be better organized by switching the order of the mouse genotypes to be consistent with all other graphs in this figure.

Answer: We have done so

• Figure 3L-N ought to include a mutagenized version of the named structural motifs fused to RFP to demonstrate the importance of those motifs for interaction with afadin and Nectin-3. The authors use expression of this fragment of the protein to argue that these domains are important but did not do any mutagenesis of any of the domains to demonstrate that any of them or all of

them are in fact important. Given that this figure is all done with expressed fusion proteins this is incredibly straightforward and simple to do.

Answer: We now include the alphafold supported identification and mutational analysis of a bipartite motif in the N-terminus of 207 that mediate binding to ACTIN. ARHGEF2-219 engages ACTG1 through a bipartite interface that requires cooperative contributions from both the RHSWGP (AA 26-31) motif and the EALFLQIREGLDTFLGLQHQG (AA 51-71) region, with disruption of either element abolishing actin binding.

• Figure 4FG are referenced in the discussion (Line 317) to demonstrate tight-junction proteins residing within autophagosomes, yet the sizes of these structures (2.5 – 3 um) are significantly larger than either yeast or mammalian autophagosomes (~200 – 500 nm). The gold standard for cargo residence within an autophagosome is to use electron microscopy to visualize the double membraned structure surrounding the cargo.

Answer: We have replaced the figure to better show the variation in vesicle size that arise in the phenotyp. LC3 positive vesicles are in the range detectable by confocal microscopy (230nm) but LC3 and ZO-1 are also detected in larger aggregates up to 2-3um. We now demonstrate that the GEF-H1 appears to activate a VPS34-dependent membrane-trafficking program that couples autophagy proteins to innate immune signaling.

• Figure 4I-M: These autophagy proteins are not unique to macroautophagy. The “conjugation of Atg8 to single membranes” a.k.a. “CASM” pathway, which is a host response to damaged endolysosomal membranes, also involves the same autophagy proteins (with exception of Atg6/Beclin-1, whose changes in protein levels may be within range of changes in Gapdh).

Answer: We now demonstrate that the phenotype most likely represents an engagement of the autophagy machinery by ARHGEF2 through a VPS34-sensitive pathway. The dependence of LC3 lipidation on class III PI3K activity, together with its enhancement by lysosomal inhibition, supports recruitment of canonical macroautophagy components rather than a VPS34-independent CASM-like mechanism.

• Figure 5L: The multiplicity of infection as well as the Listeria genotype used in this experiment must be included. Drastic positivity for LC3 is unique to Listeria mutants lacking three key proteins: ActA, a surface protein required for subversion of the host’s actin polymerization machinery for motility and escape from autophagosomes, as well as PlcA and PlcB, which reduce levels of PI3P – an essential lipid for autophagy. (See PMCID: PMC5777066)

Answer: We corrected this omission. The precise analysis of *Listeria* effectors engaging the ARHGEF2 system is an important goal in our future experiments as part of a follow up work.

• Figure 5M: Given that this experiment utilized cell lines, use of inhibitors for autophagy/lysosomal functon (SAR405/wortmannin/Bafilomycin A1) and measurement of the degradation of autophagy cargo/receptors (ex. p62) ought to be included. This would help the authors conclusively determine whether or not autophagy is induced or rather inhibited (which could also result in the accumulation of LC3-II).

Answer: Ee have carried out these experiments on organoids and cell lines. We now demonstrate that the phenotype most likely represents an engagement of the autophagy machinery by ARHGEF2 through a VPS34-sensitive pathway. Overexpression of ARHGEF2-201 increased total STING abundance and phosphorylation and was accompanied by elevated ATG16L1 and p62 levels relative to controls. LC3-I abundance was increased, whereas LC3-II was most evident following lysosomal inhibition with bafilomycin A1, indicating BafA1-sensitive LC3 turnover. Inhibition of VPS34 with SAR405 or broader PI3K inhibition with wortmannin reduced LC3-II and ATG16L1 levels, while SAR405 induced marked p62 accumulation and reduced STING phosphorylation, consistent with impaired autophagic degradation upon VPS34 inhibition.

We thank the reviewer for the thoughtful critique and constructive suggestions, which prompted additional experiments and analyses that strengthened the manuscript. Please find our detailed responses below.

Reviewer #4 (Remarks to the Author):
Summary:
This study examines the existence and role of two distinct isoforms of the microtubule-associated Dbl-family protein ARHGEF2 (Arhgef2 in mouse) and demonstrates that a longer isoform (-219, human/-207, mouse) is preferentially expressed in intestinal epithelial cells while a shorter isoform with a distinct N-terminus (-201) is preferentially expressed in cancer cell lines and mouse bone marrow-derived macrophages. The authors demonstrate that the longer isoform localizes to adherens junctions and can interact with actin via its unique N-terminus. While depletion of the long isoform results in an increase in abundance of the shorter isoform, epithelial cells lacking the long isoform have compromised barrier function suggesting non-redundant roles of the isoforms. Depletion of the long isoform was also associated with upregulation of autophagic pathways in a manner dependent on PI3K and VPS34. The authors demonstrate that 207KO mouse is more susceptible to infection by the intracellular pathogen Listeria monocytogenes, suggesting a protective role of the long isoform. However, experiments in human intestinal organoids show that L. monocytogenes infection is associated with reduced expression of the long isoform and upregulation of the shorter isoform, potentially suggesting that the pathogen co-opts isoform switching to evade ARHGEF2-mediated host defense.
The overall model of whether this is a protective response or a pathogen-driven response is unclear. Association of the shorter ARHGEF2 isoform with LC3-II and STING during L. monocytogenes infection points to its role in driving an autophagic response.
Overexpression studies further provide evidence that this ARHGEF2-driven autophagic program is both STING- and ROCK-linked and represents a secondary host defense when the long isoform is depleted.
Overall, this was thorough study that addresses an interesting mechanism of intestinal barrier maintenance and host defense. While a lot of work went into this manuscript and I am overall in favor of publication, there are still several instances where sufficient information was lacking in the text or figure legends to properly evaluate the findings, particularly with respect to L. monocytogenes experiments,
and/or inconsistencies existed in the data that were not properly addressed.
Finally, several typos and inconsistent explanation of the mouse models made it difficult to follow the manuscript.

Major comments:
With respect to use of L. monocytogenes as a model invasive pathogen, there are key methodological details missing that affect interpretation of the data. The most pressing is, did the authors treat cells and organoids with gentamicin after infection to kill extracellular bacteria?

This is routinely done in the field to synchronize infection and prevent unchecked outgrowth of bacteria in the cell culture medium (washing is not sufficient to accomplish this).
Answer: These are intestinal organoids and epithelial cell lines not macrophages that actively phagocytose Listeria. We initially did not use gentamycin as we wanted to capture ARHGEF2 regulation at all different stages of cell entry, vaculolar lysis, actin tail assembly and autophagy.

The images in Fig. 5H show a large number of non-cell-associated *L. monocytogenes* that would not be expected at 4 hours post-infection, suggesting to me that gentamicin treatment was not done. In this panel, some L. monocytogenes cells co-localize with ARHGEF2-201 and the authors conclude this is happening intracellularly.
Answer: These are z sections confocal microscopy images. We have added arrows to indicate the level of the xy section represented and to indicate intracellular Listeria surrounded by ARHGEF2-201.

Without gentamicin treatment, however, live extracellular bacteria could be interacting with the surface of the cells via internalin A, and this co-localization could reflect InlA binding-mediated redistribution of ARHGEF2.
Answer: The exact involvement of Listeria effectors in these processes will need to be established. InlA is indeed is a Listeria candidate effector for targeting ARHGEF2 which we will investigate in future experiments.

Second, with respect to organoid infections, the presence of (multiplying) extracellular bacteria over 18 hours could stimulate extracellular pattern recognition receptors and influence induction of the immune-related factors assayed in Fig. 5B-E.
Answer: We focused on TFs and kinases that are part of the ARHGEF2 signaling pathway that is activated by *Listeria* infection and peptidoglycans (PMID: 30902986, PMID: 39984444). We have shown that ARHGEF2-201 is required for peptidoglycan signaling in the activation of NF-kB, IRF5, Stat1 activation. ARHGEF2 is upstream of type I interferon responses during peptidoglycan recognition (PMID: 39984444).

There are multiple instances where inconsistencies between or within panels are not properly addressed and/or statements made in the text do not match the data that is presented within the accompanying figure (detailed below). This calls into question the reproducibility of some of the findings, especially given that not all immunoblot panels have been quantified and are often representative of n=2 experiments.
Answer: We have included additional samples or repeat experiments.

Further, some conclusions may inaccurately represent the data. While some attempts have been made to quantify immunoblot images in Figure 2, the rigor in other figures is lacking. Figure 1B, ARHGEF2-219 signal as detected with the common antibody varies greatly between samples

(yet is highly reproducible when the isoform-specific -219 antibody is used). A lower band becomes visible in lane two, but it is not clear if this is ARHGEF2-201 or not. Could this suggest that there are multiple different isoforms expressed in intestinal epithelial cells that share the same N-terminus as -219?
Answer: Immunoblots for the short form of ARHGEF2 detect a number of bands these may include other isoforms, cleavage products and distinct phosphorylation events. We have thus far found only the 219 and 201 isoforms in IECs by mass spec that identified the specific N-termini of these isoforms **(**Supplementary Data Table 1 and 2**)**. Future experiments will attempt to characterize additional isoforms, cleavage products and phosphorylation events, but that is beyond the current publication.

Figure 1E lane 1 and Figure 1F are both labeled as IECs (and referred to as such in text), but the figure legend states mouse intestinal organoids for both panels, so it is not clear which model was used. Assuming lane 1 in both panels represents Arhgef2 expression in the same cell type from wild-type animals it is not clear why there is essentially no arhgef2-201 signal in Panel E lane 1, but substantial -201 signal in F lane 1.
Answer: Figures 1E and 1F demonstrate that intestinal epithelial cells (IECs) express the long Arhgef2 isoform, whereas bone marrow-derived macrophages express only the short isoform. Both isoforms are transcribed from distinct promoters and unique first exons that encode isoform-specific N termini, but splice into a common set of downstream exons encoding the shared C terminus (Fig. 1A).

The previously generated Arhgef2TM1 gene-trap mice were created before the long isoform was identified. Consequently, the gene-trap cassette was inserted within the exon(s) specific to the short isoform, disrupting its transcription while leaving the promoter and unique first exon of the long isoform intact. As a result, the long Arhgef2 isoform continues to be expressed in IECs through alternative splicing around the gene-trap insertion, whereas expression of the short isoform is abolished (Fig. 1E,F).
Because macrophages express only the short isoform, Arhgef2TM1 mice completely lack Arhgef2 expression in these cells. In contrast, IECs retain expression of the long isoform but lose the short isoform, making the Arhgef2TM1 line an unintended yet highly informative isoform-specific knockout model that selectively eliminates the short Arhgef2 isoform while preserving the long epithelial isoform (Fig. 1F).
There is inconsistency between Figure 5B which shows that ARHGEF2-201 is almost absent at T=0 then increased by T=4 and Figure 6D where ARHGEF2-201 signal is the same at T=0 and T=8. This calls into question whether the shorter isoform is actually reproducibly induced in response to infection. Please comment.
Answer: We have included a new repeat experiment in Figure 6B, D.

Line 330: “dose-dependent reduction in LC3-II…” is inaccurate. LC3-I is clearly reduced with Y-27632 treatment, but there actually appears to be a slight increase in LC3-II with 10uM Y-27632 relative to untreated.
Answer: corrected

Minor comments:
-A more clear explanation of how the 207 (fl/fl, vil, KO) mouse models were constructed and more consistent textual references to these models (in the text and figures) would make these experiments easier to understand. For example, "207vil" is not defined in the paragraph describing the mouse models (lines 72-86), but only in the next results section (line 93).
Answer: Corrected these omissions.

-Two different antibodies against the common C terminus of ARHGEF2 are indicated in Figure 1A, and referenced in the text, but it is not clear why, nor indicated in any subsequent panel which of the two was used.
Answer: Only one was used in the revised manuscript and is indicated in Fig.1A.
Further, it is not obvious whether an isoform-specific or common antibody was used in each immunoblot image aside from the presence of one or more bands and is in some cases difficult to determine which bands correspond to which isoform (e.g. Fig 1B lane 2; Fig. 1E lane 3). We indicated the use of Abs in figure legends.

Consider adding arrows to make it more clear which bands correspond to which isoform and adding information about the specific antibody used in each part of the panel to the figure legend or elsewhere.
Answer: Specific antibodies detecting AA6-28 of human ARHGEF2-219 and AA57-76 of mouse Arhgef2-207 were used to detect the long isoforms. An antibody raised against AA656-1000 of human ARHGEF2 was used to detect human and mouse long and short isoforms.
We had specific ABs in the labels of figures in the previous version of the paper but reviewers suggested to label differently. We have added an explanation in Figure 1 and highlighted this in the method part.

-The language used in lines 109-112 is redundant and somewhat confusing.
Answer: Clarified

-The figure call out in line 119 is incorrect and should only read Fig. 2M. Fig. 2L is densitometry associated with 2J and should be referenced accordingly.
Answer: corrected

-Figure legend for Figure 2 states that a One-way ANOVA was used to assess significance and that data are presented as mean +/- SEM (Line 581). Given that only two groups are being compared in each panel, a T-test would be more appropriate.
Additionally, assuming that the data points shown represent individual values (as opposed to average values across multiple replicates), standard deviation would be more appropriate.
Answer: corrected

Line 129-Line 132: conclusions are overstated. There is no evidence presented for differential susceptibility to pathogens at this point.
Answer: We have corrected this. Figure 6I now shows susceptibility to Listeria of 207KO mice.

Comments related to Figure 3:
-Panel D, please comment on the presence of NECTIN-3 upon IP of Arhgef2 in the 207KO strain. Was AFADIN detected under these conditions as well?
Answer: Yes, we have included the Afadin blot in the figure.

-Line 145: Figure call out read: 3A, 3B, S2A, S2B
Answer: Corrected

-Line 148: language and subsequent interpretation of the figure is misleading as both isoforms should be detected with the indicated antibody.
Answer: The antibody raised against the specific N-terminus of ARHGEF2-207 only detects the long isoform. The common C-terminus AB detects long and short isoforms

-Panels F and K: Please clarify in the figure legend or elsewhere how 219N was detected (antibody to RFP or ARHGEF2-specfic antibody).
Answer: 219N was detected with the N-terminus specific AB 6-28. We have clarified this in the figure legend and in the figure

Do ARPE-19 cells express the long ARHGEF2 isoform?
Answer: ARPE-19 cells do not express isoform 219.

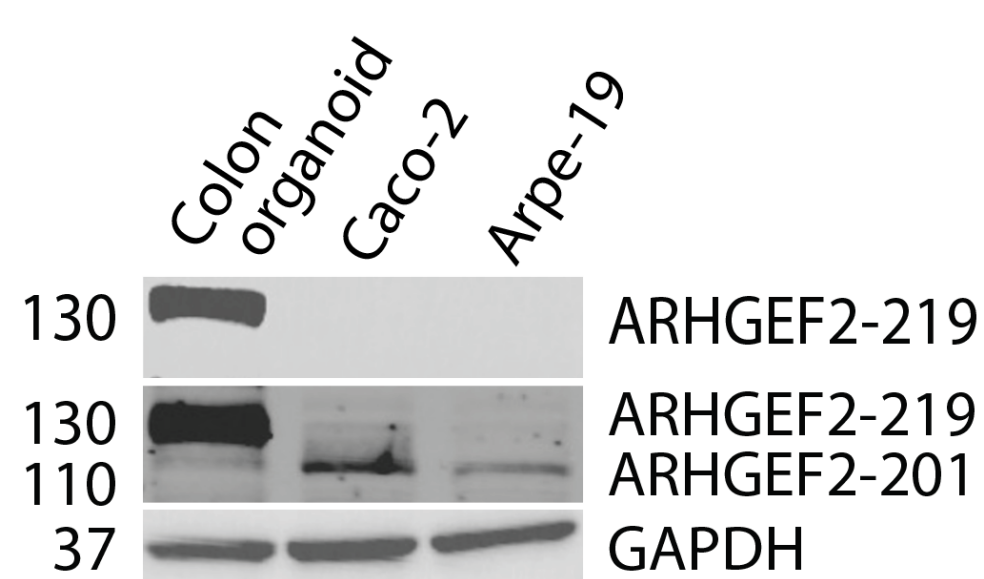

It seems possible that AFADIN, NECTIN-3 and ACTIN could actually be co-precipitating with the full-length native version of ARHGEF2. Please comment.
Answer: As shown in Fig. 3D, immunoprecipitation of the long Arhgef2-207 isoform co-precipitated NECTIN-3, and reciprocal immunoprecipitation with a NECTIN-3 antibody selectively pulled down the long Arhgef2-207 isoform but not the short isoform. These reciprocal co-immunoprecipitation experiments demonstrate a specific association between the long Arhgef2-207 isoform and the NECTIN-3 adhesion complex.

Was any AlphaFold modeling (or mutagenesis) done with the full-length ARHGEF2? It seems plausible that conformation of the N-terminus could be impacted by the presence of the rest of the protein.
Answer: Attempts to model interactions using the full-length ARHGEF2 protein were unsuccessful because AlphaFold was unable to generate reliable structural predictions for a protein of this size in complex with a second binding partner. In addition, the conformation of full-length ARHGEF2 is likely dynamically regulated by post-translational modifications, including phosphorylation and dephosphorylation, which may influence its subcellular localization, intramolecular conformation, and protein-protein interactions. Future studies will define these regulatory mechanisms and determine how they modulate ARHGEF2 structure and function.

-Panel M: labels on the right are misaligned.
Answer: corrected

The experiments done in Figure 3K-M are an interesting addition to the story pointing to a role for the unique N-terminus of the long isoform, but this could have been strengthened by localization studies addressing how disruption of the interaction with ACTG1 impacts long-isoform localization within epithelial cells.
Answer: This would likely require the expression of a reporter fusion protein of ARHGEF207 or ARHGEf2-219 in mice to maintain IEC polarization and natural regulation of the GEF. Over expression experiments with GEFs are exceedingly difficult as a precise localization and regulation is required to avoid ectopic activation of all GTPases. We are working on such reporter systems.
Comments related to Figure 4:
-Panel B: please define in the figure legend or key what the red/blue colors indicate.
Answer: Corrected

-Panel C: P70S6k is referred to as simply S6 kinase in the text (line 218), please add parenthetical reference to the shorthand name.
Answer: corrected

-Panel K: There is no indication of the length of TAM treatment prior to Bafilomycin treatment.
Answer: We have added this information. We used 48 hours after TAM treatment.

Comments related to Figure 5:
-Panel A: Does not provide any useful information in its current form. Was there some rationale for choosing these specific time points that can be provided?
Answer: We have removed this figure. We found that infection of human organoids takes much longer time compared to macrophages or cell lines. These time points were empirically determined.

-Line 299: "…-201 was redistributed to compartments surrounding intracellular bacteria". How do we know that the Listeria cells shown in the image are within compartments?
Answer: Caco-2 cells expressing ARHGEF2-201-RFP were infected with *L. monocytogenes* and imaged over time by three-dimensional (3D) confocal microscopy using sequential x-y-z image acquisition. The z section level was indicated by arrows in Fig. 6A. We revised the language to reflect the findings.

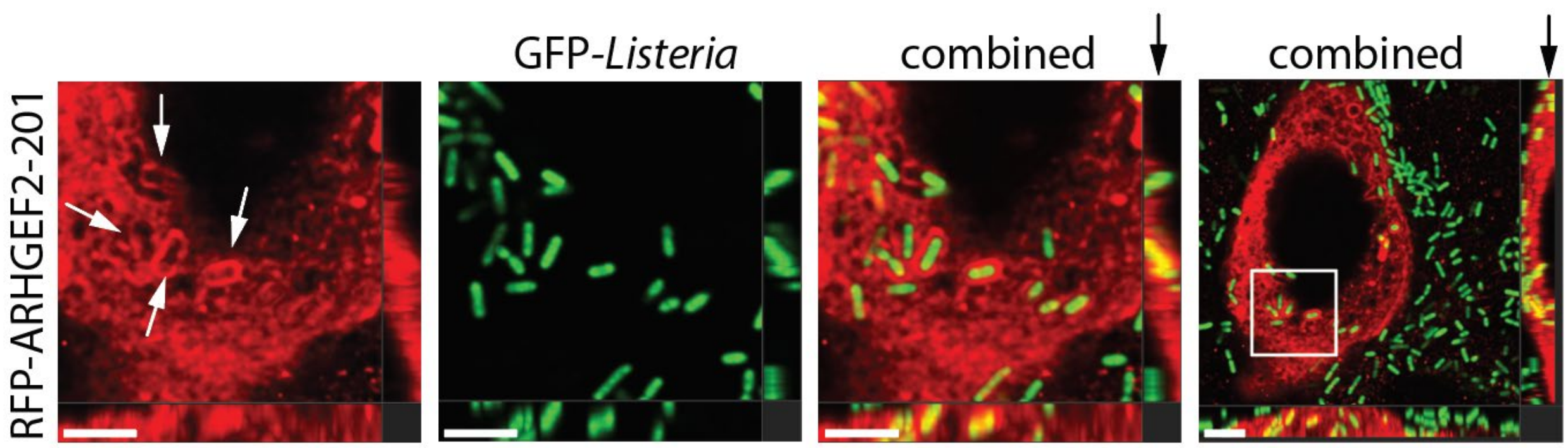

L. monocytogenes would canonically be expected to be replicating within the cytosol by 4hpi. Do the authors have a reason to suspect L. monocytogenes is in a compartment?
Answer: We observed ARHGEF2 associating and surrounding some of the intracellular *Listeria* by 3D confocal microscopy. How ARHGEF2 regulates intracellular lifecycle of Listeria is an important future goal of our investigations. We revised the language to reflect the findings.

-Expression of the human ARHGEF2 isoforms is demonstrated to be modulated by L. monocytogenes infection, but in Panel I the mouse model is used to show that the long isoform is protective from L. monocytogenes infection. Data addressing whether the mouse long isoform is also downregulated during infection with concomitant increase in the short isoform (as in Panel B) or alternatively, assessment of the role of the long isoform in protecting human epithelial cells from infection (via measuring intracellular bacterial burdens or plaquing) would greatly strengthen the figure.

Answer: Figure 5B shows that Listeria reduces the long isoform and can induce the short isoform. Figure 5I shows that genetic deletion of ARHGEF2-207 deletion renders mice susceptible to Listeria infection likely because of altered barrier function of the epithelium. Listeria is a human pathogen that requires human E-cadherin for efficient invasion of IECs. That is why we used human intestinal organoids to study the regulation of the ARHGEF2 isoforms by Listeria. Mechanisms that allow Listeria to invade mouse intestines may be distinct.

-Line 304: There is no evidence provided that "Targeting of junctional complexes by L. monocytogenes" causes the ARHGEF2 isoform switch, just that the switch occurs during infection. Please soften the language used here.
Answer: Agreed, corrected the language.

Comments related to Figure 6:
-The methods indicate that a GFP-expressing L. monocytogenes strain was used (acquired from Microbiologics, Cat #01249UV-V) and no other strain is mentioned. Yet, in panel A GFP-LC3 is used to demonstrate colocalization with L. monocytogenes during infection. Please comment on this discrepancy. -The conclusion about colocalization of LC3 and -201 on "L. monocytogenes-containing vesicles" (Line 308) appears to be made based on one ROI. Are there additional images and/or quantification that can address (1) the frequency of LC3 association with L. monocytogenes in these infection conditions and (2) the frequency of -201 and LC3-association across multiple infected cells. It seems surprising that LC3 appears to be so strongly associated with the Listeria cells given that they are largely thought to evade autophagy (~10% of L. monocytogenes co-localize with LC3 in macrophage cell lines, for example, but this single ROI suggests a much larger proportion of L. monocytogenes are associated with LC3 in these Caco-2 derivatives). Could this potentially be a factor of what is presumably ARHGEF2 overexpression in this context given that Caco2 cells naturally express more -201 (figure 1C) and are also ectopically expressing 201-RFP? The authors should show data for LC3 association with L. monocytogenes from non-overexpressing Caco-2 cells as controls.
Answer: We have removed the figure. Defining the spatiotemporal recruitment of autophagy regulators will require comprehensive high-resolution imaging, including STED microscopy, as well as the establishment of experimental systems that permit these analyses in primary intestinal organoids instead of tumor-derived cell lines. These studies are beyond the scope of the current manuscript and will be the focus of future work.

The addition of a graphical model summarizing the role of the two separate ARHGEF2 isoforms under "native" conditions and during infection including the proposed localization/proteins complexes would provide additional clarity on the overall proposed model of the isoform switching mechanism.

Answer: We have created a summary figure of the proposed function of ARHGEF2 isoforms in intestinal epithelial cells. Please see Supplementary data figure 5 (Fig. S5).
