# Supplementary material for: ARHGEF2 isoform switching couples intestinal epithelial barrier function with autophagic host defense response to pathogens
Source: Nat Commun. 2026 Sep 24;17:11103. doi: 10.1038/s41467-026-78030-w (PMC13696713; doi:10.1038/s41467-026-78030-w)
Supplement: Supplementary file 3 [file 41467_2026_78030_MOESM1_ESM.pdf]

Supplementary Information:

1. Supplementary Figures

2. Data for STED microscopy: Depletion laser and power settings

1. Supplementary Figures

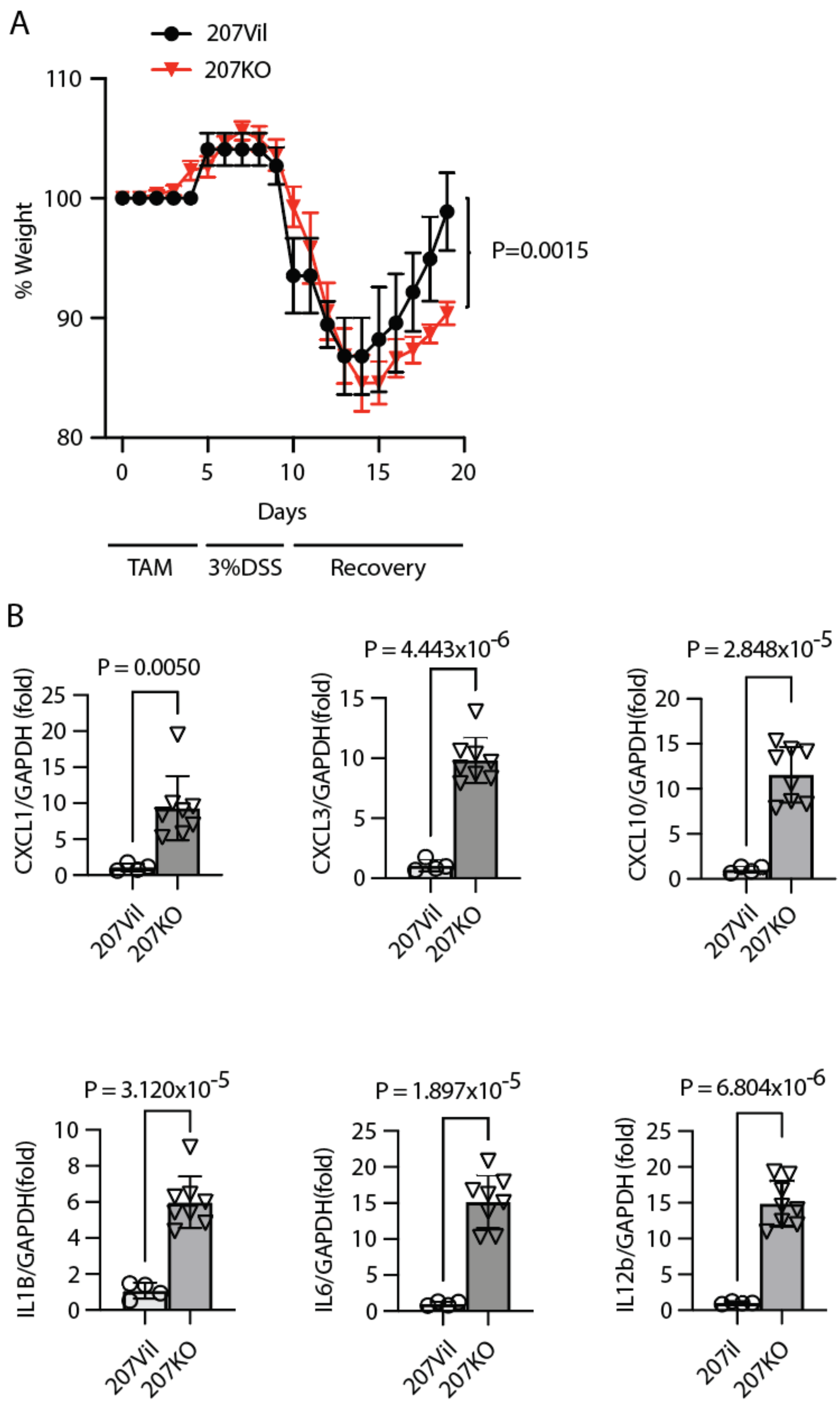

Fig. S1. Arhgef2-207 is required for intestinal epithelial barrier integrity and intestinal homeostasis.

(A). Graph showing body weight change in 207Vil (n = 4) and 207KO (n = 8) mice administered 3% DSS in drinking water for 5 days following tamoxifen intraperitoneal (i.p.) injection.

(B). qPCR quantification of CXCL1, CXCL3, CXCL10, IL1β, IL6, and IL12b expression in mesenteric lymph node (MLN) tissue from 207Vil (n = 4) and 207KO (n = 8) mice treated with 3% DSS. Experiment was independently repeated two times and yielded consistent results. Source data provided in Source Data files.

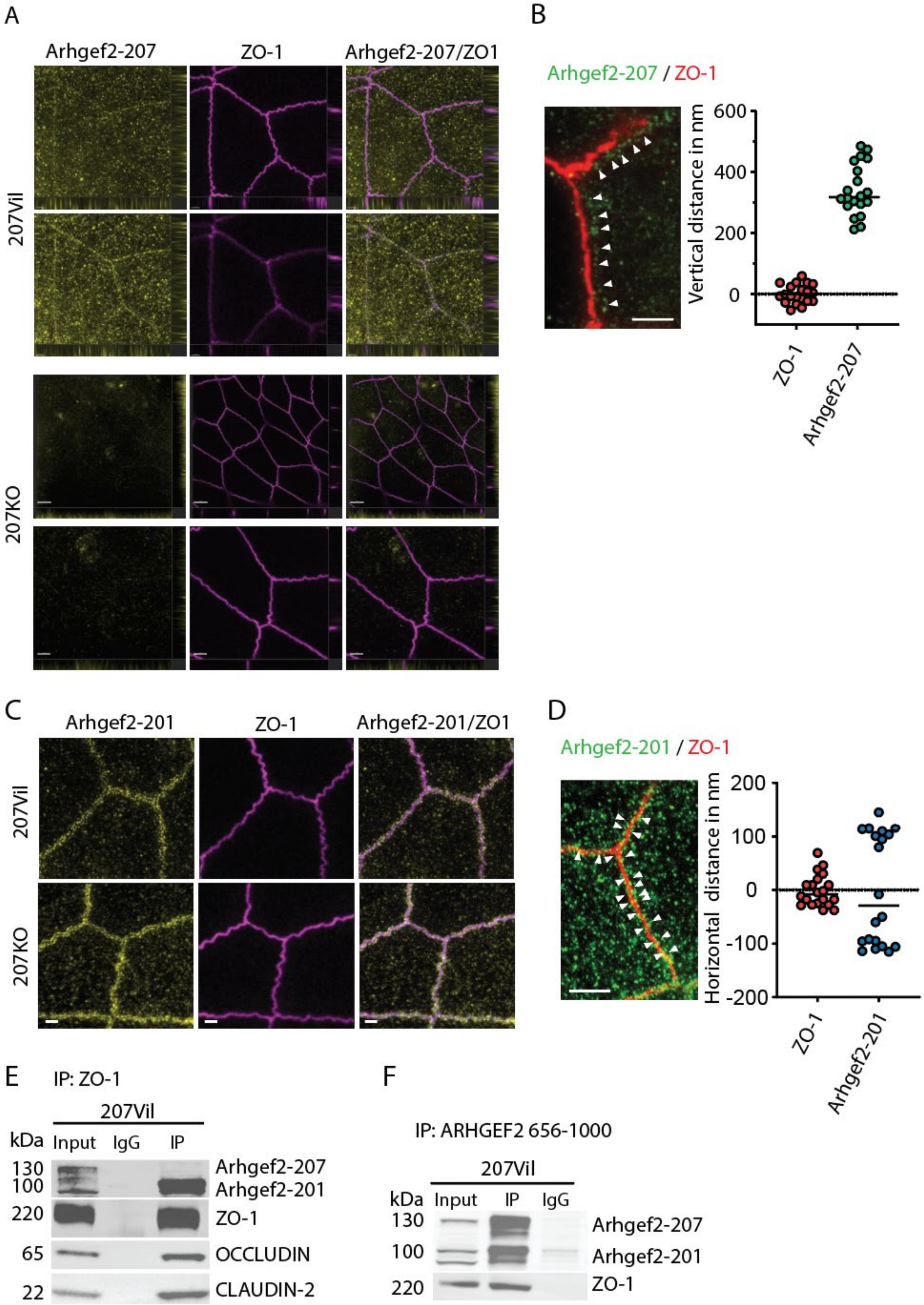

A
Arhgef2-207
ZO-1
Arhgef2-207/ZO1
207Vil
207KO
B
Arhgef2-207 / ZO-1
Vertical distance in nm
600
400
200
0
ZO-1
Arhgef2-207
C
Arhgef2-201
ZO-1
Arhgef2-201/ZO1
207Vil
207KO
D
Arhgef2-201 / ZO-1
Horizontal distance in nm
200
100
0
-100
-200
ZO-1
Arhgef2-201
E
IP: ZO-1
207Vil
kDa
Input
IgG
IP
130
100
220
65
22
Arhgef2-207
Arhgef2-201
ZO-1
OCCLUDIN
CLAUDIN-2
F
IP: ARHGEF2 656-1000
207Vil
kDa
Input
IP
IgG
130
100
220
Arhgef2-207
Arhgef2-201
ZO-1

Fig. S2. Isoform- specific coupling of Arhgef2 isoforms to epithelial junctional protein.

(A). Apical STED image of mouse small intestinal epithelium showing Arhgef2-207 localization at apical junctions. Scale bar, 1 µm.

(B). Lateral STED view of junctional regions in mouse small intestine, with quantification of the distance from the midline of the ZO-1 signal. Arrows indicate puncta labeled by an antibody raised against amino acids 57–76 of Arhgef2-207. Scale bar, 1 µm.

(C). Apical STED image of mouse small intestinal epithelium showing Arhgef2-201 localization at apical junctions. Scale bar, 1 µm.

(D). Lateral STED view of junctional regions in mouse small intestine, with quantification of the distance from the midline of the ZO-1 signal. Arrows indicate puncta labeled by an antibody raised against amino acids 656–1000 of Arhgef2-201. Scale bar, 1 µm.

(E). Immunoprecipitation of ZO-1 and associated protein complexes from intestinal epithelial cell (IEC) lysates of 207Vil mice.

(F). Immunoprecipitation using antibodies against Arhgef2 (amino acids 656–1000) and associated protein complexes from IEC lysates of 207 KO mice. Experiment was independently repeated two times and yielded consistent results. Source data provided as Source Data files below.

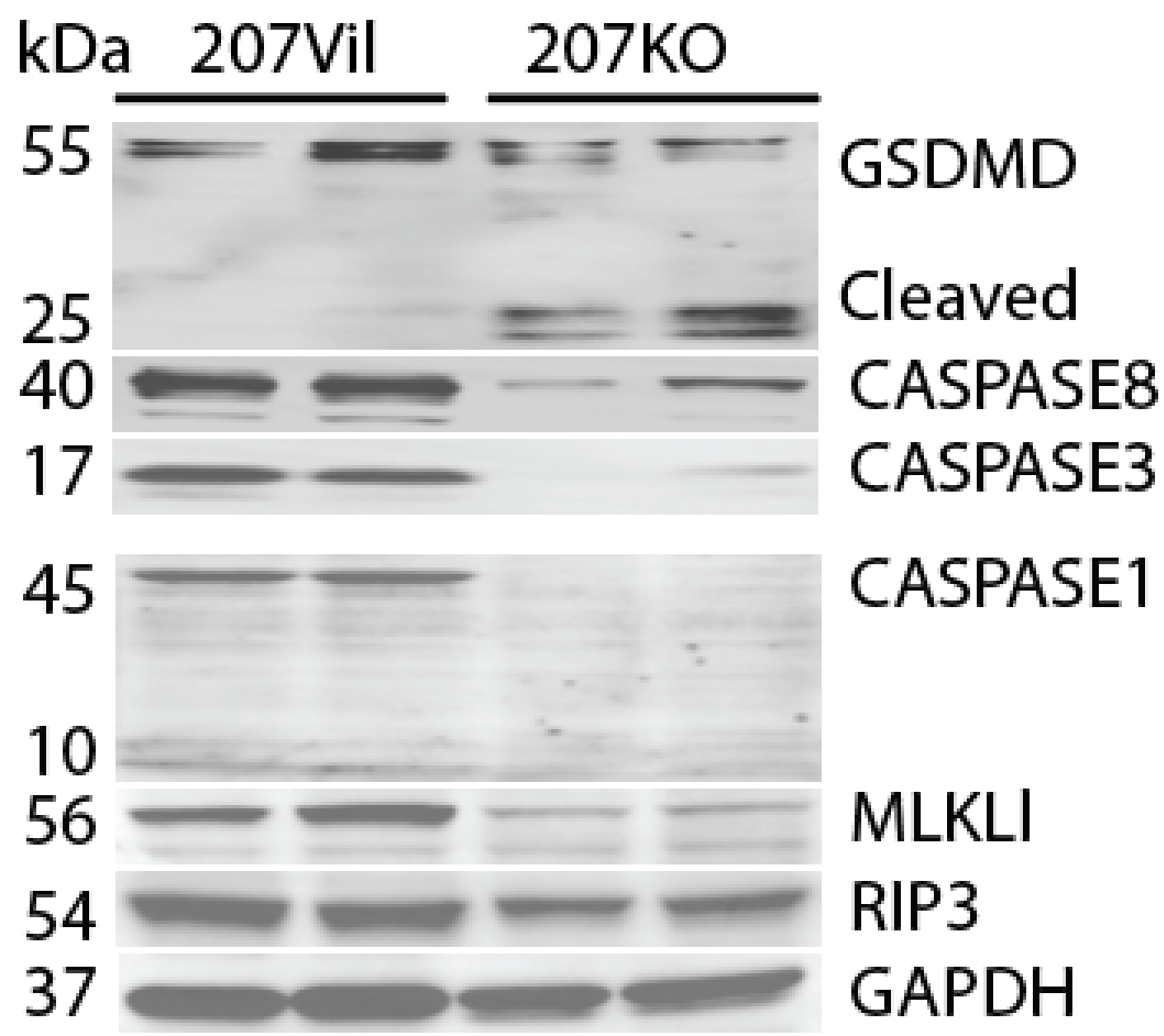

Fig. S3. Caspase 1 activation upon deletion of Arhgef2-207 in IECs.

Immunoblot analysis of apoptosis and pyroptosis markers in IECs lysates from 207Vil mice before and after Cre mediated Arhgef2-207 deletion. Experiment was independently repeated two times and yielded consistent results. Source data provided as Source Data files below.

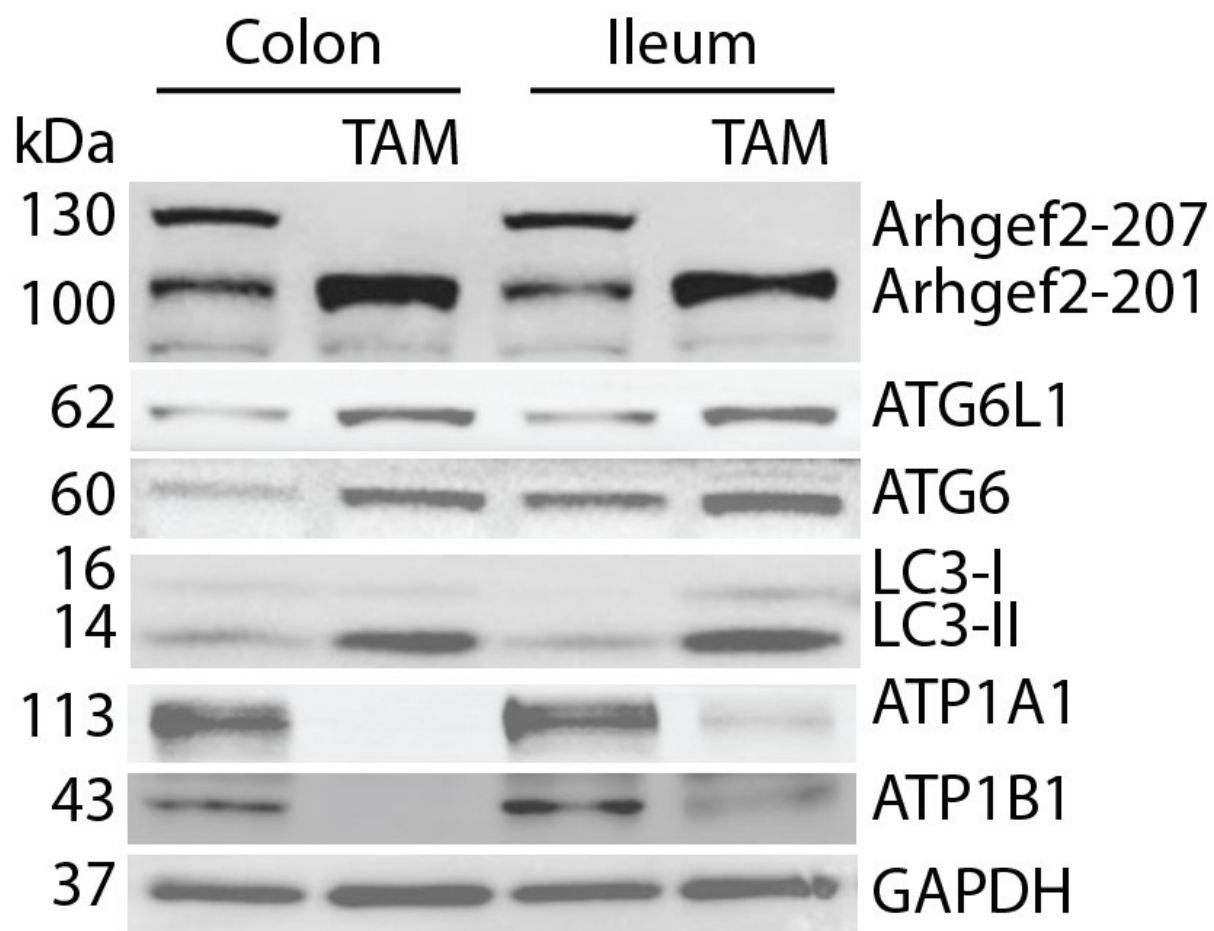

Fig. S4. Induction of polarization and autophagy regulators upon deletion of Arhgef2-207 and induction of Arhgef2-201 in colon and ileum IECs.

Immunoblot analysis of autophagy-related proteins in colon and ileum epithelial organoids derived from 207Vil mice before and after Cre induction, with or without treatment using 10 μM TAM. Experiment was independently repeated two times and yielded consistent results. Source data provided as Source Data files below.

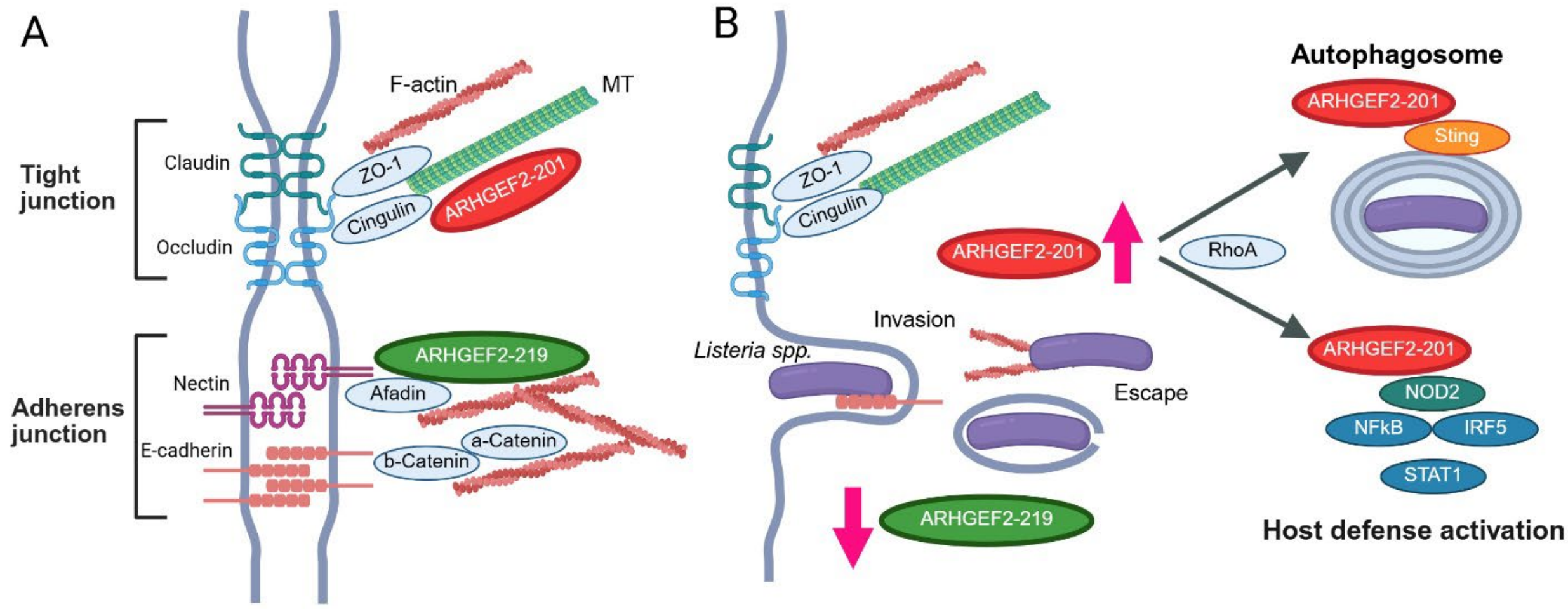

Fig. S5. Proposed model of ARHGEF2 isoform function in intestinal epithelial cells.

(A) The long ARHGEF2 isoform, ARHGEF2-219, is recruited to adherens junctions through association with the Nectin-Afadin complex, where it contributes to epithelial polarization, junctional organization, and barrier integrity. In contrast, the short isoform, ARHGEF2-201, localizes predominantly to tight junctions.

(B) During *Listeria monocytogenes* invasion, disruption of adherens junctions is associated with loss of ARHGEF2-219 expression and induction of ARHGEF2-201. This isoform switch promotes autophagy-associated antimicrobial defense pathways and enhances epithelial innate immune responses, thereby contributing to host protection against infection. Created in BioRender. Reinecker, H. (2026) https://BioRender.com/pvwfq4e".

2. Description of STED microscopy: Depletion laser and power settings

Fig. 3A. Labeled with Abberior STAR RED and Abberior STAR ORANGE was imaged starting at 08:16:14 for 462.2 s using a confocal setup (axes X×Y×Z; image size 1409×519×22 px corresponding to 42.27×15.57×2.75 µm; pixel size 30×30×125 nm; image center X −9.678 mm, Y 1.454 mm, Z 456.5 nm). Imaging was performed with a UPLXAPO60XO objective (60×, NA 1.42, oil immersion). Frame step 1 used a pixel dwell time of 4 µs and a pinhole of 1.0 AU at 574 nm (29.95 µm). For the STAR RED channel: line step 1; data type signed 16-bit integer; single and overlay color red; acquisition mode confocal with accumulation 1; total pixel dwell time 4 µs; excitation at 640 nm with 10% power; detection using APD over 650–755 nm; gating off; Dynamic Plus off.

Fig. 3B. Labeled with Abberior STAR RED and Abberior STAR ORANGE was imaged starting at 09:24:17 for 198.1 s using a confocal setup (axes X×Y×Z; image size 868×251×28 px corresponding to 26.04×7.53×2.8 µm; pixel size 30×30×100 nm; image center X −13.2 mm, Y −908.4 µm, Z −286.6 nm; rotation 34.57°). Imaging was performed with a UPLXAPO60XO objective (60×, NA 1.42, oil immersion). Frame step 1 used a pixel dwell time of 3 µs and a pinhole of 1.0 AU at 574 nm (29.95 µm). For the STAR RED channel: line step 1; data type signed 16-bit integer; single and overlay color red; acquisition mode confocal with accumulation 1; total pixel dwell time 3 µs; excitation at 640 nm with 4% power; detection using APD over 650–755 nm; gating off; Dynamic Plus off

Fig. S2a 207KO. Labeled with Abberior STAR RED and Abberior STAR ORANGE was imaged starting at 08:12:59 for 335.9 s using a confocal setup (axes X×Y×Z; image size 812×945×6 px corresponding to 24.36×28.35×2.4 µm; pixel size 30×30×400 nm; image center X 4.394 mm, Y 3.022 mm, Z −2.602 µm). Imaging was performed with a UPLXAPO60XO objective (60×, NA 1.42, oil immersion). Frame step 1 used a pixel dwell time of 8 µs and a pinhole of 1.0 AU at 574 nm (29.95 µm). For the STAR RED channel: line step 1; data type signed 16-bit integer; single and overlay color red; acquisition mode confocal with accumulation 1; total pixel dwell time 8 µs; excitation at 640 nm with 15% power; detection using APD over 650–755 nm; gating off; Dynamic Plus off.

Fig. S2a 207vil. Labeled with Abberior STAR RED and Abberior STAR ORANGE was imaged starting at 07:18:15 for 423.5 s using a confocal setup (axes X×Y×Z; image size 1020×2316×4 px corresponding to 30.6×69.48×3.2 µm; pixel size 30×30×800 nm; image center X 6.189 mm, Y 3.218 mm, Z 1.591 µm; rotation 4.361°). Imaging was performed with a UPLXAPO60XO objective (60×, NA 1.42, oil immersion). Frame step 1 used a pixel dwell time of 6.5 µs and a pinhole of 1.0 AU at 574 nm (29.95 µm). For the STAR RED channel: line step 1; data type signed 16-bit integer; single and overlay color red; acquisition mode confocal with accumulation 1; total pixel dwell time 6.5 µs; excitation at 640 nm with 15% power; detection using APD over 650–755 nm; gating off; Dynamic Plus off.

Fig. S2b 207vil. Labeled with Abberior STAR RED and Abberior STAR ORANGE was imaged starting at 09:19:21 for 271.9 s using a confocal setup (axes X×Y×Z; image size 331×801×20 px corresponding to 9.93×24.03×4 µm; pixel size 30×30×200 nm; image center X −10.27 mm, Y

1.399 mm, Z 4.602 µm; rotation −20.32°). Imaging was performed with a UPLXAPO60XO objective (60×, NA 1.42, oil immersion). Frame step 1 used a pixel dwell time of 4 µs and a pinhole of 0.91 AU at 574 nm (26.96 µm). For the STAR RED channel: line step 1; data type signed 16-bit integer; single and overlay color red; acquisition mode confocal with accumulation 1; total pixel dwell time 4 µs; excitation at 640 nm with 9% power; detection using APD over 650–755 nm; gating off; Dynamic Plus off.

Fig. S2c 207KO. Labeled with Abberior STAR RED and Abberior STAR ORANGE was imaged starting at 09:36:02 for 184.4 s using a confocal setup (axes X×Y×Z; image size 1018×636×4 px corresponding to 30.54×19.08×2.6 µm; pixel size 30×30×650 nm; image center X 9.683 mm, Y 1.008 mm, Z 3.3 µm; rotation −22.46°). Imaging was performed with a UPLXAPO60XO objective (60×, NA 1.42, oil immersion). Frame step 1 used a pixel dwell time of 8 µs and a pinhole of 1.0 AU at 574 nm (29.95 µm). For the STAR RED channel: line step 1; data type signed 16-bit integer; single and overlay color red; acquisition mode confocal with accumulation 1; total pixel dwell time 8 µs; excitation at 640 nm with 15% power; detection using APD over 650–755 nm; gating off; Dynamic Plus off.

Fig. S2c 207vil. Labeled with Abberior STAR RED and Abberior STAR ORANGE was imaged starting at 09:06:19 for 136 s using a confocal setup (axes X×Y×Z; image size 862×652×4 px corresponding to 25.86×19.56×2.6 µm; pixel size 30×30×650 nm; image center X −4.078 mm, Y −2.99 mm, Z 2.016 µm; rotation 30.37°). Imaging was performed with a UPLXAPO60XO objective (60×, NA 1.42, oil immersion). Frame step 1 used a pixel dwell time of 6.5 µs and a pinhole of 1.0 AU at 574 nm (29.95 µm). For the STAR RED channel: line step 1; data type

signed 16-bit integer; single and overlay color red; acquisition mode confocal with accumulation 1; total pixel dwell time 6.5 μs; excitation at 640 nm with 15% power; detection using APD over 650–755 nm; gating off; Dynamic Plus off.
