# Supplementary material for: ARHGEF2 isoform switching couples intestinal epithelial barrier function with autophagic host defense response to pathogens
Source: Nat Commun. 2026 Sep 24;17:11103. doi: 10.1038/s41467-026-78030-w (PMC13696713; doi:10.1038/s41467-026-78030-w)
Supplement: Supplementary file 4 [file 41467_2026_78030_MOESM2_ESM.pdf]

Description of Additional Supplementary Files

File name: Supplementary Data 1
Description: Peptides detected by mass spectrometry following immunoprecipitation with antibodies targeting amino acids 57–76 of Arhgef2-207 in primary intestinal epithelial cells.

File name: Supplementary Data 2
Description: Peptides detected by mass spectrometry following immunoprecipitation with antibodies targeting amino acids 656–1000 of Arhgef2-201 in primary macrophages.

File name: Supplementary Data 3
Description: Residue-level contact map of the ARHGEF2-219 N-terminal interface with ACTG1 defined by ≤4 Å proximity. For each N219 residue, the corresponding ACTG1 residues within this distance are listed. The minimum interatomic distance (Å) and the number of atom–atom pairs within 4 Å are reported. Residues without detected contacts under this criterion (e.g., P31) are indicated by the absence of ACTG1 partners and zero atom-pair counts. Contact clusters are observed in two discrete regions of the N219 sequence (residues 26–31 and 57–71), consistent with localized interface segments.

File name: Supplementary Data 4
Description: Residue-level contact analysis of the ARHGEF2-219 N-terminal segment (residues 26–71) in the W29A/P31A mutant model reveals a marked loss of interactions within the mutated core RHSAGAG motif. While residues R26 and H27 retain limited contacts with ACTG1 (minimum distances 2.38–3.31 Å), all subsequent residues within the mutated region (28–31) lack detectable contacts within 4 Å, indicating disruption of the local interface.

File name: Supplementary Data 5
Description: Comparative residue-level contact analysis of the wild-type model with the AAFAQAEEGA DTFLGLQ mutant (in residues 55–71) that does not engage ACTG1 and is positioned distal to the interface. In the mutant model, this 55–71 motif fails to establish any detectable contacts within 4 Å, with all residues displaced from the ACTG1 surface despite moderate pLDDT confidence. These data indicate a complete loss of binding capacity within the mutated motif, consistent with disruption of its positioning relative to the ACTG1 interface.

File name: Supplementary Data 6
Description: DESeq2 analysis of differentially regulated genes in small intestinal epithelial cells isolated from mice. Arhgef2-207vil and Arhgef2-207KO.

File name: Supplementary Data 7
Description: Quantification of infection-induced changes in RNA polymerase II (Pol II) loading at promoters. Promoter-specific Pol II CUT&Tag signal (mean ± SEM) across

Arhgef2-associated promoters (P1–P2), as well as CXCL8 and PHDLA1, in control and *Listeria*-infected organoids. Includes Welch's t-test statistics, fold-change values, and $\log_2$ fold-change ($\log_2$FC) estimates.

File name: Supplementary Data 8
Description: Key resources table.
