# Supplementary material for: ARHGEF2 isoform switching couples intestinal epithelial barrier function with autophagic host defense response to pathogens
Source: Nat Commun. 2026 Sep 24;17:11103. doi: 10.1038/s41467-026-78030-w (PMC13696713; doi:10.1038/s41467-026-78030-w)
Supplement: Supplementary file 5 [file 41467_2026_78030_MOESM10_ESM.docx]

**Key resources table**

| REAGENT or RESOURCE | SOURCE | IDENTIFIER | | |
| --- | --- | --- | --- | --- |
| Antibodies | | | | |
| Sheep anti-*Arhgef2* | Exalpha Biologicals | X1089 | | |
| Rabbit anti-GEFH1 | Abcam | ab155785 | | |
| Rabbit anti-*Arhgef2* (57-76) | Pacific Immunology Corp. | PAC17843/4 | | |
| Rabbit anti-*Arhgef2* (6-28) | Pacific Immunology Corp. | PAC17845/6 | | |
| Rabbit anti-GAPDH | Cell Signaling Technology | 92310 | | |
| Mouse anti-ZO-1 | Invitrogen | 33-9100 | | |
| Rat anti-Occludin | Cell Signaling Technology | 68534 | | |
| Rabbit anti-Claudin-2 | Cell Signaling Technology, | 48120 | | |
| Rabbit anti-Claudin-3 | Invitrogen | 34-1700 | | |
| Goat anti-E-Cadherin | R&D Systems | AF748-SP | | |
| Rabbit anti-β-Catenin | Cell Signaling Technology | 8480 | | |
| Goat anti-Nectin-3 | Invitrogen | PA5-47441 | | |
| Goat anti-Nectin-3 | R&D Systems | AF3064-SP | | |
| Rabbit anti-Afadin | Cell Signaling Technology | 13531T | | |
| Rabbit anti-ATP1A1 | Cell Signaling Technology | 3010S | | |
| Rabbit anti-ATP1B1 | Cell Signaling Technology | 44759S | | |
| Mouse anti-α-Tubulin | Cell Signaling Technology | 3873P | | |
| Mouse anti-β-Actin | Cell Signaling Technology | 3700S | | |
| Rabbit anti-IRF5 | Abcam | 181553 | | |
| Rabbit anti-phospho-IRF5 | Invitrogen | PA5-64760 | | |
| Rabbit anti-IKKε | Cell Signaling Technology | 2905P | | |
| Rabbit anti-phospho-IKKε | Cell Signaling Technology | 8766S | | |
| Rabbit anti-STAT1 | Cell Signaling Technology | 9172 | | |
| Rabbit anti-phospho-STAT1 | Cell Signaling Technology | 7649P | | |
| Rabbit anti-p65 | Cell Signaling Technology | 8242S | | |
| Mouse anti-phospho-p65 | Cell Signaling Technology | 13346S | | |
| Rabbit anti-STING | Cell Signaling Technology | 13647S | | |
| Rabbit anti-TBK1 | Cell Signaling Technology | 3504 | | |
| Rabbit anti-phospho-TBK1 | Cell Signaling Technology | 5483S | | |
| Rabbit anti-IL-1B | Cell Signaling Technology | 31202 | | |
| Rabbit anti-ATG16L1 | Cell Signaling Technology | 8089 | | |
| Rabbit anti-BECLIN1 | Cell Signaling Technology | 3495T | | |
| Rabbit anti-LC3 | Cell Signaling Technology | 12741 | | |
| Mouse anti-S6 | Cell Signaling Technology | 2317 | | |
| Rabbit anti- phospho-S6 | Cell Signaling Technology | 4858 | | |
| Rabbit anti-p70S6K | Cell Signaling Technology | 34475T | | |
| Mouse anti-phospho-p70S6K | Cell Signaling Technology | 9206T | | |
| Rabbit anti-p62 | Cell Signaling Technology | 5114 | | |
| Rabbit anti-MLKL | Cell Signaling Technology | 14993S | | |
| Rabbit anti-GSDMD | Proteintech, | 20770-1-AP | | |
| Rabbit anti-Caspase-1 | Abcam | Ab108362 | | |
| Rabbit anti-cleaved Caspase-3 | Cell Signaling Technology | 9664 | | |
| Rabbit anti-Caspase-8 | Cell Signaling Technology | 4790S | | |
| Rabbit anti-RIP3 | Cell Signaling Technology | 3493S | | |
| Rabbit anti-phospho-ROCK1 | Invitrogen | BS-4630R | | |
| Anti-rabbit IgG-HRP | Cell Signaling Technology | 7074 | | |
| Anti-mouse-IgG-HRP | Cell Signaling Technology | 7078 | | |
| Anti-sheep-IgG-HRP | Invitrogen | A16041 | | |
| Anti-goat-IgG-HRP | Santa Cruz Biotechnology | SC-2354 | | |
| Anti-goat IgG AF488 | Invitrogen | A32814TR | | |
| Anti-rabbit IgG AF594 | Invitrogen | A11037 | | |
| Anti-mouse IgG AF488 | Invitrogen | A-21202 | | |
| Anti-goat IgG AF647 | Invitrogen | A-21447 | | |
| STAR Red goat anti-rabbit | Abberior Instruments | STRED-1002-500UG | | |
| STAR Red donkey anti-goat | Abberior Instruments | STORANGE-1055-500UG | | |
| **Bacterial and virus strains** |  |  | | |
| One ShotTop10 Competent E. coli | Invitrogen | C404003 | | |
| Listeria monocytogenes | Microbiologics | 01249UV-V | | |
| **Chemicals, peptides, and recombinant proteins** |  |  | | |
| Fetal Bovine Serum | Gibco | 26140-079, 500 mL | | |
| 4-Hydroxytamoxifen | MilliporeSigma | H6278 | | |
| Tamoxifen | MilliporeSigma | T2859 | | |
| Penicillin/streptomycin | Sigma-Aldrich | P4458-100mL | | |
| RPMI-1640 Medium (no L-glutamine) | Sigma-Aldrich | RO883-500mL | | |
| DMEM, High Glucose (with L-Glutamine, Sodium Pyruvate) | Cytiva (Hyclone) | SH30243-01 | | |
| 0.25% Trypsin | Gibco | 25200-056 100mL | | |
| 70 µm Cell Strainers | Falcon | 352350 | | |
| 10 cm Petri Dish | Fisherbrand | FB0875713 | | |
| 15 mL Tube | Falcon | 352097 | | |
| 50 mL Tube | Falcon | 352098 | | |
| 1.5 mL Microcentrifuge Tube | Eppendorf | 022363204 | | |
| 384-well PCR Plates | Bio-Rad | HSP3805 | | |
| PCR Film | Eppendorf | 0030127781 | | |
| PCR 8-tube Strips | USA Scientific | 21102 | | |
| 6-well Plate | Corning | 3506 | | |
| 10-cm Culture Dish | Corning | 430167 | | |
| PBS (tablet) | Millipore Sigma | 524650 | | |
| Pierce RIPA Buffer | Thermo Scientific | 89900 | | |
| Proteinase Inhibitor Cocktail | Sigma-Aldrich | P8340-1mL | | |
| Tween 20 | Fisher Scientific | BP337-500 | | |
| 10X Tris/Glycine Buffer | Bio-Rad | 1610771 | | |
| 10X Tris/Glycine/SDS Buffer | Bio-Rad | 1610772 | | |
| Methanol | Sigma-Aldrich | 179337-500mL | | |
| 8-16% Tris-Glycine Gel | Invitrogen | XP08162BOX | | |
| 4-12% Tris-Glycine Gel | Invitrogen | NW04122BOX | | |
| Immobilon Western Chemiluminescent HRP Substrate | Millipore | WBKLS0100 | | |
| Pierce IP Lysis Buffer | Thermo Scientific | 87787 | | |
| Bio-Rad XT Sample Buffer X4 | Bio-Rad | 1610791 | | |
| 10% SDS | Invitrogen | 15553-035 100mL | | |
| 50X TAE Buffer | Invitrogen | 24710-030 1000mL | | |
| Luria Agar | Research Product International | L24020-500 | | |
| Luria Broth | Research Product International | L24040-1000 | | |
| ProLong Gold Antifade Mountant with DAPI | Invitrogen | P36931 | | |
| Normal Donkey Serum | Jackson ImmunoResearch | NC9624464 | | |
| Matrigel Matrix | Corning | 356231 | | |
| GlutaMAX | Gibco | 35050 | | |
| Advanced DMEM/F12 Medium | Gibco | 12634-010 | | |
| Y-27632 Dihydrochloride | Selleck Chemicals | 1254 | | |
| SB 431542 | TOCRIS | 1614 | | |
| Antibiotic-Antimycotic | Gibco | 15240-062 | | |
| 24-well Nunclon Delta Surface Plate | Thermo Fisher Scientific | 142475 | | |
| SAR405 | Sigma-Aldrich | AMBH303C4EB3 | | |
| Wortmannin | Sigma-Aldrich | 681675 | | |
| Bafilomycin A1 | Sigma-Aldrich | AABH30371C0C | | |
| H-151 | Sigma-Aldrich | SML2437 | | |
| **Critical commercial assays** |  |  | | |
| RNeasy Plus Mini RNA Kit | Qiagen | 74134 | | |
| QuantiTect Reverse Transcription Kit | Qiagen | 205311 | | |
| EndoFree Plasmid Maxi Kit | Qiagen | 12362 | | |
| PowerTrack SYBR Green Master Mix | Applied Biosystems | A46109 | | |
| Primescript RT Reagent Kit | TaKaRa | RR037a | | |
| Pierce BCA Protein Assay Kit | Thermo Fisher Scientific | 23227 | | |
| Lipofectamine 3000 Transfection Reagent | Thermo Fisher Scientific | L3000001 | | |
| CellTiter-Glo Luminescent Cell Viability Assay | Promega | G7570 | | |
| **Experimental models: Cell lines** |  |  | | |
| ARPE-19 | ATCC | CRL-2302 | | |
| Caco2 | ATCC | HTB-37 | | |
| T84 | ATCC | CCL-248 | | |
| HCT116 | ATCC | CCL-247EMT | | |
| HT29 | ATCC | HTB-38 | | |
| **Experimental models: Organisms/strains** |  |  | | |
| ARHGEF2-/- mice | https://www.ncbi.nlm.nih.gov/pmc/articles/PMC4066330/ | N/A | | |
| C57BL/6 | The Jackson Laboratory | 000664 | | |
| B6.Cg-Tg(Vil1-cre/ERT2)23Syr/J | The Jackson Laboratory | 020282 | | |
| *Arhgef2*-201 flox mice | See Methods | N/A | | |
| *Argef2-*207 flox mice | See Methods | N/A | | |
| **Oligonucleotides** |  |  | | |
| *Arhgef2*-lox2-F | AGGGCTGTGGAATTCTCAGC | | N/A |  |
| *Arhgef2*-lox2-R | AGAGCCCTGTGTAGACCCTT | | N/A |  |
| *Arhgef2*-lox4-F | CCCGGCACAGAGAAATCGTA | | N/A |  |
| *Arhgef2*-lox4-R | ACCTGGCGTCCAGATAAACA | | N/A |  |
| *Arhgef2*-lox5-F | TGCCATGCTTGGATAGCACA | | N/A |  |
| *Arhgef2*-lox5-R | GCAAGGATGCTTCCCACAAG | | N/A |  |
| *Arhgef2*-207-F | GCTTCCACACATCACTCCTTAG | | N/A |  |
| *Arhgef2*-207-R | GCAGTCTCCTCCTGCTTATTG | | N/A |  |
| *Arhgef2*-201-F | TTGCAGATGGAGCCAGAAG | | N/A |  |
| *Arhgef2*-201-R | GCCGTTCCAGAAGCTGATTA | | N/A |  |
| *ARHGEFH2*-201-F | CTGTACCAAGGTCAAGCAGAAG | | N/A |  |
| *ARHGEF2*-201-R | TCCCGGATGGTTGTCTTACT | | N/A |  |
| *ARHGEF2*-219-F | ATGCTTTCCAAGTCTGTACCC | | N/A |  |
| *ARHGEF2*-219-R | CAGCGTTTCCTGAGGTCAAT | | N/A |  |
| oIMR1084 | GCGGTCTGGCAGTAAAAACTA TC | | N/A |  |
| oIMR1085 | AAACAGCATTGCTGTCACTT | | N/A |  |
| oIMR7338 | AGAATTGAAAGATCT | | N/A |  |
| IL6-F | CTTCACAAGTCGGAGGCTTAAT | | N/A |  |
| IL6-R | ACTCCAGGTAGCTATGGTACT | | N/A |  |
| IL12b-F | GGACCAAAGGGACTATGAGAAG | | N/A |  |
| IL12b-R | GAAGAAGCTGGTGCTGTAGT | | N/A |  |
| IFNb-F | GATGACGGAGAAGATGCAGAAG | | N/A |  |
| IFNb-R | ACCCAGTGCTGGAGAAATTG | | N/A |  |
| GAPDH -F | GGAGAAACCTGCCAAGTATGA | | N/A |  |
| GAPDH -R | CCTGTTGCTGTAGCCGTATT | | N/A |  |
| **Recombinant DNA** |  | |  |  |
| pCMV6-AC-GFP | Origene | | PS100010 |  |
| pCMV6-AC-RFP | Origene | | PS100034 |  |
| Arhgef2 (NM_008487) Mouse Tagged ORF Clone | Origene | | MG211388 |  |
| pCMV-human Arhgef2-GFP/RFP | PubMed 30902986 | | N/A |  |
| pCMV-human Arhgef2S372A-GFP | PubMed 30902986 | | N/A |  |
| **Software and algorithms** |  | |  |  |
| Excel | Microsoft Office | | N/A |  |
| GenePix Pro 7 | Molecular Devices | | N/A |  |
| GraphPad Prism 9 | GraphPad | | N/A |  |
| MestReNova | Mestrelab Research S.L. | | N/A |  |
| ImageJ | NIH / Wayne Rasband | | RRID:SCR_003070 |  |
| Imaris | Oxford Instruments / Bitplane | | <https://imaris.oxinst.com/> |  |
| **Other** |  | |  |  |
| Slides, Nexterion Slide H | Schott Minifab | | N/A |  |
| 16-Well Slide Module | Grace Bio-Labs | | 206862 |  |
| Stainless Steel Spring Clips | Grace Bio-Labs | | 204838 |  |
| MicroAmp Optical Adhesive Film | Applied Biosystems | | 4311971 |  |
| FoilSeal | Excel Scientific | | XTRFOIL100 |  |
| RNASeq data | <https://www.ncbi.nlm.nih.gov/geo/query/acc.cgi?acc=>GSE311695. | |  |  |
| Resource data file | <https://doi.org/10.18738/T8/OMPEZK> | |  |  |
